# Deamination of 1-Aminoalkylphosphonic Acids: Reaction Intermediates and Selectivity

**DOI:** 10.3390/molecules27248849

**Published:** 2022-12-13

**Authors:** Anna Brol, Tomasz K. Olszewski

**Affiliations:** Department of Physical and Quantum Chemistry, Faculty of Chemistry, Wrocław University of Science and Technology, ul. Wybrzeże Wyspiańskiego 27, 50-370 Wrocław, Poland

**Keywords:** diazotization, carbenium ion, 1-phosphonoalkylium ion, substitution reaction, elimination reaction, rearrangement

## Abstract

Deamination of 1-aminoalkylphosphonic acids in the reaction with HNO_2_ (generated “in situ” from NaNO_2_) yields a mixture of substitution products (1-hydroxyalkylphosphonic acids), elimination products (vinylphosphonic acid derivatives), rearrangement and substitution products (2-hydroxylkylphosphonic acids) as well as H_3_PO_4_. The variety of formed reaction products suggests that 1-phosphonoalkylium ions may be intermediates in such deamination reactions.

## 1. Introduction

Organophosphorus compounds are a very interesting class of molecules well known to exist in nature, exhibit very intriguing activity, and have already found broad applications in various sectors of industry, such as in agrochemistry [1], pharmacy [2], catalysis [3], materials [4], as flame retardants [5], or chemical reagents [6]. Particular interest is devoted to substituted 1-aminoalkylphosphonic acids that can be considered structural analogs of natural 2-aminoalkanoic acids [7,8,9]. In that regard, the use of 1-aminoalkylphosphonic acids in drug discovery has proven successful in many cases, with prominent examples being potential drugs for the treatment of diabetes [10,11], asthma [12], inflammation [13], heart failure [14], cancer [15], malaria [16], and HIV [17]. Due to the importance of the 1-aminoalkylphosphonic acids, several synthetic methods for their preparation have been designed over the years [18,19,20,21,22,23,24,25,26].

**Scheme 1:**
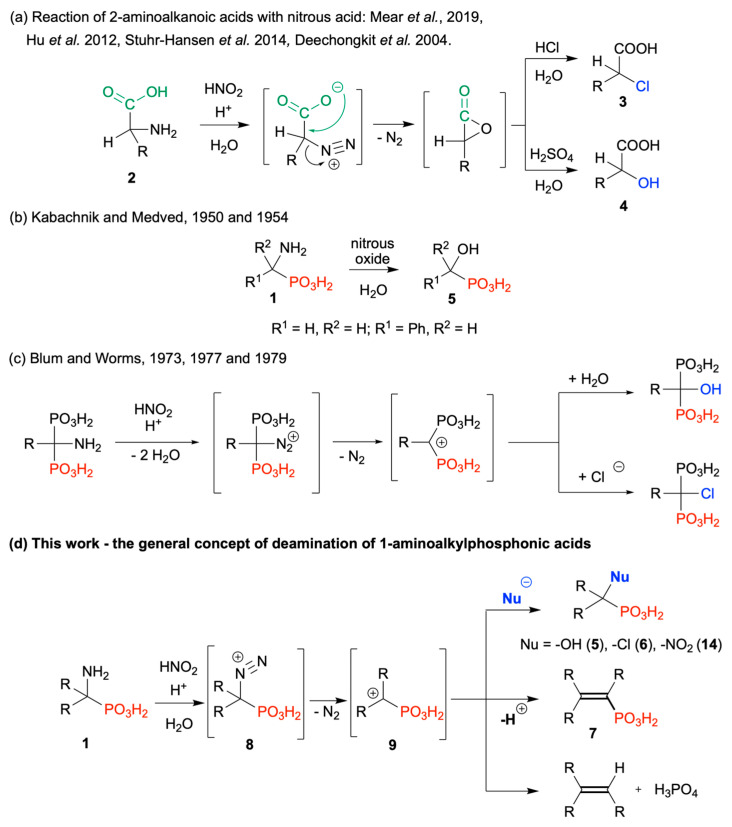
General presentation of deamination reaction carried out on 2-aminoalkanoic acids and their phosphorus analogs [34,35,36,37,49,50,51,52,53].

Surprisingly, further transformations of 1-aminoalkylphosphonic acids and their reactivity as reaction substrates in organic synthesis are scarcely described in the literature [27,28,29,30,31,32,33]. Those described include among others alkaline deacylation of 1-(acylamino)alkylphosphonic acids, ref. [31] oxidative dephosphorylation of 1-aminoalkylphosphonic acids [32], oxidation of 1-(*N*,*N*-dialkylamino)-alkylphosphonic acids leading to corresponding N,N-dialkyl-N-oxide derivatives [30], or recently effective preparation of 1-aminoalkylphosphonic acid quaternary ammonium salts from simple 1-aminoalkylphosphonic acid [27]. On the other hand, the use of analogous 2-aminoalkanoic acids as substrates, particularly in diazotization reaction, is a well-known methodology that yields 2-hydroxyalkanoic acids or 2-chloroalkanoic acids (Scheme 1a) [34,35,36,37], useful building blocks in medicinal chemistry [38,39,40], total synthesis of natural products [41,42,43], and polymer chemistry [44,45,46]. Inspired by the activity and utility of 2-amino acids in diazotization reactions we decided to study the reactivity of 1-aminoalkylphosphonic acids in deamination by the diazotization reaction (Scheme 1d). It is well known that the amine group reacts with nitrous acid (HNO_2_) generated by the acidification of aqueous solutions of sodium nitrite (NaNO_2_) with a mineral acid to yield diazonium salts, followed by reaction with various nucleophiles [47]. However, aliphatic diazonium salts are commonly unstable, and the formation of carbenium ion intermediate is inevitable, which causes complications with controlling the selectivity of such reaction [47,48].

**Scheme 2:**
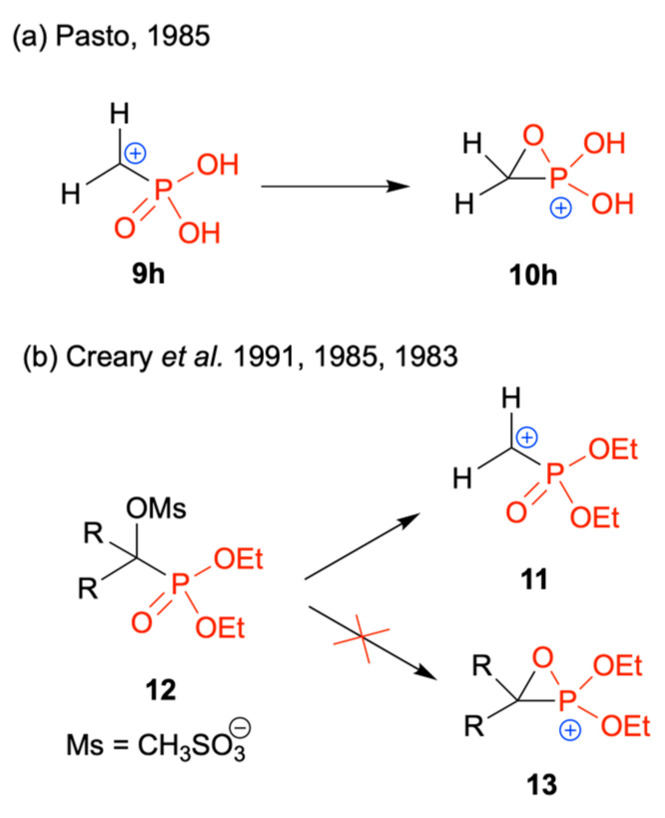
Possible structures of the 1-phosphonoalkylium ions known from the literature [54,55,56,57,58].

While the deamination of 2-aminoalkanoic acids as substrates has been described in a great number of articles, reactions of structurally similar 1-aminoalkylphosphonic acids are scarcely described in the literature. In 1950 [49] and 1954 [50], Kabachnik and Medved described the analytical applications of the deamination reaction of aminomethylphosphonic acid and 1-amino-1-phenylethylphosphonic acid with nitrous oxides in which hydroxymethylphosphonic acid and 1-hydroxy-1-phenylethylphosphonic acids were formed respectively (Scheme 1b). Much later, reactions of related aminoalkylidene-1,1-diphosphonic acids with nitrous acid were described by Blum and Worms [51,52,53]. The authors concluded that carbenium ions with two phosphonic groups are formed and the reaction products are hydroxyalkylidene-1,1-diphosphonic acids, chloroalkylidene-1,1-diphosphonic acids, and derivatives of vinylphosphonic acid (Scheme 1c). It is worth mentioning that 1-phosphonoalkylium ions **9**, which may be intermediates in the deamination reaction of 1-aminoalkylphosphonic acids **1**, have also not been extensively studied in the literature. Only theoretical calculations for the simplest phosphonomethylium ion (**9h**) (which exist in the cyclic form **10h**) have been described by Pasto (Scheme 2a) [54]. On the other hand, Creary et al. studied the formation of carbenium ions substituted with phosphonic ester group **11** in the solvolysis reactions of mesylates **12** [55,56,57,58]. Experiments on the α-deuterium isotope effect proved that intermediates have an open form **11** and that no cyclic compounds **13** are formed (Scheme 2b). Intrigued by the very scarce literature reports on the deamination of 1-aminoalkylphosphonic acids, and interested in revealing the reactivity of the 1-phosphonoalkylium ions, possible intermediates in deamination of 1-aminoalkylphosphonic acids, we decided to study this interesting reaction in greater detail (Scheme 1d).

Herein we present the results of our detailed study on the deamination reaction of structurally diverse 1-aminoalkylphosphonic acids carried out with nitrous acid. The presented results show the potential application of this transformation in organic synthesis and shed light on the possible reaction mechanism and reaction intermediates.

## 2. Results

**Figure 1:**
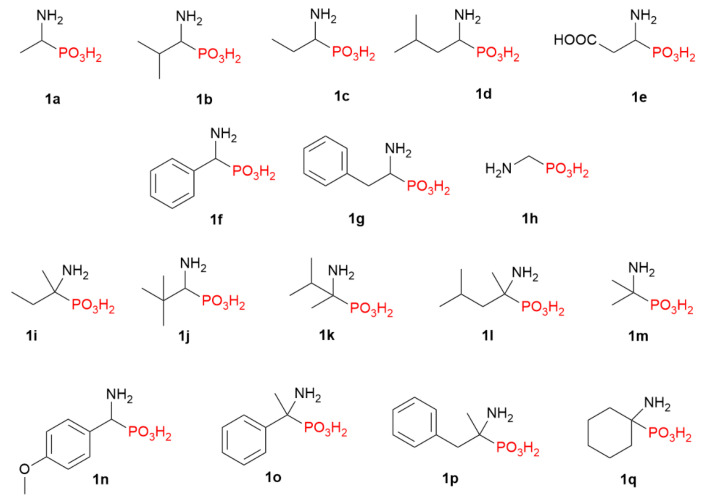
Structures of 1-aminoalkylphosphonic acids used in this study.

For our study, we selected a representative and structurally diverse palette of 1-aminoalkylphosphonic acids (Figure 1, 17 examples). The selected examples include phosphorus analogs of such amino acids as alanine **1a**, valine **1b**, leucine **1d**, glycine **1h**, phenylalanine **1g,** and phenylglycine **1f**.

### 2.1. Diazotization of 2-Aminoalkanoic Acids vs. ***1***-Aminoalkylphosphonic Acids—Preliminary Experiments

**Scheme 3:**
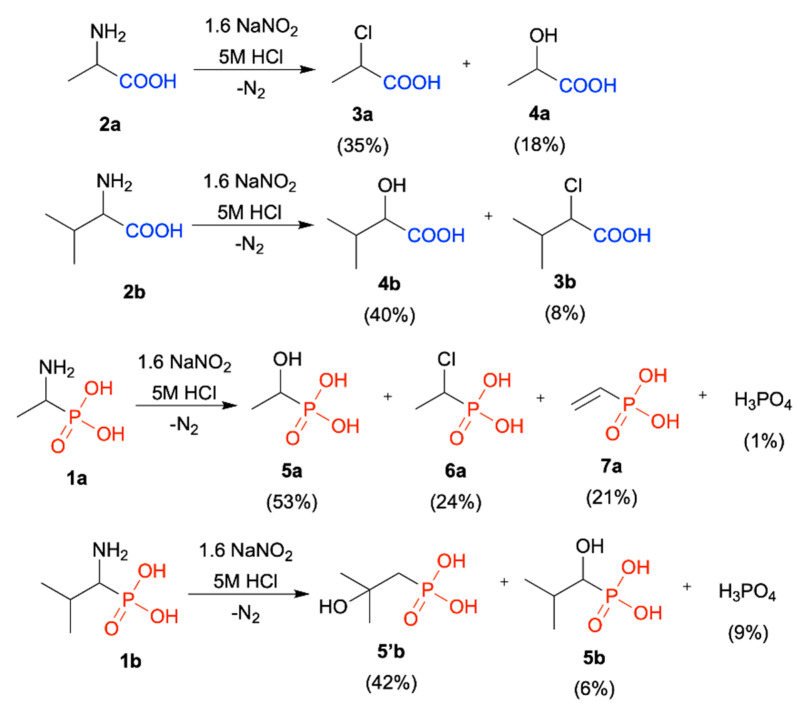
Preliminary experiments on **the** diazotization of alanine (**2a**) and valine (**2b**) and their corresponding phosphorus analogs **1a** and **1b**.

We started our preliminary experiments using the conditions applied for the diazotization of 2-aminoalkanoic acids (NaNO_2_, 5M HCl) (Scheme 3) [59]. Preliminary experiments clearly showed that 1-aminoalkylphosphonic acids reacted with nitrous acid (HNO_2_), generated in situ from sodium nitrite (NaNO_2_), differently than the tested amino acids. The degree of conversion in the case of 1-aminoalkylphosphonic was slightly higher than in the case of classical amino acids. No other products than the ones depicted on Scheme 3 were observed and they were additionally accompanied by unreacted starting material. Under the examined conditions, no selectivity towards chloride ions was observed and 1-hydroxyalkylphosphonic acids were the main reaction products.

Moreover, in the case of amino acids **2**, as expected, the main reaction products were 1-hydroxy or 1-chloroalkanoic acids, while in the case of 1-aminoalkylphosphonic **1** a greater number of reaction products, including rearrangement and fragmentation products, were observed (Scheme 3).

Due to the complex composition of post-reaction mixtures, we decided to modify the original reaction conditions used for the diazotization of amino acids. Expecting to obtain complex reaction mixtures, we wanted to focus first on generating the carbenium ions and then observe their reactivity with just a limited number of nucleophiles to simplify the analysis of the results. Based on the literature data describing the diazotization of amino acids, we envisaged that the most important parameter is the initial pH of the reaction mixture [60,61,62,63]. Lowering the pH should increase the concentration of the electrophilic nitrosating agent, but at the same time causes the protonation of the amino group in the starting 1-aminoalkylphosphonic acids, which lowers the nucleophile concentration. Additionally, we have assumed that 1-aminoalkylphosphonic acids are strong enough acids to generate the nitrosating agent in situ from sodium nitrite in water, therefore there is no need to use hydrochloric acid in the reaction. After this simplification, the only nucleophiles in the reaction mixture were nitrite ions and water.

### 2.2. Diazotization of 1-Aminoalkylphosphonic Acids—Optimized Reaction Conditions

When 1-aminoalkylphosphonic acid **1** (1 equiv.) was added to the solution of NaNO_2_ (2 equiv.), nitrogen evolution was observed, which proved that diazonium salts **8** were generated. The post-reaction mixtures contained products of substitution reaction (1-hydroxyalkylphosphonic acids **5**), elimination reaction (vinylphosphonic acid derivatives **7**), and additionally 2-hydroxyalkylphosphonic acids **5′** and phosphoric acid (H_3_PO_4_).

We have observed that the product distribution in these reactions depended strongly on the structure of the starting 1-aminoalkylphosphonic acid **1**, therefore the reaction results are outlined in Table 1, Table 2, Table 3 and Table 4, according to the structure of the substrates used.

To avoid the formation of secondary products, the crude post-reaction mixtures were analyzed directly by NMR spectroscopy without isolation of the reaction products, and thus the results are given in the form of conversion. In all cases, the structures of reaction products were confirmed by NMR spectroscopy (especially ^31^P NMR and ^1^H NMR) by the addition of known reference compounds (synthesized separately) or by analysis and comparison of the NMR spectra of the crude reaction mixture with spectra of products known from the literature (see Supplementary Materials for more details).

Substitution was generally the main reaction for most of the investigated 1-aminoalkylphosphonic acids **1** (Table 1 and Table 2), especially for those that do not have protons in the β-position (**1f, 1n, 1h**). For example, in the reaction of amino(phenyl)methylphosphonic acid (**1f**) the conversion of substrate to hydroxy(phenyl)phosphonic acid (**5f**) was 97% (Table 2, entry 1).

In turn, elimination was the major reaction for 1-aminoalkylphosphonic acids **1q, 1l,** and **1i** which have bulky substituents (Table 3). For substrates **1l** and **1i,** two isomers of vinylphosphonic acid derivatives were formed: **7l**, **7′l** for **1l** and **7i**, **7′i** for **1i**. We assume that in this case the steric hindrance impedes the access of nucleophiles and, as a result, the elimination reaction is favored.

Furthermore, for substrates **1j, 1b,** and **1k** that have β-position migrating groups, the major reaction product was phosphoric acid (H_3_PO_4_), accompanied by various amounts of substitution products **5** and rearrangement products **5′**.

**Scheme 4:**
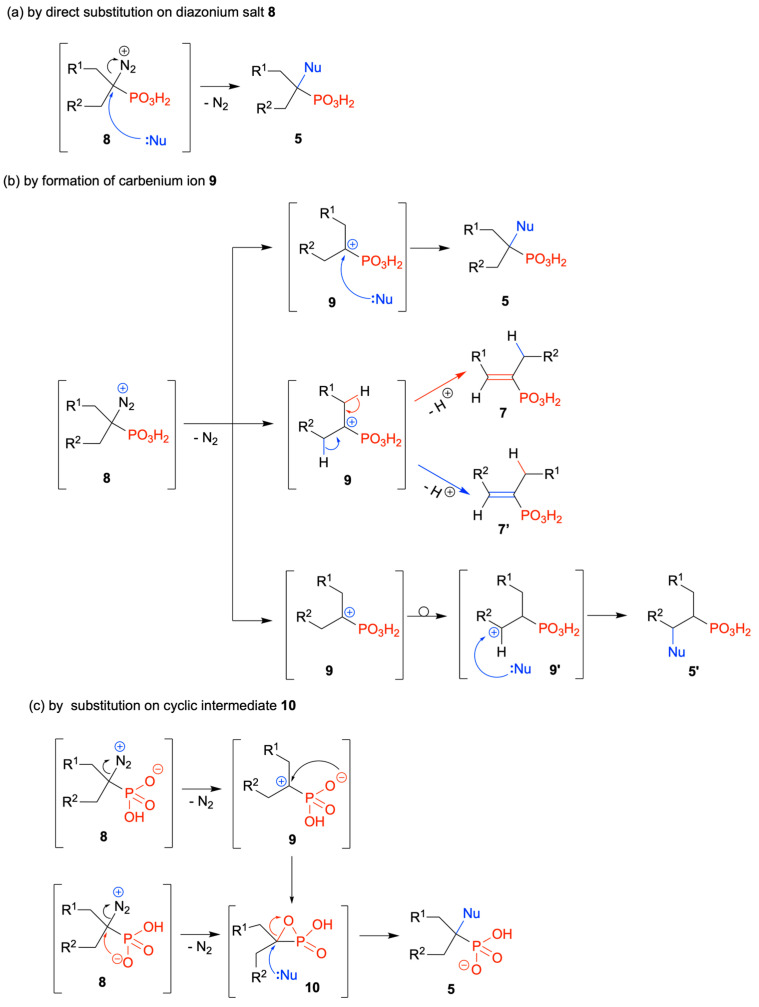
Possible mechanism of the reaction of 1-aminoalkylphosphonic acids **1** with HNO_2_.

**Scheme 5:**
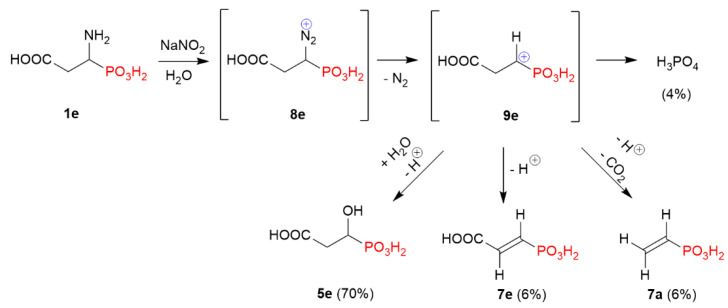
Deamination reaction of 3-amino-3-phosphonopropanoic acid (**1e**) with HNO_2_.

While direct substitution on the diazonium group in 1-phosphonoalkenediazonium salts **8** cannot be excluded (Scheme 4a), the complex composition of the post-reaction mixtures suggests that 1-phosphonoaklylium ions **9** may be intermediates in the diazotization reaction of 1-aminoalkylphosphonic acids **1**. This assumption is supported by the fact that all typical products of carbenium ion reactions, especially rearrangement products **5′**, were observed simultaneously in the crude post-reaction mixtures (Scheme 4b). It has to be mentioned that the accepted mechanism of deamination of analogous aliphatic 2-aminoalkanoic acids assumes the presence of α-lactones as reaction intermediates (Scheme 1a). As postulated, their formation is the reason for the high enantioselectivity of these reactions. By analogy, in the reaction of 1-aminoalkylphosphonic acids similar cyclic intermediates, namely 2-hydroxy-2-oxa-1,2-oxaphosphiranes **10**, could also be postulated (Scheme 4c). However, there is no experimental information about intermediate **10** described thus far in the literature. In addition, our results indicate that the formation of **10** is unlikely. For example, the reaction products of 3-amino-3-phosphonopropanoic acid (**1e**) with nitrous acid may be explained by the assumption that 1-phosphonoalkylium ion **9e** is formed (Scheme 5). The 3-hydroxy-3-phosphonopropanoic acid (**5e**) is formed in the reaction of nucleophile (water) addition to 1-phosphonoalkylium ion **9e**, while *(E)*-3-phosphonoacrylic acid (**7e**) is formed as the result of proton elimination from **9e**. Carbenium ion **9e** also undergoes fragmentation and as a result, vinylphosphonic acid (**7a**) and carbon dioxide are formed.

**Scheme 6:**
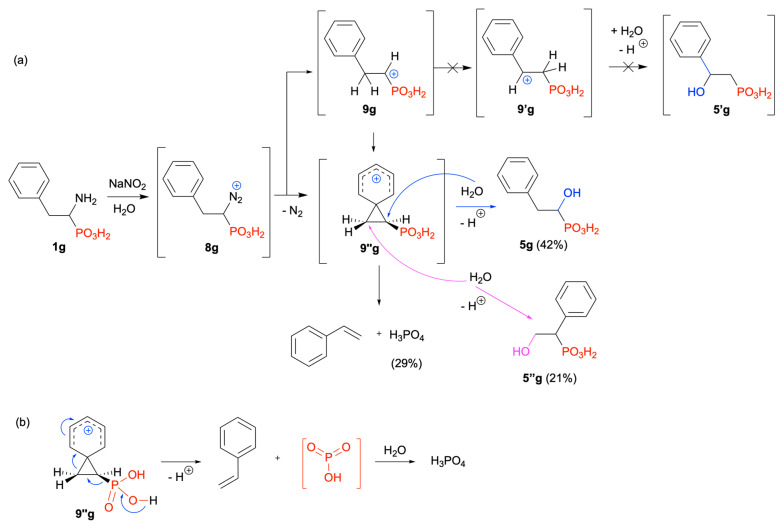
(**a**) Deamination of 1-amino-2-phenylethylphosphonic acid (**1g**) with HNO_2_ and analysis of the reaction products. (**b**) Mechanism of phosphoric acid formation from cyclic intermediate **9″g**.

An interesting example illustrating the complexity of the deamination reaction of 1-aminoalkylphosphonic acid **1** is the reaction of 1-amino-2-phenylethylphosphonic acid (**1g**) with HNO_2_ (Scheme 6). Among the expected products of substitution **5g**, elimination **7g,** and phosphoric acid, in the post-reaction mixture, the rearranged 2-hydroxy-1-phenylethylphosphonic acid (**5″g**) was identified. Considering the formation of carbenium ion **9g** we expected the rearrangement of this carbenium ion to **9′g**, which should be more stable due to the stabilizing effect of the phenyl group. Subsequent addition of nucleophile (H_2_O) to both carbenium ions should lead to the corresponding hydroxyalkylphosphonic acids **5g** and **5′g** respectively (Scheme 6). However, analysis of the NMR spectra of the crude reaction mixture revealed that the second product of the reaction is not the **5′g** but **5″g** (see Supplementary Materials for more details).

**Scheme 7:**
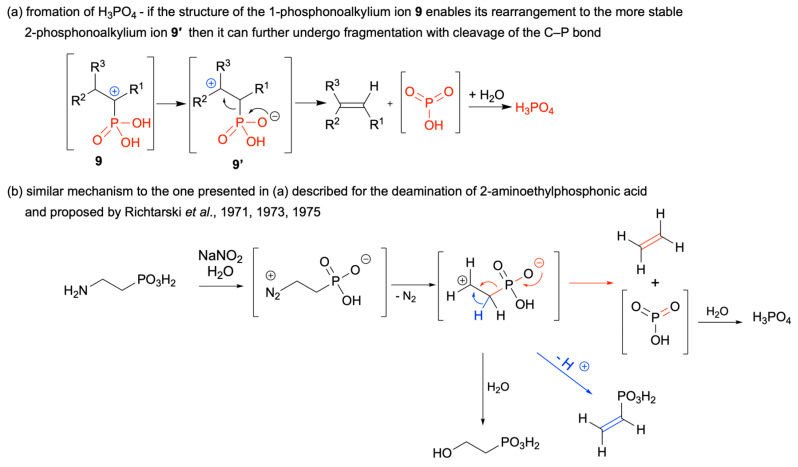
Mechanism of H_3_PO_4_ formation in rearrangement and fragmentation reaction [64,65,66].

**Scheme 8:**
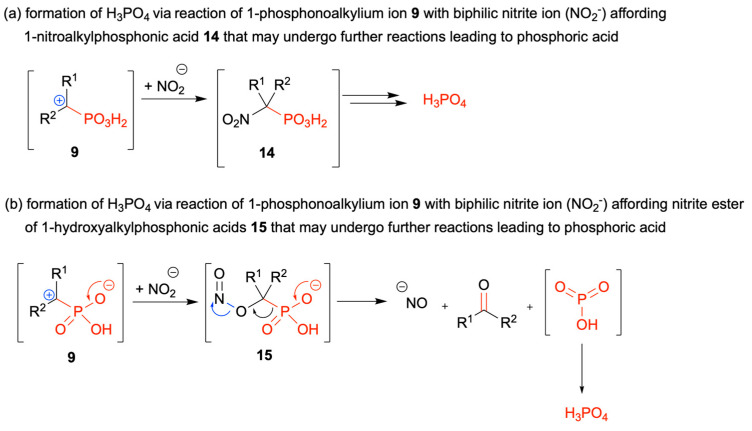
Proposed mechanisms of H_3_PO_4_ formation in the reactions of 1-phosphonoalkylium ion with nitrite ion.

Formation of 2-hydroxy-1-phenylphosphonic acid (**5″g**), as well as unrearranged **5g** and phosphoric acid may be explained by the formation of cyclic intermediate **9″g** (Scheme 6a). The nucleophilic attack of water on the less crowded side (pink arrow on Scheme 6a) of intermediate **9″g** yields rearranged 2-hydroxyalkylphosphonic acid **5″g**, while fragmentation of **9″g** (Scheme 6b) yields styrene and metaphosphoric acid which hydrolyses to phosphoric acid. Finally, when examining the reactivity of 1-aminoalkylphosphonic acids **1** in a deamination reaction with HNO_2_, in every reaction we have always observed the presence of various amounts of phosphoric acid (H_3_PO_4_). We postulate that the formation of H_3_PO_4_ could be explained by two reaction mechanisms which depend on the structure of the used 1-aminoalkylphosphonic acids **1** (Scheme 7 and Scheme 8). According to the first reaction mechanism (Scheme 7a), if the structure of the formed 1-phosphonoalkylium ion **9** enables its rearrangement to the more stable 2-phosphonoalkylium ion **9′** (compounds **1j, 1b, 1k, 1g**), then ion **9′** can further undergo fragmentation with cleavage of the C–P bond resulting in the formation of alkene and metaphosphoric acid (that undergo hydrolysis to phosphoric acid in the presence of water). A similar mechanism was proposed by Mastalerz and Richtarski for the deamination of 2-aminoethylphosphonic acid and related compounds, where the main reaction products were ethylene and phosphoric acid (Scheme 7b) [64,65,66].

The second reaction mechanism should explain the formation of H_3_PO_4_ in the case where there is no possibility of rearrangement of the formed carbenium ion **9** (Scheme 8), especially for the reaction of substrates **1a**, **1f**, **1n**, and **1h**. By analogy to reactions of 2-aminoalkanoic acids with HNO_2_ [67], the reaction of 1-phosphonoalkylium ion **9** with biphilic nitrite ion (NO_2_^-^) gives 1-nitroalkylphosphonic acid **14** (Scheme 8a) or nitrite ester of 1-hydroxyalkylphosphonic acids **15** (Scheme 8b). Compounds **14** and **15** may undergo secondary reactions which ultimately produce phosphoric acid.

## 3. Materials and Methods

### 3.1. General Information

The ^1^H, ^13^C{^1^H}, ^31^P NMR, and DEPT-135 spectra were collected on a Jeol 400yh instrument (Jeol, Ltd., Tokio, Japan) (400 MHz for ^1^H NMR, 162 MHz for ^31^P NMR, and 100 MHz for ^13^C NMR) and were processed with dedicated software (Delta 5.0.5). NMR experiments recorded in D_2_O were referenced to the respective residual ^1^H signal of the solvent. Multiplicities were reported using the following abbreviations: s (singlet), d (doublet), t (triplet), q (quartet), and m (multiplet). The reported coupling constants (*J*) values were those observed from the splitting patterns in the spectrum and may not reflect the true coupling constant values. The composition of post-reaction mixtures (as the conversion of substrate to the given product) was calculated based on ^31^P NMR (recorded in D_2_O) of the crude reaction mixture. Structural assignments of **5″g** were made with additional information from gCOSY, gHSQC, and gHMBC experiments.

### 3.2. Reagents

Aminomethylphosphonic acid (**1h**) was obtained in the reaction of benzamide, formaldehyde, and phosphorous trichloride [68]. 3-Amino-3-phosphonopropanoic acid (**1e**) was synthesized from diethyl acetamidomethylenemalonate [69]. The remaining 1-aminoalkylphosphonic acids **1** were obtained in the reaction of an appropriate carbonyl compound with acetamide, acetyl chloride, and PCl_3_ in acetic acid, using Soroka’s protocol [70]. 1-Hydroxyalkylphosphonic acids **5**, which were used as reference materials for confirmation of reaction products structures, were synthesized by dealkylation of diethyl 1-hydroxyalkylphosphonates, which were obtained in the reaction of triethyl phosphite with suitable aldehyde or ketone and hydrogen chloride [71].

### 3.3. Deamination of 1-Aminoalkylphosphonic Acids ***1*** and 2-Aminoalkanoic Acids **2** in 5M HCl

The deamination experiments were conducted in a three-necked flask equipped with a reflux condenser, thermometer, dropping funnel, and magnetic stirrer, as described in the original protocol [40]. The solution of 1-aminoalkylphosphonic acid **1** or 2-aminoalkanoic acid **2** (10 mmol) in 5M HCl (65 mmol, 13 mL) was cooled in an ice/NaCl cooling bath to a temperature of −12 °C. Subsequently, 4 M NaNO_2_ solution in water (16 mmol, 4.0 mL) was added dropwise for 2 min. The temperature of the reaction mixture was maintained under 0 °C for 5 h, and then at 25 °C for 12 h. The samples for ^1^H and ^31^P NMR spectra were prepared by diluting post-reaction mixtures (0.10 mL) in D_2_O (0.40 mL). The samples were re-measured after the addition of reference materials. The composition of the mixture was calculated based on the integration of signals on the ^31^P NMR spectra (for phosphorous substrates) or on the ^1^H NMR spectra (for 2-aminoalkanoic acids).

### 3.4. Deamination of 1-Aminoalkylphosphonic Acids ***1*** in Water

The deamination reactions of 1-aminoalkylphosphonic acids **1** were conducted in a round-bottom flask equipped with a magnetic stirrer and calibrated gas burette (Figure S11 in Supplementary Materials). The flask was placed in a water bath at a temperature of about 20 °C. 1-Aminoalkylphosphonic acid **1** (3.0 mmol) was added to a 0.67 M solution of NaNO_2_ (6.0 mmol, 9.0 mL). The solution or suspension was stirred by the means of a magnetic stirrer until the stoichiometric volume of gas was evolved, and additionally for 12 h. The ^1^H and ^31^P NMR spectra were recorded after that time and additionally after a few days. The composition of the mixture was calculated based on the integration of signals on the ^31^P NMR spectra.

## 4. Conclusions

**Scheme 9:**
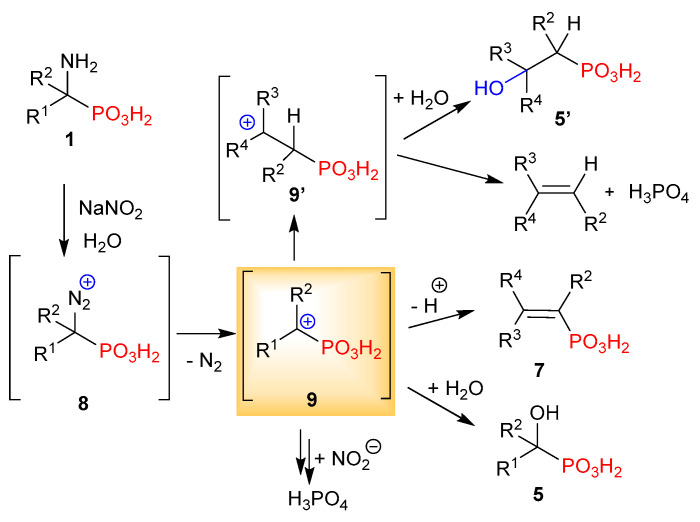
Possible transformations of 1-phosphonoalkylium ions **9** discussed in this study.

We have studied the deamination of 17 1-aminoalkylphosphonic acids **1** in the reaction with nitrous acid. We have postulated that 1-phosphonoalkylium ions **9** are plausible reactive intermediates in these reactions. Depending on the structure of 1-aminoalkylphosphonic acid **1** used, these ions **9** react with a nucleophile (H_2_O or NO_2_^¯^), undergo elimination of protons, or a rearrangement/fragmentation reaction (Scheme 9). Furthermore, we explained the formation of the phosphoric acid (H_3_PO_4_), present in every reaction mixture, through two mechanisms (Scheme 6 and Scheme 7). We have experimentally demonstrated that the selectivity of the reaction of 1-phosphonoalkylium ions **9** is not easy to control but, in some cases, the addition of nucleophile (H_2_O) is the major reaction and the starting 1-aminoalkylphosphonic acids **1** could be transformed into 1-hydroxyphosphonic acids **5** (Scheme 9). In turn, the derivatives of vinylphosphonic acid **7** resulting from proton elimination from 1-phosphonoalkylium ions **9** (Scheme 9) could be major products in the case of 1-aminoalkylphosphonic acids having a positive charge positioned at the tertiary carbon atom and surrounded by bulky substituents, such as compounds **1q, 1l,** and **1i** (Scheme 9, Table 3). Finally, if the generated 1-phosphonoalkylium ions **9** have migrating groups in the β-position, such as in compounds **9j, 9b, 9k,** and **9g**, they can further rearrange to more stable 2-phosphonoalkylium ions **9′** and either react with a nucleophile to form 2-hydroxyalkylphosphonic acid **5′** or undergo fragmentation to alkene and H_3_PO_4_ (Scheme 9, Table 4). Although the reported procedure of the deamination of 1-aminoalkylphosphonic **1** generally may have limited synthetic application, in specific cases, it may be an irreplaceable synthetic method leading to the desired products.

## Figures and Tables

**Table 1:** Reaction of HNO_2_ with 1-aminoalkylphosphonic acids **1** that are stabilized by substituents in 1-position or those that cannot rearrange ^a^.

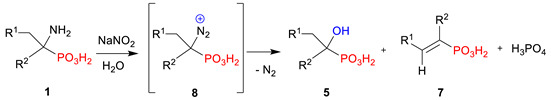						
Entry	Substrate	R1	R1	Conversion of 1 to 5 ^b^	Conversion of 1 to 7 ^b^	Conversion of 1 to H3PO4 ^b^
1	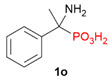	H	Ph	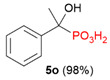	-	2%
2	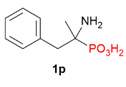	Ph	Me	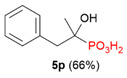	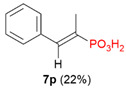	3%
3	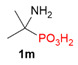	H	Me	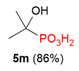	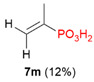	2%
4	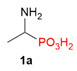	H	H	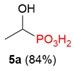	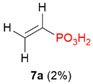	10%
5	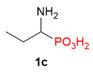	Me	H	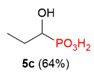	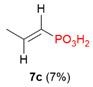	26%
6	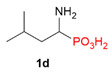	*i*Pr	H	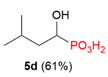	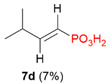	27%
7	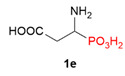	COOH	H	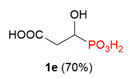	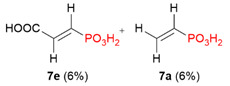	4%

^a^ Reaction conditions: 1-aminoalkylphosphonic acid (1.0 mmol), NaNO_2_ (2.0 mmol), evolution of N_2_ occurs, 21 °C, and NMR analysis of crude reaction mixture; ^b^ Conversions calculated based on ^31^P NMR (recorded in D_2_O) of the crude reaction mixture.

**Table 2:** Reaction of HNO_2_ with 1-aminoalkylphosphonic acids **1** that do not have protons in the β-position ^a^.

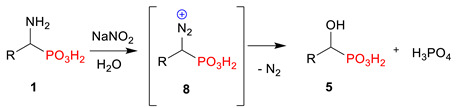				
Entry	Substrate	R	Conversion of 1 to 5 ^b^	Conversion of 1 to H_3_PO_4_^b^
1	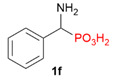	Ph	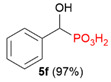	3%
2	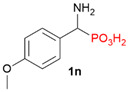	4-MeOPh	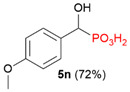	25%
3	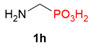	H	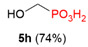	9%

^a^ Reaction conditions: 1-aminoalkylphosphonic acid (1.0 mmol), NaNO_2_ (2.0 mmol), evolution of N_2_ occurs, 21 °C, and NMR analysis of crude reaction mixture; ^b^ Conversions calculated based on ^31^P NMR (recorded in D_2_O) of the crude reaction mixture.

**Table 3:** Reaction of HNO_2_ with 1-aminoalkylphosphonic acids **1** having sterically hindered tertiary carbon atom ^a^.

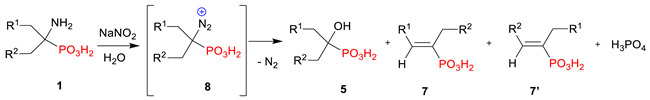							
Entry	Substrate	R^1^	R^1^	Conversion of 1 to 5 ^b^	Conversion of 1 to 7 ^b^		Conversion of 1 to H_3_PO_4_^b^
1	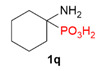	C_3_H_6_		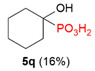			7%
2	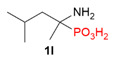	*i*Pr	H	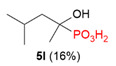	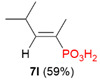	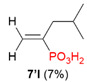	7%
3	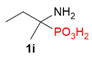	Me	H	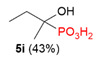	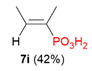	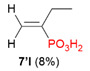	8%

^a^ Reaction conditions: 1-aminoalkylphosphonic acid (1.0 mmol), NaNO_2_ (2.0 mmol), evolution of N_2_ occurs, 21 °C, and NMR analysis of crude reaction mixture; ^b^ Conversions calculated based on ^31^P NMR (recorded in D_2_O) of the crude reaction mixture.

**Table 4:** Reaction of HNO_2_ with 1-aminoalkylphosphonic acids **1** that have a migrating group in the β-position ^a^.

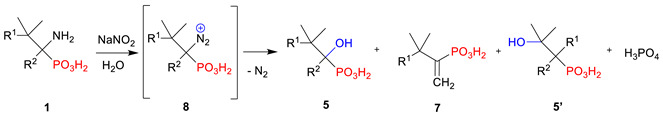							
Entry	Substrate	R^1^	R^1^	Conversion of 1 to 5 ^b^	Conversion of 1 to 7 ^b^	Conversion of 1 to 5′ ^b^	Conversion of 1 to H_3_PO_4_^b^
1	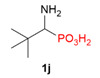	Me	H	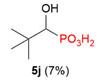	-	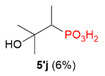	86%
2	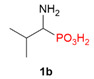	H	H	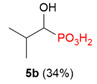	-	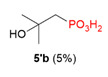	59%
3	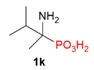	H	Me	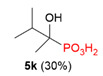	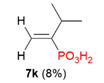	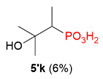	40%

^a^ Reaction conditions: 1-aminoalkylphosphonic acid (1.0 mmol), NaNO_2_ (2.0 mmol), evolution of N_2_ occurs, 21 °C, and NMR analysis of crude reaction mixture; ^b^ Conversions calculated based on ^31^P NMR (recorded in D_2_O) of the crude reaction mixture.

## Data Availability

Not applicable.

## Supplementary Materials

The following supporting information can be downloaded at: https://www.mdpi.com/article/10.3390/molecules27248849/s1, The material includes detailed procedures and NMR spectra for all reactions and compounds [72,73,74,75,76,77,78,79,80,81,82,83,84,85,86,87,88].
