# Supplementary material for: Deamination of 1-Aminoalkylphosphonic Acids: Reaction Intermediates and Selectivity
Source: Molecules. 2022 Dec 13;27(24):8849. doi: 10.3390/molecules27248849 (PMC9783495; doi:10.3390/molecules27248849)
Supplement: Supplementary file 1 [file molecules-27-08849-s001.zip › molecules-2075272- supplementary.pdf]

molecules

MDPI

Supplementary Materials

Deamination of 1-Aminoalkylphosphonic Acids: Reaction Intermediates and Selectivity

Anna Brol and Tomasz K. Olszewski *

Department of Physical and Quantum Chemistry, Faculty of Chemistry, Wrocław University of Science and Technology, ul. Wybrzeże Wyspiańskiego 27, 50-370 Wrocław, Poland.
* Correspondence: tomasz.olszewski@pwr.edu.pl

Table of contents

1. General informations. **2**
2. Deamination of 1-aminoalkylphosphonic acids and 2-aminoalkanoic acids in 5M HCl ... **2**
ABr738. Deamination of 1a in 5M hydrochloric acid. 3
ABr742. Deamination of 1b in 5M hydrochloric acid 7
ABr746. Deamination of 2a in 5M hydrochloric acid. 11
ABr748. Deamination of 2b in 5M hydrochloric acid 12
3. Deamination of 1-aminoalkylphosphonic acids in water. **14**
ABr1178. Deamination of 1a in water. 15
ABr1204. Deamination of 1m water. 18
ABr1206. Deamination of 1o in water. 22
ABr1208. Deamination of 1b in water. 25
ABr1210. Deamination of 1d in water. 29
ABr1212. Deamination of 1c in water. 32
ABr1214. Deamination of 1i in water. 35
ABr1216. Deamination of 1l in water. 39
ABr1218. Deamination of 1k in water. 43
ABr1220. Deamination of 1j in water. 46
ABr1222. Deamination of 1q in water. 49
ABr1224. Deamination of 1f in water. 52
ABr1226. Deamination of 1n in water. 55
ABr1228. Deamination of 1h in water. 60
ABr1234. Deamination of 1p in water. 62
ABr1236. Deamination of 1g in water. 65
ABr1248. Deamination of 1e in water. 70
4. References. **74**

1. General Informations

The ^1H, $^{13}C\{^1H\}$, ^{31}P NMR and DEPT-135 spectra were collected on a Jeol 400yh instrument (400 MHz for ^1H NMR, 162 MHz for ^{31}P NMR and 100 MHz for $^{13}C\{^1H\}$ NMR) and were processed with dedicated software (Delta 5.0.5). NMR experiments recorded in D_2O were referenced to the respective residual ^1H signal of the solvent. Multiplicities were reported using the following abbreviations: s (singlet), d (doublet), t (triplet), q (quartet), and m (multiplet). The reported coupling constants (*J*) values were those observed from the splitting patterns in the spectrum and may not reflect the true coupling constant values. The composition of post-reaction mixtures (as conversion of substrate to the given product) were calculated based on ^{31}P NMR (recorded in D_2O) of the crude reaction mixture. Structural assignments of **5′′g** were made with additional information from gCOSY, gHSQC, and gHMBC experiments.

Reagents. Aminomethylphosphonic acid **(1h)** was obtained in the reaction of benzamide, fomaldehyde and phosphorous trichloride [1]. 3-Amino-3-phosphonopropanoic acid **(1e)** was synthesized from diethyl acetamidomethylenemalonate [2]. Remaining 1-aminoalkylphosphonic acids **1** were obtained using Sorokas' protocol and the reaction of an appropriate carbonyl compound with acetamide, acetyl chloride and PCl_3 in acetic acid [3].

1-Hydroxyalkylphosphonic acids **5**, which were used as reference materials for confirmation of reaction products structures, were synthesized by dealkylation of diethyl 1-hydroxyalkylphosphonates. The substrates were obtained in the reaction of triethyl phosphite with suitable aldehyde or ketone and hydrogen chloride [4].

2. Deamination of 1-Aminoalkylphosphonic Acids and 2-Aminoalkanoic Acids in 5M HCl

Preliminary deamination experiments were contucted in three-necked flask equipped with reflux condenser, thermometer, dropping funnel and magnetic stirrer, as decsribed in the original protocol [5].

General procedure. Preliminary deamination experiments were contucted in three-necked flask equipped witreflux condenser, thermometer, dropping funnel and magnetic stirrer, as decsribed in the original protocol [5]. The solution of 1-aminoalkylphosphonic acid **1** or 2-aminoalkanoic acid **2** (10 mmol, 1 equiv) in 5M HCl (6.5 equiv, 13mL) was cooled in ice/NaCl cooling bath to temperature 0 °C. Cooled 4M $NaNO_2$ solution in water (16 mmol, 1.6 equiv, 4.0 mL) was subsequently added dropwise for 2 min. Temperature of the reaction mixture was maintained under 0 °C for 5h, and then in 25 °C for 12 h. The samples for ^1H and ^{31}P NMR spectra were prepared by diluting post-reaction mixtures (0.10mL) in D_2O (0.40mL). The samples were re-measured after addition of reference materials. The composition of mixture was calculated based on the integration of signals on the ^{31}P NMR spectra (for phosphorous substrates) or on the ^1H NMR spectra (for 2-aminoalkanoic acids).

*2.1. ABr738. Deamination of **1a** in 5M Hydrochloric Acid*

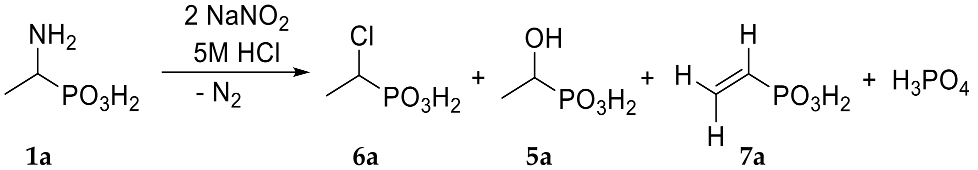

1a **6a** **5a** **7a**

	α = 0.99	53%	24%	21%	1%
δ_P [ppm]	16.88 (dq)	20.56 (dq)	25.57(dq)	17.16 (ddd)	0.39 (s)

After 12h in 21 °C, post-reaction mixture contained 1-chloroethylphosphonic acid **(6a)** (53%molP), 1-hydroxyethylphosphonic acid **(5a)** (24%molP), vinylphosphonic acid **(7a)** (21%molP), orthophosphoric acid (1%molP) and unreacted substrate **1a** (1%molP) (Figure S1 and Figure S2).

1-Hydroxyethylphosphonic acid **(7a)** was identified on ^{1}H and ^{31}P NMR spectra by re-measuring the NMR spectra of the reaction mixture with the addition of post-reaction mixture of 1-aminoethylphosphonic acid with $NaNO_2$ in water (ABr1178), where this compound **5a** was the main product (Figure S3). Vinylphosphonic acid **(7a)** and 1-chlorophosphonic acid **(6a)** were identified by comparing chemical shifts, multiplecity and coupling constants on ^{1}H and ^{31}P NMR spectra with description in the literature (Table S1) [6, 7].

The presence of 1-chloroethylphosphonic acid **(6a)** was additionally confirmed by careful analysis of NMR spectra. In the post-reaction mixture there were two compounds having the structure $-CH(CH_3)PO_3H_2$ (δ_P = 20.56 ppm and δ_P = 25.57 ppm). 1-Hydroxyethylphosphonic acid **(5a)** (δ_P = 25.57 ppm) was identified earlier. The presence of the unreacted substrate was excluded by re-reasuring NMR spectra of post-reaction mixture with addition of 1-aminoethylphosphonic acid **(1a)** (Figure S4), which resulted in appearance of new peak (δ_P = 16.88 ppm). Therefore, the second product was 1-chloroethylphosphonic acid **(6a)** (δ_P = 20.56 ppm).

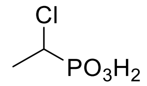

1-Chloroethylphosphonic acid (6a). ^{31}P NMR (D_2O, ca. 0.8M HCl): δ 20.56 (dq, $^2J_{H\text{-}P}$ = 9.4 Hz, $^3J_{H\text{-}P}$ = 15.9 Hz); ^{1}H NMR (D_2O, ca. 0.8M HCl) δ: 3.85 (dq, 1H, $^3J_{H\text{-}H}$ = 7.3 Hz, $^2J_{H\text{-}P}$ = 9.00 Hz), 1.36 (dd, 3H, $^3J_{H\text{-}H}$ = 7.3 Hz, $^3J_{H\text{-}P}$ = 16.5 Hz).

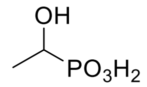

1-Hydroxyethylphosphonic acid (5a). ^{31}P NMR (D_2O, ca. 0.8M HCl): δ 25.57 (dq, $^2J_{H\text{-}P}$ = 5.6 Hz, $^3J_{H\text{-}P}$ = 17.6 Hz); ^{1}H NMR (D_2O, ca. 0.8M HCl) δ: 3.77 (dq, 1H, $^2J_{H\text{-}P}$ = 5.2 Hz, $^3J_{H\text{-}H}$ = 7.0 Hz), 1.16 (dd, 3H, $^3J_{H\text{-}H}$ = 7.0 Hz, $^3J_{H\text{-}P}$ = 17.4 Hz).

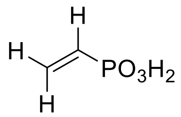

Vinylphosphonic acid (7a). ^{31}P NMR (D_2O, ca. 0.8M HCl) δ: 17.16 (ddd, $^2J_{H\text{-}P}$ = 24.3 Hz, $^3J_{H\text{-}P\ (cis)}$ = 26.9 Hz, $^3J_{H\text{-}P\ (trans)}$ = 49.3 Hz); ^1H NMR (D_2O, ca. 0.8M HCl) δ: 5.75-6.03 (m, 3H).

Table S1. Comparison of chemical shifts and copuling constants of 1-aminoethylphosphonic acid **(1a)** deamination products in 5M HCl on ^1H and ^{31}P NMR spectra with literature description.

**Structure**	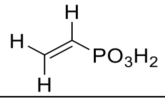		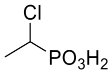	
**Source**	**ABr738**	**Reference [6]**	**ABr738**	**Reference [7]**
**Solvent**	**D_2O, ca. 0.8M HCl**	**$CDCl_3$**	**D_2O, ca. 0.8M HCl**	**"hydrolyzate"**
**^{31}P NMR**	17.16 (ddd, *J* = 24.3, 26.9, 49.3 Hz)	17.3 (ddd, *J* = 20.0, 20.5, 51.3 Hz)	20.56 (dq, *J* = 9.4, 15.9 Hz)	20.1
**^1H NMR**	5.75-6.03 (m, 3H)	6.31 (ddd, 1H, *J* = 20.5, 16.2, 4.5 Hz)	3.85 (dq, 1H, *J* = 7.3, 9.00 Hz)	
		6.05 (ddd, 1H, *J* = 51.3, 11.2, 4.5 Hz),	1.36 (dd, 3H, *J* = 7.3, 16.5 Hz)	
		6.02 (ddd, 1H, *J* = 20.0, 16.2, 11.2 Hz)		

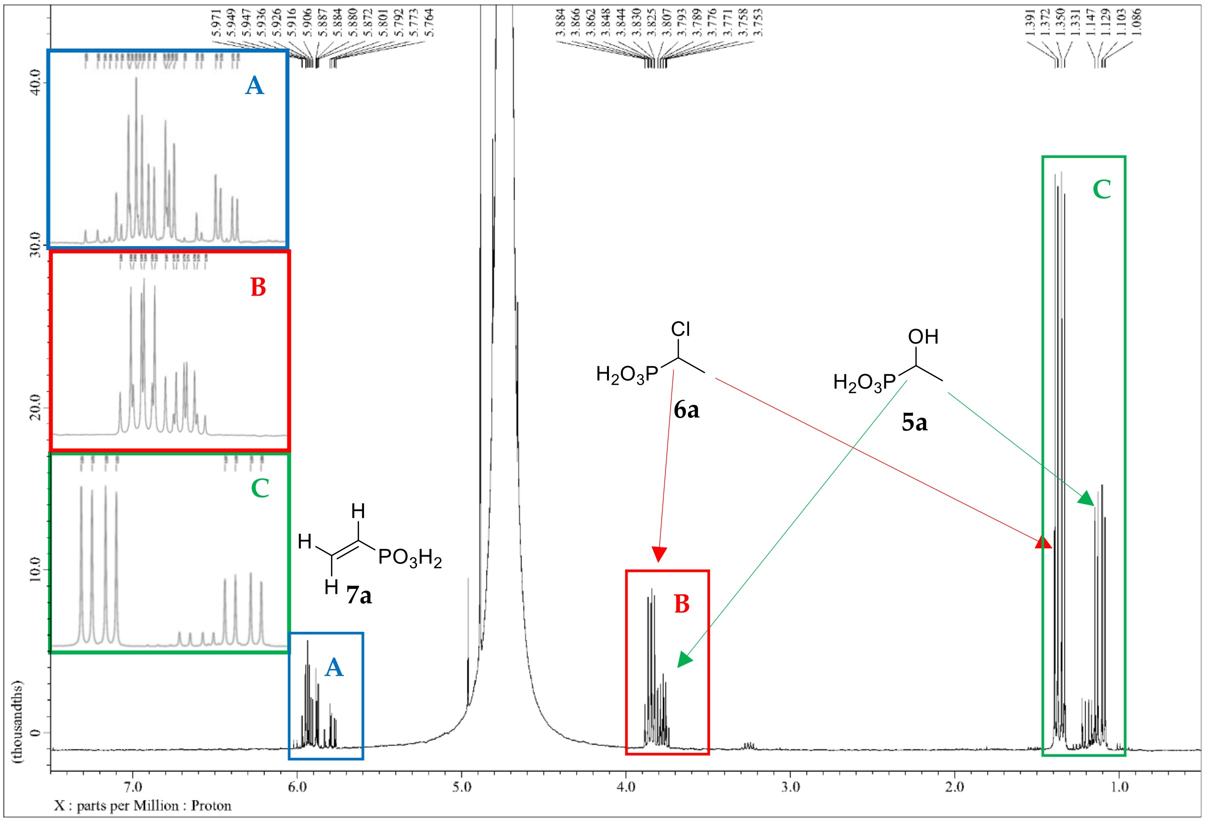

Figure S1. ^{1}H NMR (D_2O, 400MHz) spectra of post-reaction mixture of 1-aminoethylphosphonic acid **(1a)** with $NaNO_2$ in 5M hydrochloric acid.

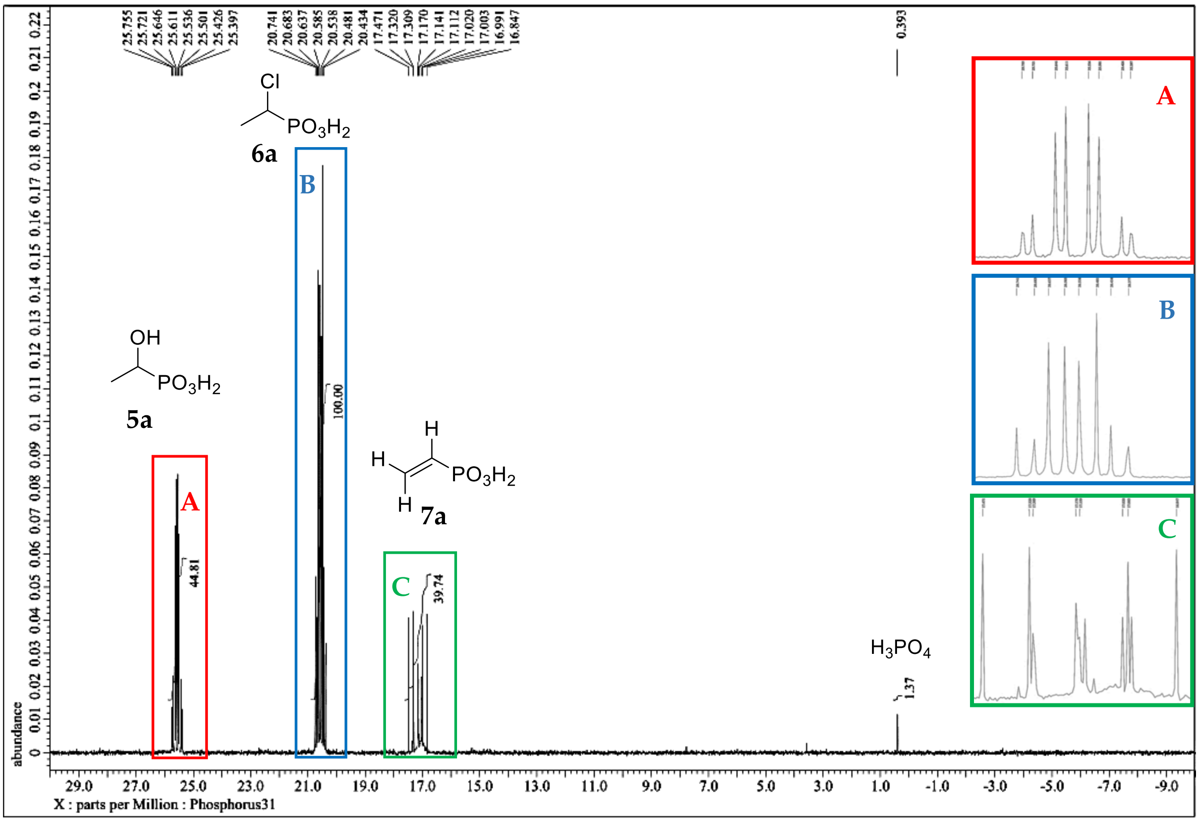

Figure S2. ^{31}P NMR (D_2O, 162MHz) spectra of post-reaction mixture of 1-aminoethylphosphonic acid **(1a)** with $NaNO_2$ in 5M hydrochloric acid.

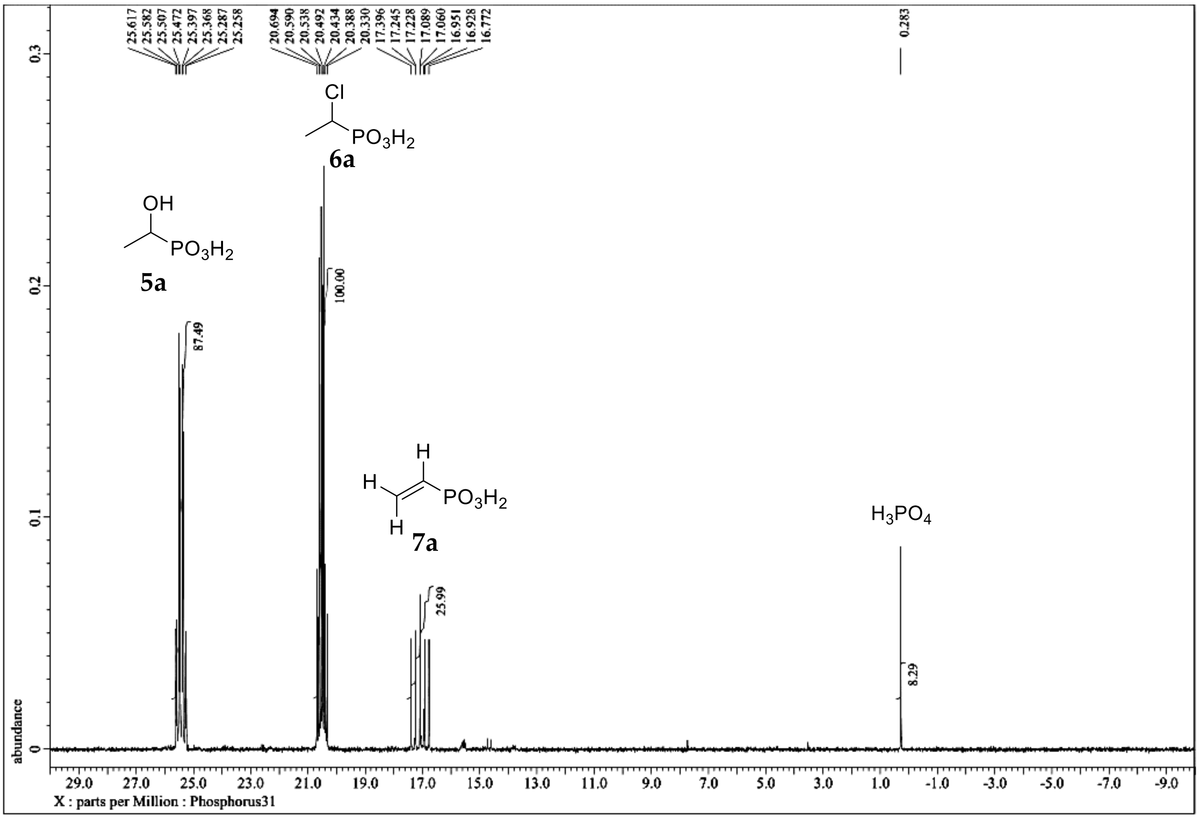

Figure S3. ^{31}P NMR (D_2O, 162MHz) spectra of post-reaction mixture of 1-aminoethylphosphonic acid **(1a)** with $NaNO_2$ in 5M hydrochloric acid with addition of 1-hydroxyethylphosphonic acid **(5a)** as standard.

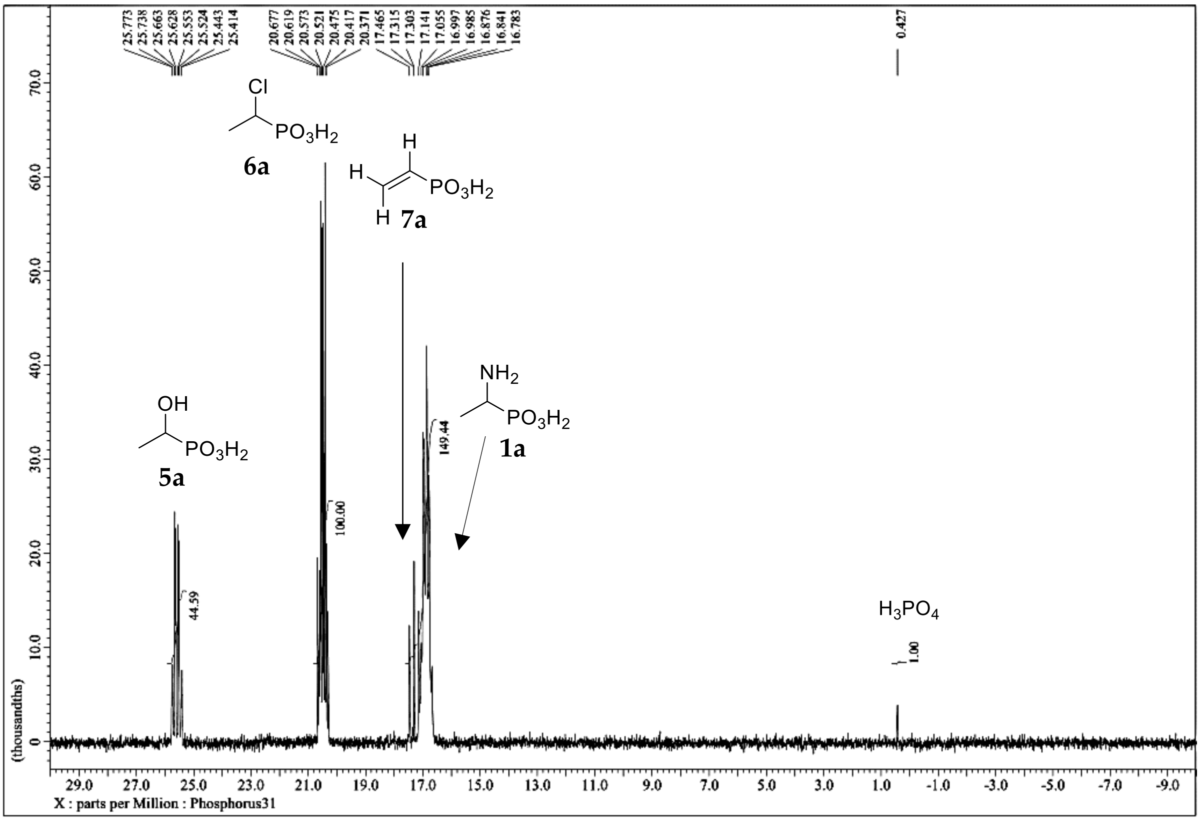

Figure S4. ^{31}P NMR (D_2O, 162MHz) spectra of post-reaction mixture of 1-aminoethylphosphonic acid **(1a)** with $NaNO_2$ in 5M hydrochloric acid with addition of substrate **(1a)** as standard.

*2.2. ABr742. Deamination of **1b** in 5M Hydrochloric Acid*

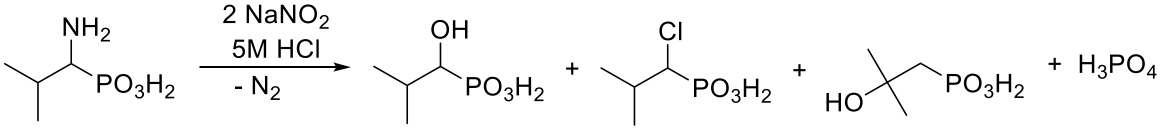

1b **5b** **6b** **5′b**

	α = 0.61	6%	2%	42%	9%
δ_P [ppm]	16.88 (dq)	25.02 (dq)	19.37 (dq)	27.00 (t)	0.39 (s)

After 12h in 21 °C, post-reaction mixture contained 1-hydroxy-2-methylpropylphosphonic acid **(5b)** (6%molP), 1-chloro-2-methylpropylphosphonic acid **(6b)** (2%molP), 2-hydroxy-2-methylpropylphosphonic acid **(5′b)** (42%molP) and orthophosphoric acid (9%molP) (Figure S5 and Figure S7).

Unreacted 1-amino-2-methylpropylphosphonic acid was identified on ^{1}H and ^{31}P NMR spectra by re-measuring the NMR spectra of the reaction mixture with the addition of substrate (Figure S6 and Figure S8).

1-Hydroxy-2-methylpropylphosphonic acid **(5b)** was identified by comparing chemical shifts, multiplecity and coupling constants on ^{1}H and ^{31}P NMR spectra with description in the literature (Table S2).

Spectra of 2-hydroxy-2-methylpropylphosphonic acid **(5′b)** and 1-chloro-2-methylpropylphosphonic acid **(6b)** have not been described in the literature. Therefore, these compounds were identified by analysis of the signals multiplecity on the ^{1}H and ^{31}P NMR spectra. Moreover, ^{1}H and ^{31}P NMR spectra of 2-hydroxy-2-methylpropylphosphonic acid **(5′b)** were compared with spectra of similar 2-hydroxypropylphosphonic acid (Table S2).

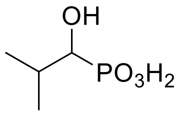

1-Hydroxy-2-methylpropylphosphonic acid (5b). ^{31}P NMR (D_2O, ca. 0.8M HCl): δ 25.02 (dd, $^{2}J_{H\text{-}P}$ = 8.4 Hz, $^{3}J_{H\text{-}P}$ = 8.4 Hz); ^{1}H NMR (D_2O, ca. 0.8M HCl): δ 3.46 (dd, 1H, $^{2}J_{H\text{-}P}$ = 8.3 Hz, $^{3}J_{H\text{-}H}$ = 5.8 Hz), 1.70-1.93 (m, 1H), 0.81 (d, 3H, $^{3}J_{H\text{-}H}$ = 6.7 Hz), 0.80 (d, 3H, $^{3}J_{H\text{-}H}$ = 6.7 Hz).

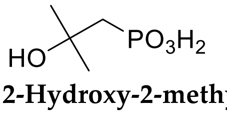

2-Hydroxy-2-methylpropylphosphonic acid (5′b). ^{31}P NMR (D_2O, ca. 0.8M HCl): δ 27.00 (t, $^{2}J_{H\text{-}P}$ = 19.2 Hz); ^{1}H NMR (D_2O, ca. 0.8M HCl) δ: 1.95 (d, 3H, $^{2}J_{H\text{-}P}$ = 18.7 Hz, 2H), 1.16 (s, 6H).

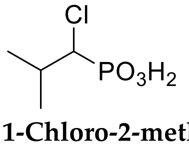

1-Chloro-2-methylpropylphosphonic acid (6b). ^{31}P NMR (D_2O, ca. 0.8M HCl): δ 19.37 (dd, $^{2}J_{H\text{-}P}$ = 10.3 Hz, $^{3}J_{H\text{-}P}$ = 7.5 Hz); ^{1}H NMR (D_2O, ca. 0.8M HCl): δ 3.83 (dd, 1H, $^{2}J_{H\text{-}P}$ = 11.3 Hz, $^{3}J_{H\text{-}H}$ = 3.7 Hz), 2.10-2.21 (m, 1H), 0.82-0.92 (6H).

Table S2. Comparison of chemical shifts and coupling constants on ^{1}H and ^{31}P NMR spectra of the reaction products of 1-amino-2-methylpropylphosphonic acid **(1b)** with $NaNO_2$ in 5M HCl with compounds described in the literature.

**Structure**	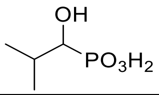		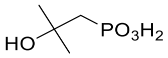	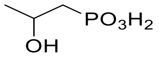
**Source**	**ABr742**	**Reference [8]**	**ABr742**	**Reference [9]**
**Solvent**	**(D_2O, 0.8M HCl)**	**D_2O**	**D_2O**	**D_2O**
**^{31}P NMR**	25.02 (t, *J* = 8.4 Hz)	19.6	21.24 (t, *J* = 17.8 Hz)	19.9
**^{1}H NMR**	3.46 (dd, 1H, *J* = 8.3, 5.8 Hz)	3.23 (dd, 1H, *J* = 8.4, 6.0 Hz)		3.80 (1H, dm, *J* = 6.5 Hz)
	1.70-1.93 (m, 1H)	1.79-1.86 (m, 1H)	1.83 (d, 3H, *J* = 17.7 Hz)	1.39 (2H, ddd, *J* = 18.0, 6.6, 15.3 Hz)
	0.81 (d, 3H, *J* = 6.7 Hz)	0.84 (d, 3H, *J* = 6.6 Hz)	1.22 (s, 6H)	0.97 (3H, d, *J* = 6.5 Hz)
	0.80 (d, 3H, *J* = 6.7 Hz)	0.83 (d, 3H, *J* = 6.6 Hz)		

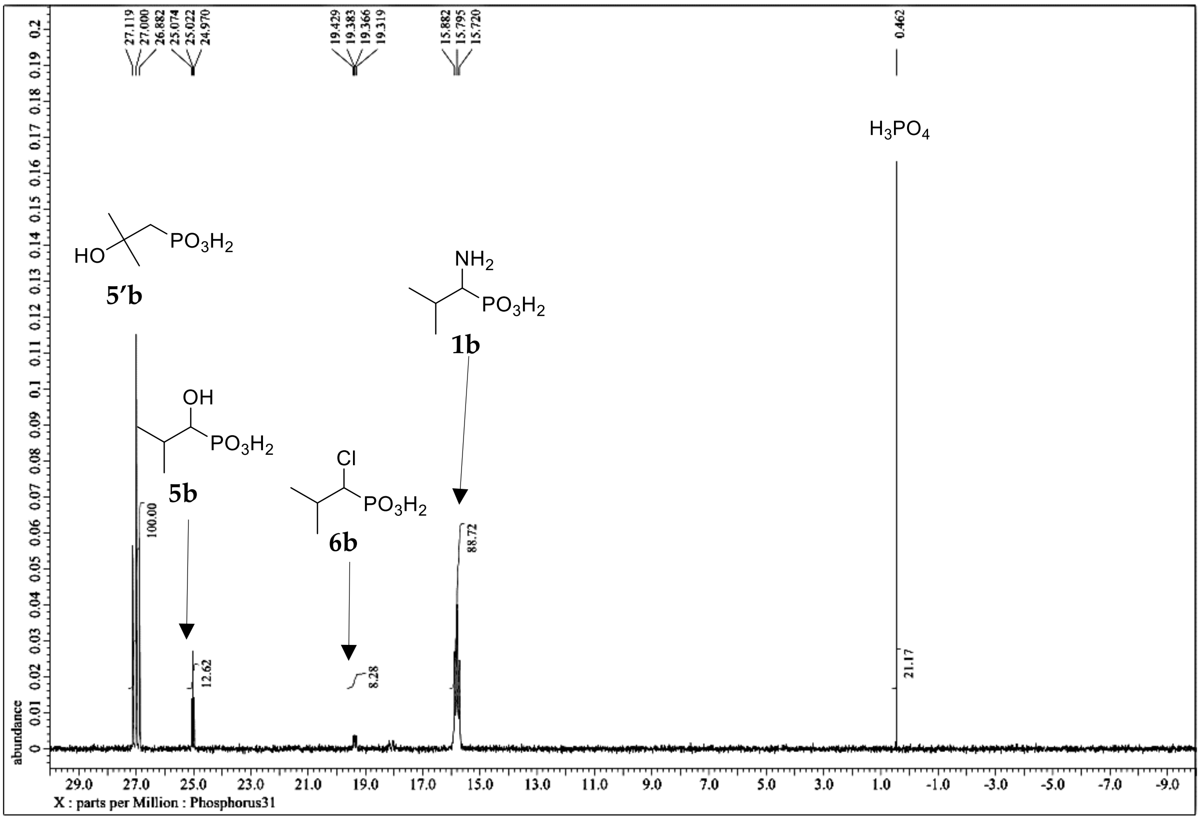

Figure S5. ^{31}P NMR (D_2O, 162MHz) spectra of the crude post-reaction mixture of 1-amino-2-methylpropylphosphonic acid **(1b)** with $NaNO_2$ in 5M HCl.

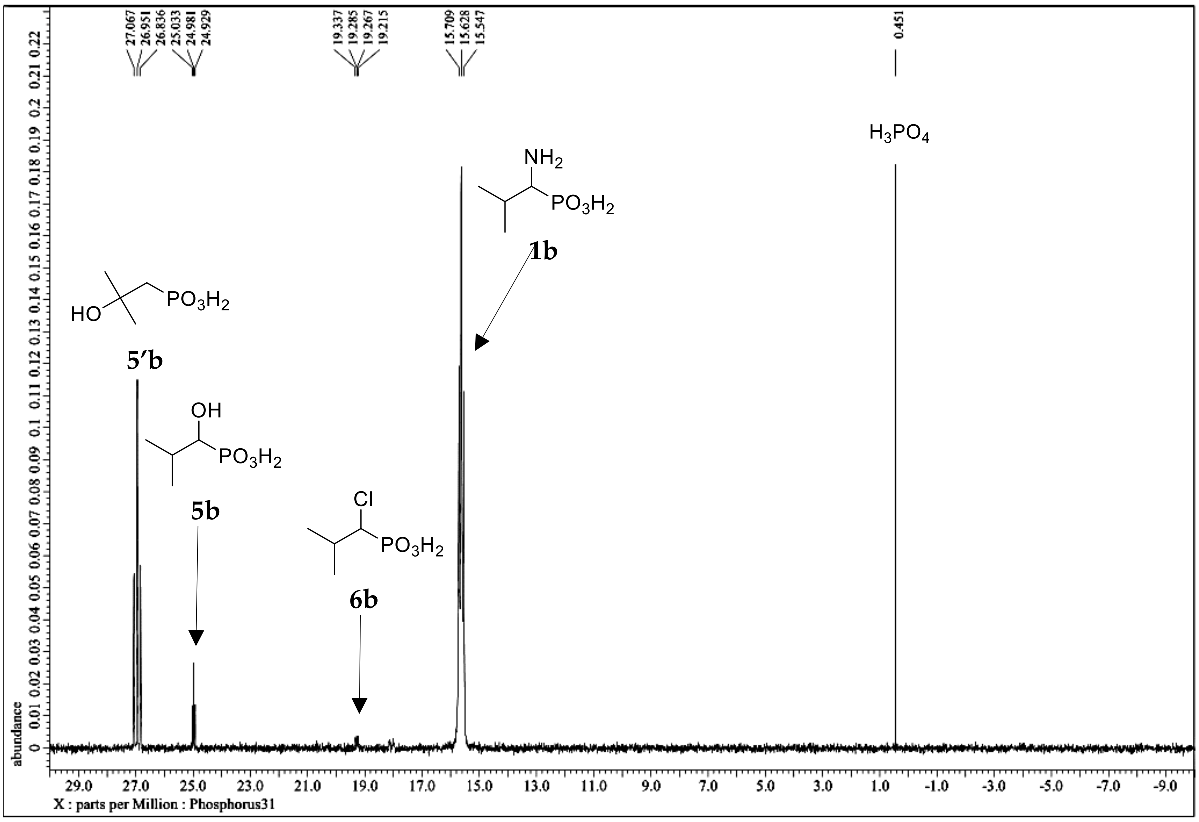

Figure S6. ^{31}P NMR (D_2O, 162MHz) spectra of the crude post-reaction mixture of 1-amino-2-methylpropylphosphonic acid **(1b)** with $NaNO_2$ in 5M HCl with addition of substrate **(1b)** as standard.

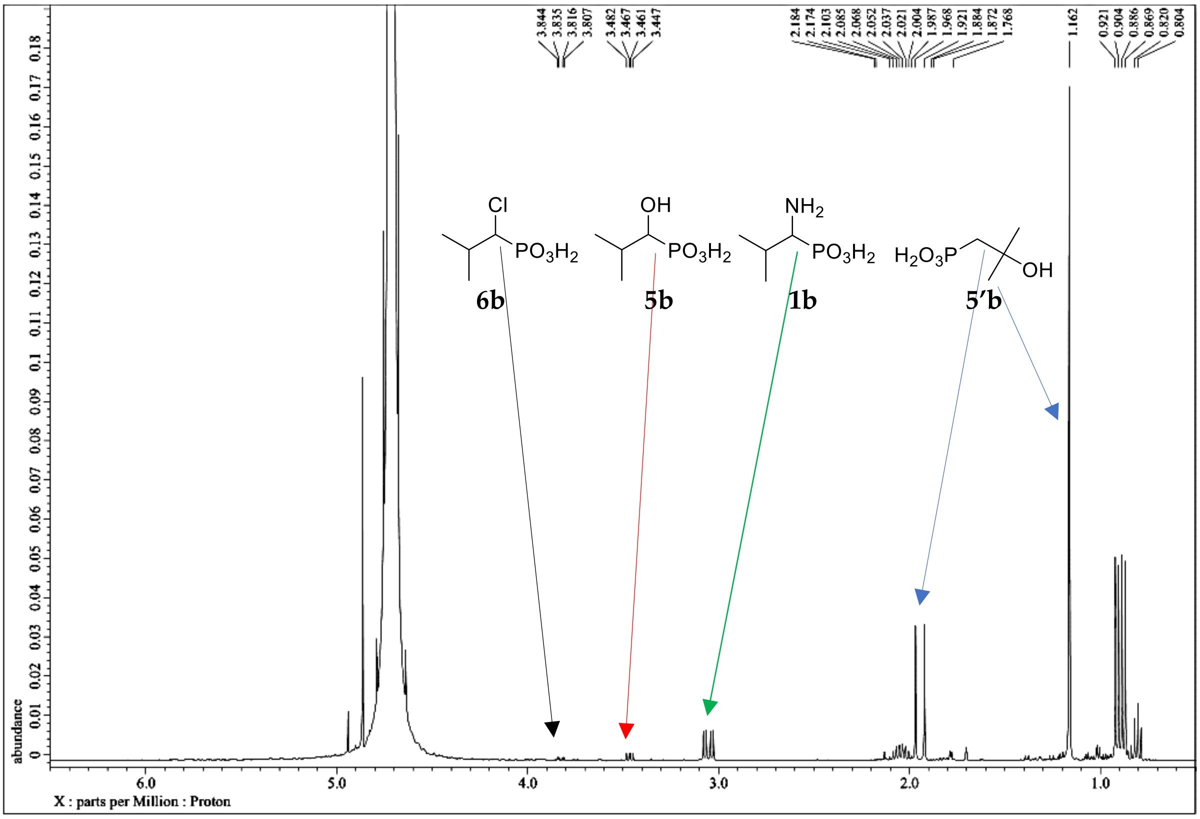

Figure S7. ^{1}H NMR (D_2O, 400MHz) spectra of the crude post-reaction mixture of 1-amino-2-methylpropylphosphonic acid **(1b)** with $NaNO_2$ in 5M HCl.

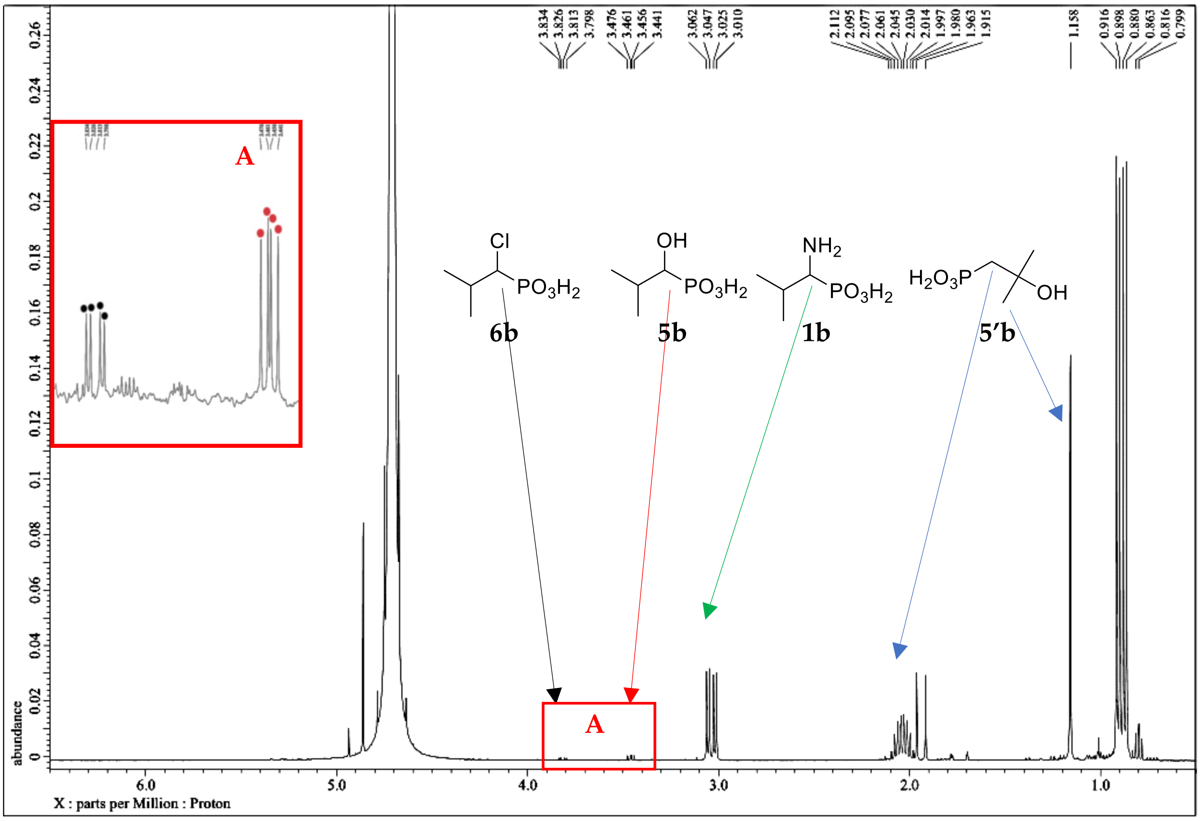

Figure S8. ^{1}H NMR (D_2O, 400MHz) spectra of the crude post-reaction mixture of 1-amino-2-methylpropylphosphonic acid **(1b)** with $NaNO_2$ in 5M HCl with addition of substrate **(1b)** as standard.

2.3. ABr746. Deamination of ***2a*** *in 5M Hydrochloric Acid*

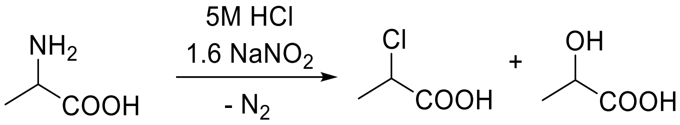

2a **3a** **4a**

α = 0.53	35%	18%

After 24 h in 21 °C, post-reaction mixture contained unreacted substrate **2a** (47%mol), 2-chloropropanoic acid **(3a)** (35%mol) and 2-hydroxypropanoic acid **(4a)** (18%mol) (Figure S9).

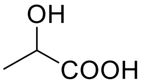

2-Hydroxypropanoic acid (4a). ^{1}H NMR (D_2O, ca. 0.8M HCl) δ: 4.12 (q, 1H, $^3J_{H\text{-}H}$ = 7.0 Hz), 1.15 (d, 3H, $^3J_{H\text{-}H}$ = 7.0 Hz).

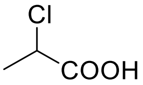

2-Chloropropanoic acid (3a). ^{1}H NMR (D_2O, ca. 0.8M HCl) δ: 4.35 (q, 1H, $^3J_{H\text{-}H}$ = 7.0 Hz), 1.41 (d, 3H, $^3J_{H\text{-}H}$ = 7.0 Hz).

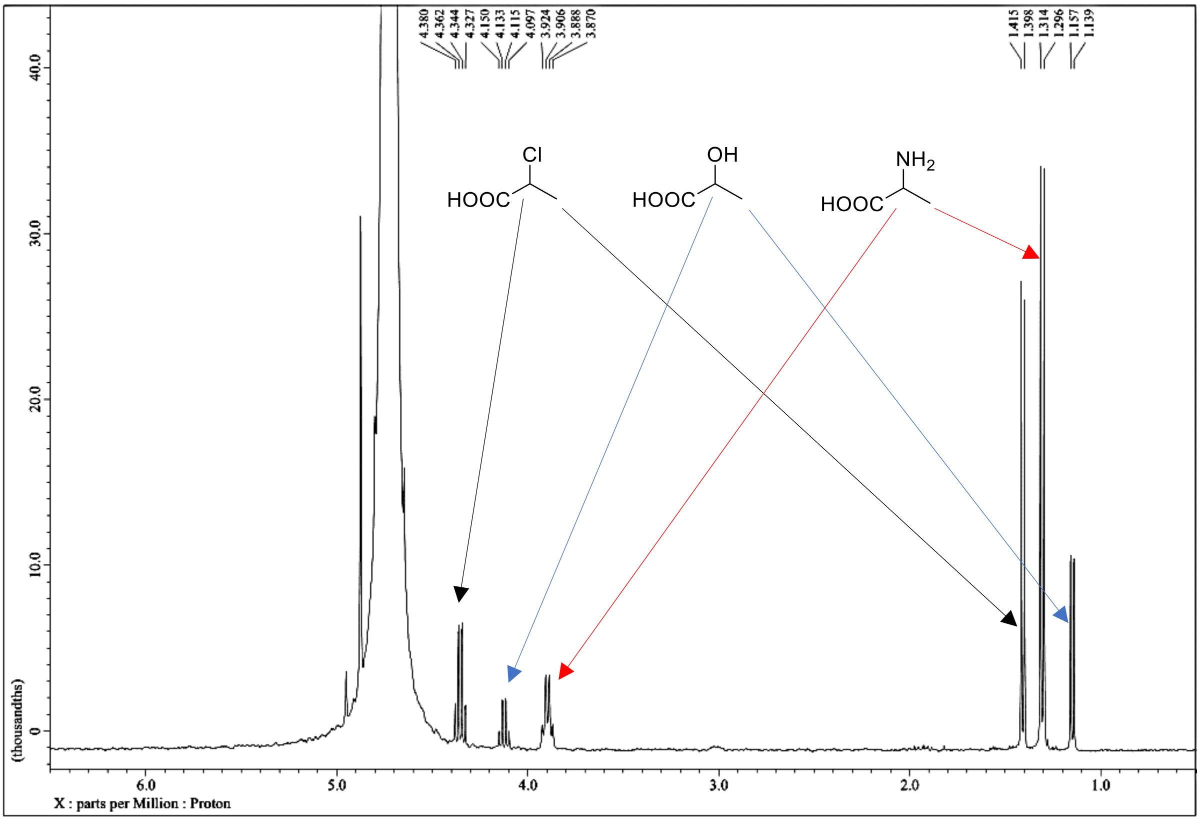

Figure S9. ^{1}H NMR (D_2O, 400MHz) spectra of crude post-reaction mixture of alanine **(2a)** with $NaNO_2$ in 5M hydrochloric acid.

*2.4. ABr748. Deamination of **2b** in 5M Hydrochloric Acid*

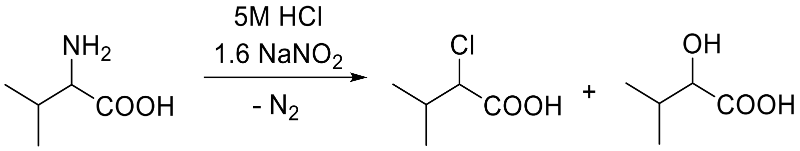

2b **3b** **4b**

$\alpha = 0.57$	40%	8%

After 24 h in 21 °C, post-reaction mixture contained unreacted substrate **2b** (43%mol), 2-chloro-3-methylbutanoic acid **(3b)** (40%mol) and 2-hydroxy-3-methylbutanoic acid **(4b)** (8%mol) and unidentified compound (Figure S10).

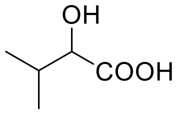

2-Hydroxy-3-methylbutanoic acid (4b). ^{1}H NMR (D_2O, ca. 0.8M HCl) δ: 3.86 (d, 1H, $^{3}J_{H\text{-}H}$ = 4.0 Hz), 1.82 (doublet of septets, 1H, $^{3}J_{H\text{-}H}$ = 7.0 Hz, $^{3}J_{H\text{-}H}$ = 4.0 Hz), 0.71 (d, 3H, $^{3}J_{H\text{-}H}$ = 7.0 Hz), 0.62 (d, 3H, $^{3}J_{H\text{-}H}$ = 7.0 Hz).

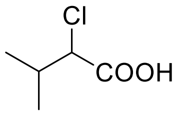

2-Chloro-3-methylbutanoic acid (3b). ^{1}H NMR (D_2O, ca. 0.8M HCl) δ: 4.17 (d, 1H, $^{3}J_{H\text{-}H}$ = 5.5 Hz), 2.05 (doublet of septets, 1H, $^{3}J_{H\text{-}H}$ = 6.7 Hz, $^{3}J_{H\text{-}H}$ = 5.8 Hz), 0.76 (d, 3H, $^{3}J_{H\text{-}H}$ = 6.7 Hz), 0.72 (d, 3H, $^{3}J_{H\text{-}H}$ = 6.7 Hz).

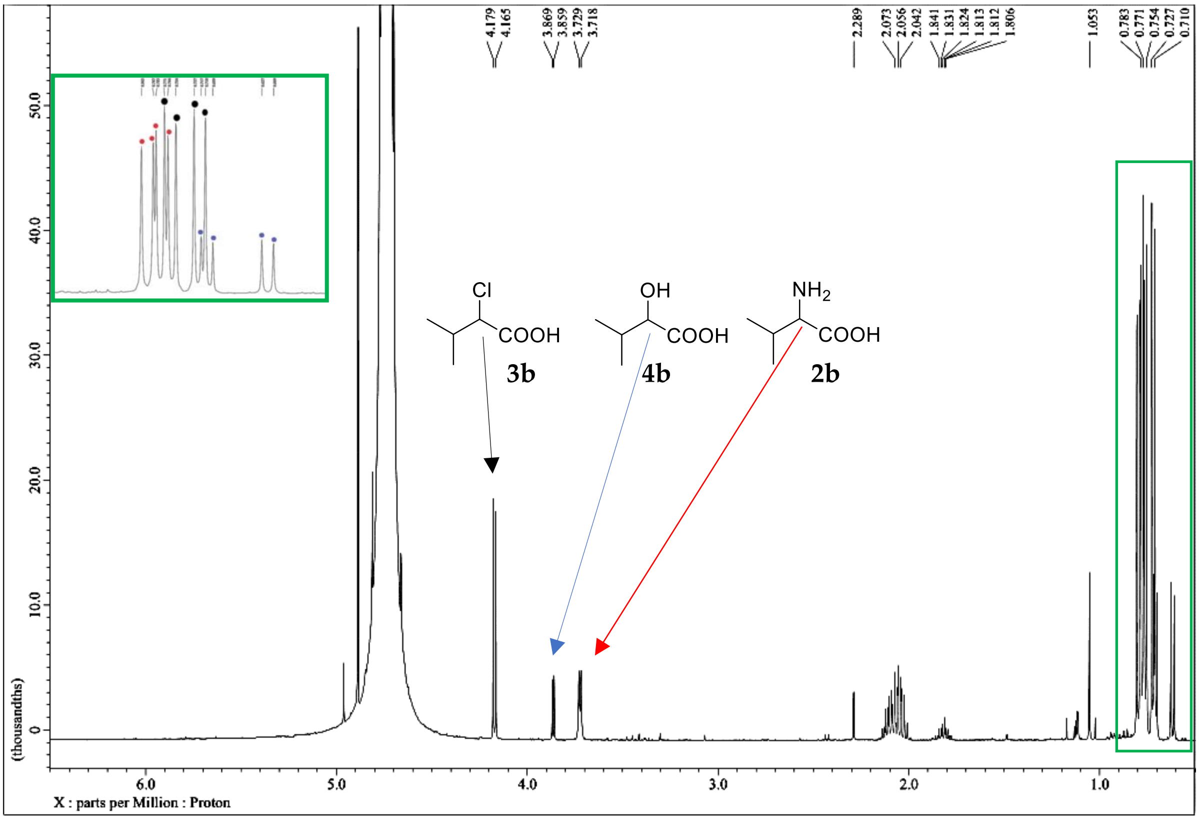

Figure S10. ^{1}H NMR (D_2O, 400MHz) spectra of crude post-reaction mixture of valine **(2b)** with $NaNO_2$ in 5M hydrochloric acid.

3. Deamination of 1-Aminoalkylphosphonic Acids in Water

Apparatus. Deamination reactions of 1-aminoalkylphosphonic acids **1** were conducted in round-bottom flask equipped with magnetic stirrer and calibrated gas burette (Figure S11). The flask was placed in a water bath at a temperature of about 20 °C. 1-Aminoalkylphosphonic acid **1** (3.0 mmol, 1 eqiv) was added to 0.67 M solution of $NaNO_2$ (6.0 mmol, 2 equiv, 9.0 mL). Solution or suspension was stirred by the means of magnetic stirrer until the stoichiometric volume of gas was evolved and additionally for 12 h. ^{1}H and ^{31}P NMR spectra were recorded after that time and additionally after few days. The composition of mixture was calculated based on the integration of signals on the ^{31}P NMR spectra.

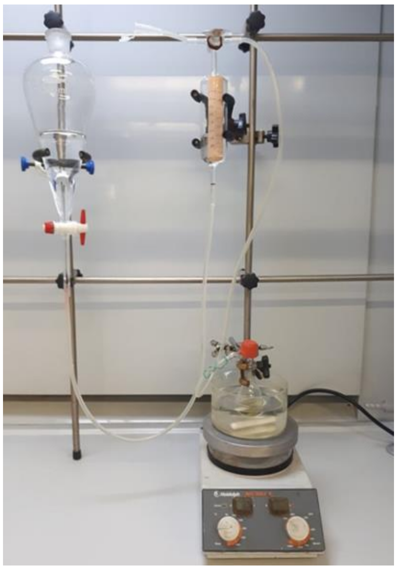

Figure S11. Apparatus used in the deamination reactions of 1-aminoalkylphosphonic acids **1** with sodium nitrite in water.

General Procedure. 1-aminoalkylphosphonic acid **1** (3.0 mmol, 1 eqiv) was added to cooled in water bath 0.67M solution of $NaNO_2$ (6.0 mmol, 2 equiv, 9.0 mL). Solution or suspension was stirred by the means of magnetic stirrer until the stoichiometric volume of gas was evolved and additionally for 12 h. ^{1}H and ^{31}P NMR spectra were recorded after that time and additionally after few days. The composition of mixture was calculated based on the integration of signals on the ^{31}P NMR spectra.

*3.1. ABr1178. Deamination of **1a** in Water*

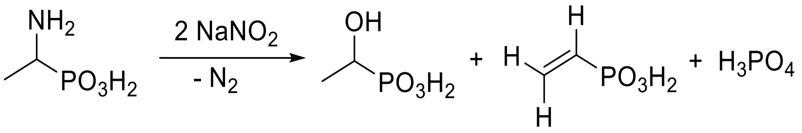

1a **5a** **7a**

24 h	$\alpha = 0.98$	85%	1%	9%
312 h	$\alpha = 1.00$	84%	2%	10%
δ_P [ppm]	13.6 (dq)	21.33 (dq)	12.9 (ddd)	1.25 (s)

After 24 h at 21 °C, post-reaction mixture contained 1-hydroxyethylphosphonic acid **(5a)** (85%molP), phosphoric acid (9%molP), vinylphosphonic acid **(7a)** (ca. 1%molP), unreacted substrate **(1a)** (2%molP) and 4 unidentified compounds (ca. 3%molP in total).

After 312 h at 21 °C, post-reaction mixture contained 1-hydroxyethylphosphonic acid **(5a)** (84%molP), orthophosphoric acid (10%molP), vinylphosphonic acid **(7a)** (ca. 2%molP) and 4 unidentified compounds (ca. 4%molP in total) (Figure S12 and Figure S14).

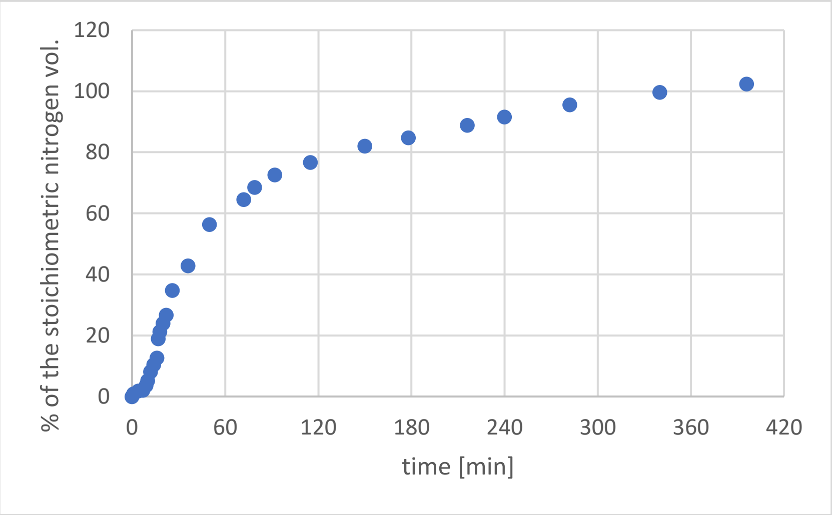

Chart 1. Dependence of the volume of the released gas (in % of the stoichiometric nitrogen volume) on the time in the reaction of 1-aminoethylphosphonic acid **(1a)** with $NaNO_2$ in water.

Vinylphosphonic acid **(7a)** and 1-hydroxyethylphosphonic acid **(5a)** identified by comparing chemical shifts, multiplecity and coupling constants on ^1H and ^{31}P NMR spectra with description in the literature (Table S3)

Additionally, 1-hydroxyethylphosphonic acid **(5a)** was identified on ^1H and ^{31}P NMR spectra by re-measuring the NMR spectra from the reaction mixture with the addition of 1-hydroxyethylphosphonic acid **(5a)** standard (Figure S13).

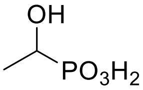

1-Hydroxyethylphosphonic acid (5a). ^{31}P NMR (D_2O): δ 21.33 (dq, $^2J_{H\text{-}P}$ = 5.6 Hz, $^3J_{H\text{-}P}$ = 15.4 Hz); ^1H NMR (D_2O) δ: 3.70 (dq, 1H, $^2J_{H\text{-}P}$ = 5.9 Hz, $^3J_{H\text{-}H}$ = 7.0 Hz), 1.19 (dd, 3H, $^3J_{H\text{-}H}$ = 7.0 Hz, $^3J_{H\text{-}P}$ = 15.9 Hz).

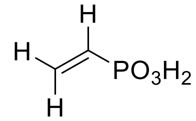

Vinylphosphonic acid (7a). ^{31}P NMR (D_2O) δ: 12.9 (ddd, $^2J_{H\text{-}P}$ = 20.6 Hz, $^3J_{H\text{-}P\,(cis)}$ = 23.4 Hz, $^3J_{H\text{-}P\,(trans)}$ = 46.8 Hz); ^{1}H NMR (D_2O) δ: 6.01 (ddd, 1H, $^3J_{H\text{-}H}$ = 12.7 Hz, $^3J_{H\text{-}H}$ = 18.8 Hz, $^2J_{H\text{-}P}$ = 20.5 Hz), 5.75 (ddd, 1H, $^2J_{H\text{-}H}$ = 2.5 Hz, $^3J_{H\text{-}H}$ = 18.9 Hz, $^3J_{H\text{-}P\,(cis)}$ = 23.3 Hz), 5.66 (ddd, 1H, $^2J_{H\text{-}H}$ = 2.5 Hz, $^3J_{H\text{-}H}$ = 12.7 Hz, $^3J_{H\text{-}P\,(trans)}$ = 46.3 Hz).

Table S3. Comparison of chemical shifts and coupling constants on ^{1}H and ^{31}P NMR spectra of the reaction products of 1-aminoethylphosphonic acid **(1a)** with $NaNO_2$ in water with compounds described in the literature.

**Structure**	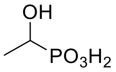		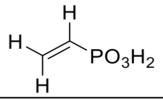	
**Source**	**ABr1178**	**Reference [8]**	**ABr1178**	**Reference [6]**
**Solvent**	**D_2O**	**D_2O**	**D_2O**	**$CDCl_3$**
**^{31}P NMR**	21.33 (dq, *J* = 5.6, 15.4 Hz)	19.6	12.9 (ddd, *J* = 20.6, 23.4, 46.8 Hz)	17.3 (ddd, *J* = 20.0, 20.5, 51.3 Hz)
**^{1}H NMR**	3.70 (dq, 1H, *J* = 5.9, 7.0 Hz)	3.53-3.58 (m, 1H)	6.01 (ddd, 1H, *J* = 12.7, 18.8, 20.5 Hz)	6.31 (ddd, 1H, *J* = 20.5, 16.2, 4.5 Hz)
	1.19 (dd, 3H, *J* = 7.0, 15.9 Hz)	1.12 (dd, *J* = 7.2, 14.4 Hz, 3H)	5.75 (ddd, 1H, *J* = 2.5, 18.9, 23.3 Hz)	6.05 (ddd, 1H, *J* = 51.3, 11.2, 4.5 Hz)
			5.66 (ddd, 1H, *J* = 2.5, 12.7, 46.3 Hz)	6.02 (ddd, 1H, *J* = 20.0, 16.2, 11.2 Hz)

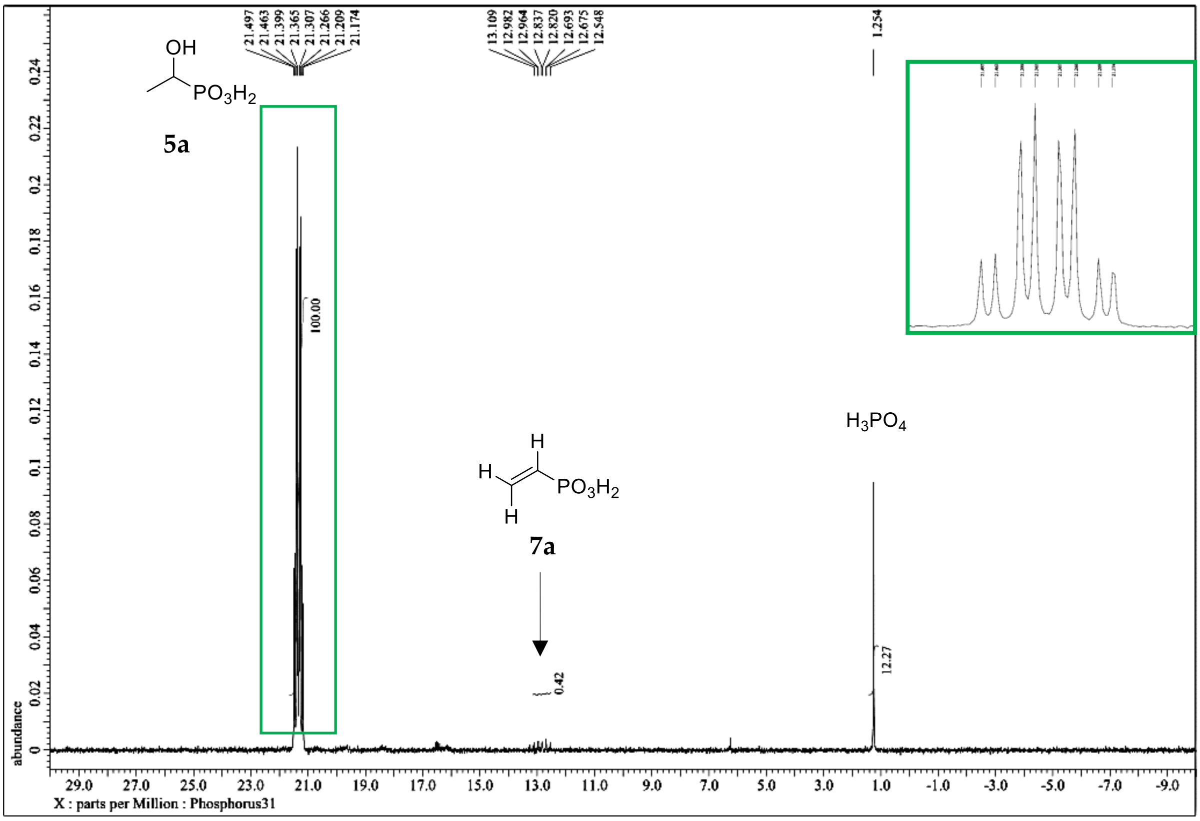

Figure S12. ^{31}P NMR (D_2O, 162MHz) spectra of the crude post-reaction mixture of 1-aminoethylphosphonic acid **(1a)** with $NaNO_2$ after 312 h.

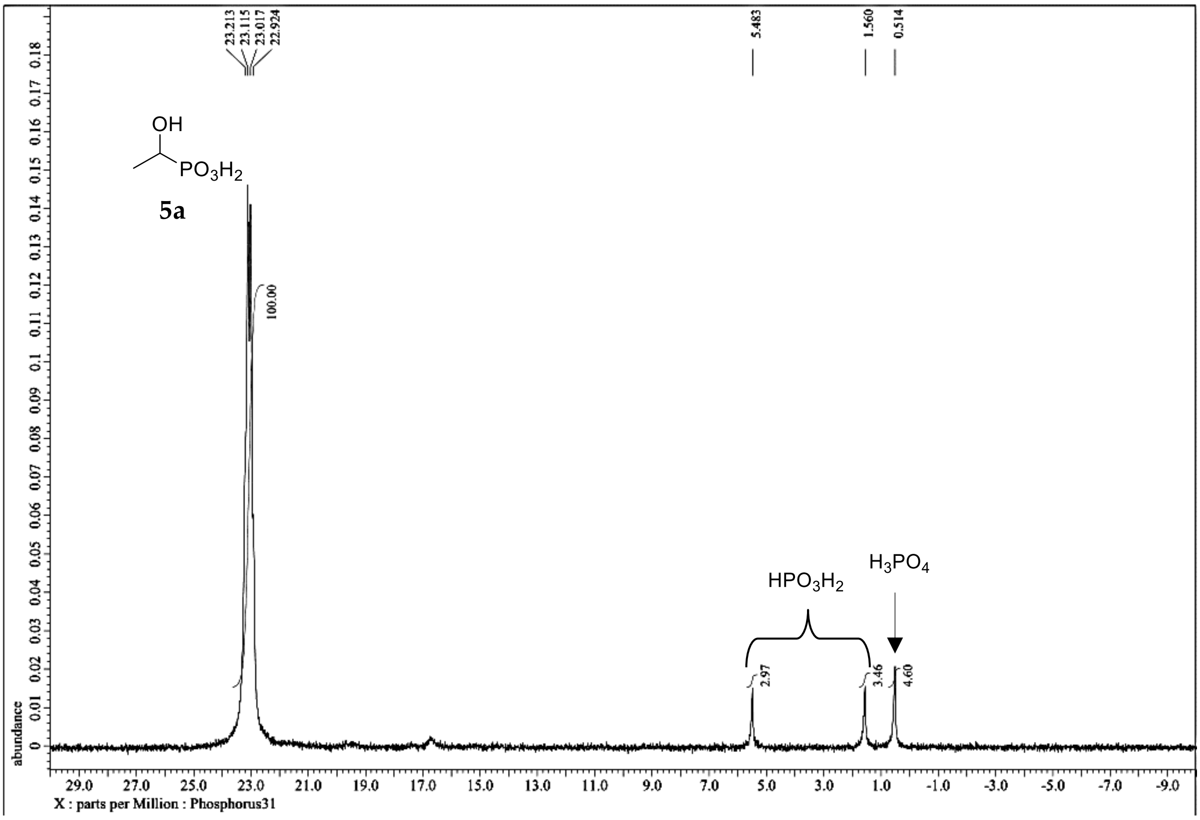

Figure S13. ^{31}P NMR (D_2O, 162MHz) spectra of the crude post-reaction mixture of 1-aminoethylphosphonic acid **(1a)** with $NaNO_2$ after 312 h with addition of 1-hydroxyethylphosphonic acid **(5a)** as standard.

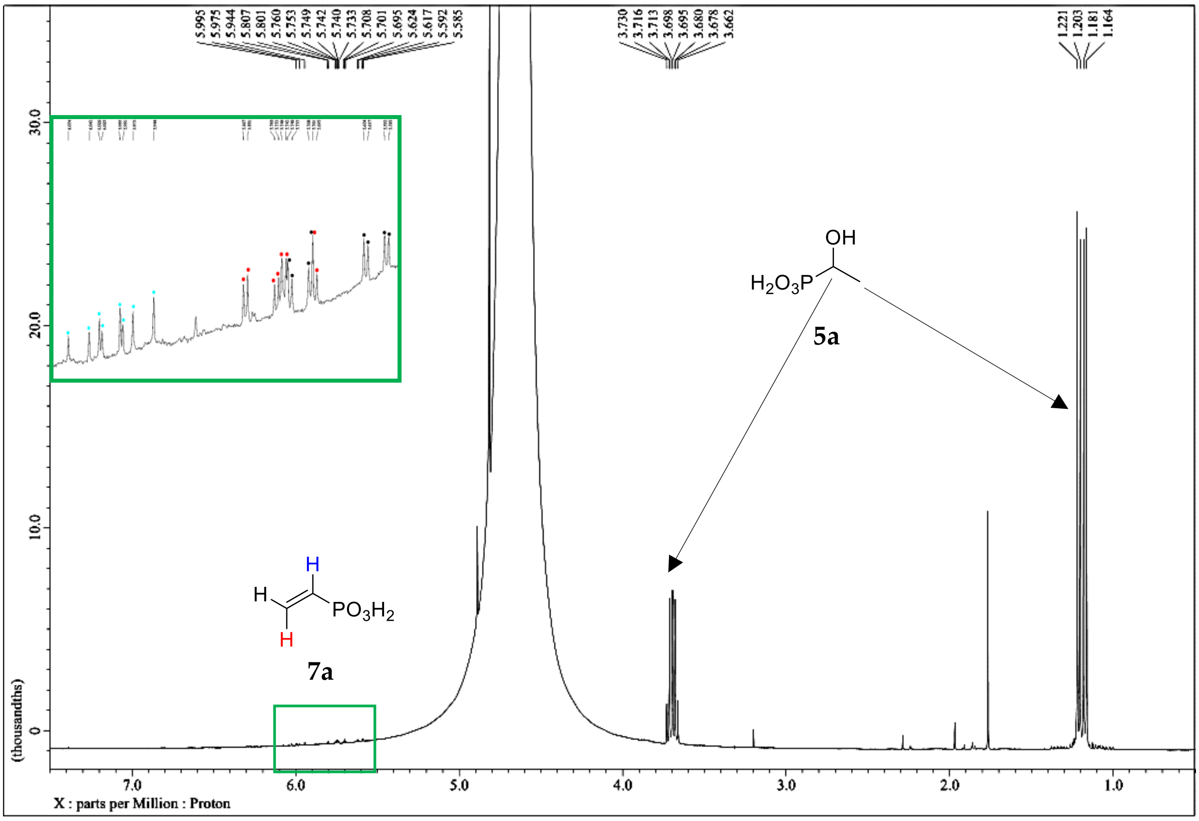

Figure S14. ^{1}H NMR (D_2O, 400MHz) spectra of the crude post-reaction mixture of 1-aminoethylphosphonic acid **(1a)** with $NaNO_2$ after 312 h.

*3.2. ABr1204. Deamination of **1m** in Water*

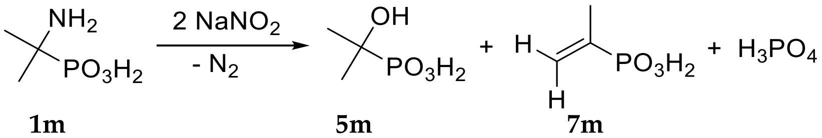

1m **5m** **7m**

24 h	α = 0.98	84%	13%	1%
192 h	α = 1.00	86%	12%	2%
δ_P [ppm]	16.83 (septet)	23.84 (septet)	15.31 (ddq)	1.15 (s)

After 24 h in 21 °C, post-reaction mixture contained 1-hydroxy-1-methylethylphosphonic acid **(5m)** (84%molP), 1-methylvinylphosphonic acid **(7m)** (13%molP), orthophosphoric acid (1%molP) and unreacted substrate **1m** (2%molP).

After 192 h in 21 °C, post-reaction mixture contained 1-hydroxy-1-methylethylphosphonic acid **(5m)** (86%molP), 1-methylvinylphosphonic acid **(7m)** (12%molP) and orthophosphoric acid (2%molP) (Figure S15 and Figure S17).

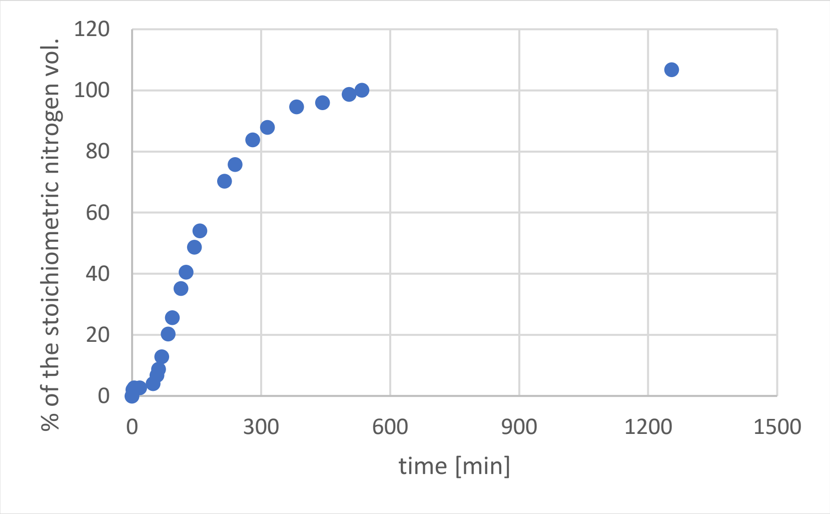

Chart 2. Dependence of the volume of the released gas (in % of the stoichiometric nitrogen volume) on the time in the reaction of 1-amino-1-methylethylphosphonic acid **(1m)** with $NaNO_2$ in water.

The presence of 1-hydroxy-1-methylethylphosphonic acid **(5m)** was confirmed by re-measuring NMR spectra with addition of reference material—1-hydroxy-1-methylethylphoshonic acid **(5m)** (Figure S16 and Figure S18).

1-Methylvinylphosphonic acid (**7m)** was identified by comparing chemical shifts, multiplecity and coupling constants on ^{1}H and ^{31}P NMR spectra with description in the literature (Table S1).

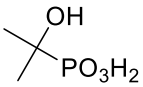

1-Hydroxy-1-methylethylphosphonic acid (5m). ^{31}P NMR (D_2O): δ 23.84 (septet, $^{3}J_{H\text{-}P}$ = 14.0 Hz); ^{1}H NMR (D_2O) δ: 1.23 (d, 6H, $^{3}J_{H\text{-}P}$ = 13.8 Hz).

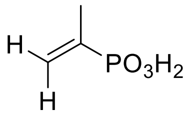

1-Methylvinylphosphonic acid (7m). ^{31}P NMR (D_2O): δ 15.21 (ddq, $^3J_{H\text{-}P\,(trans)}$ = 43.9Hz, $^3J_{H\text{-}P\,(cis)}$ = 20.6, $^3J_{H\text{-}P}$ = 13.1 Hz); ^{1}H NMR (D_2O) δ: 5.46 (ddq, 1H, $^3J_{H\text{-}P\,(cis)}$ = 20.3 Hz, $^2J_{H\text{-}H}$ = 2.1 Hz, $^4J_{H\text{-}H}$ = 1.2 Hz), 5.36 (ddq, 1H, $^3J_{H\text{-}P\,(trans)}$ = 43.4 Hz, $^2J_{H\text{-}H}$ = 1.8 Hz, $^4J_{H\text{-}H}$ = 1.8 Hz), 1.77 (dt, 3H, $^3J_{H\text{-}P}$ = 13.1 Hz, $^4J_{H\text{-}H}$ = 1.2 Hz).

Table S4. Comparison of chemical shifts and coupling constants on ^{1}H and ^{31}P NMR spectra of 1-methylvinylphosphonic acid **(7m)** description in the literature.

**Structure**	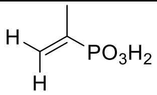	
**Source**	**ABr1204**	**Reference [10]**
**Solvent**	**D_2O**	**D_2O**
**^{31}P NMR**	15.21 (ddq, *J* = 43.9, 20.6, 13.1 Hz)	18.34
**^{1}H NMR**	5.46 (ddq, 1H, *J* = 20.3, 2.1, 1.2 Hz)	5.32–5.47 (q, 2H)
	5.36 (tq, 1H, *J* = 43.4, 1.8 Hz)	
	1.77 (dt, 3H, *J* = 13.1, 1.2 Hz)	1.55/1.58 (d, 3H)

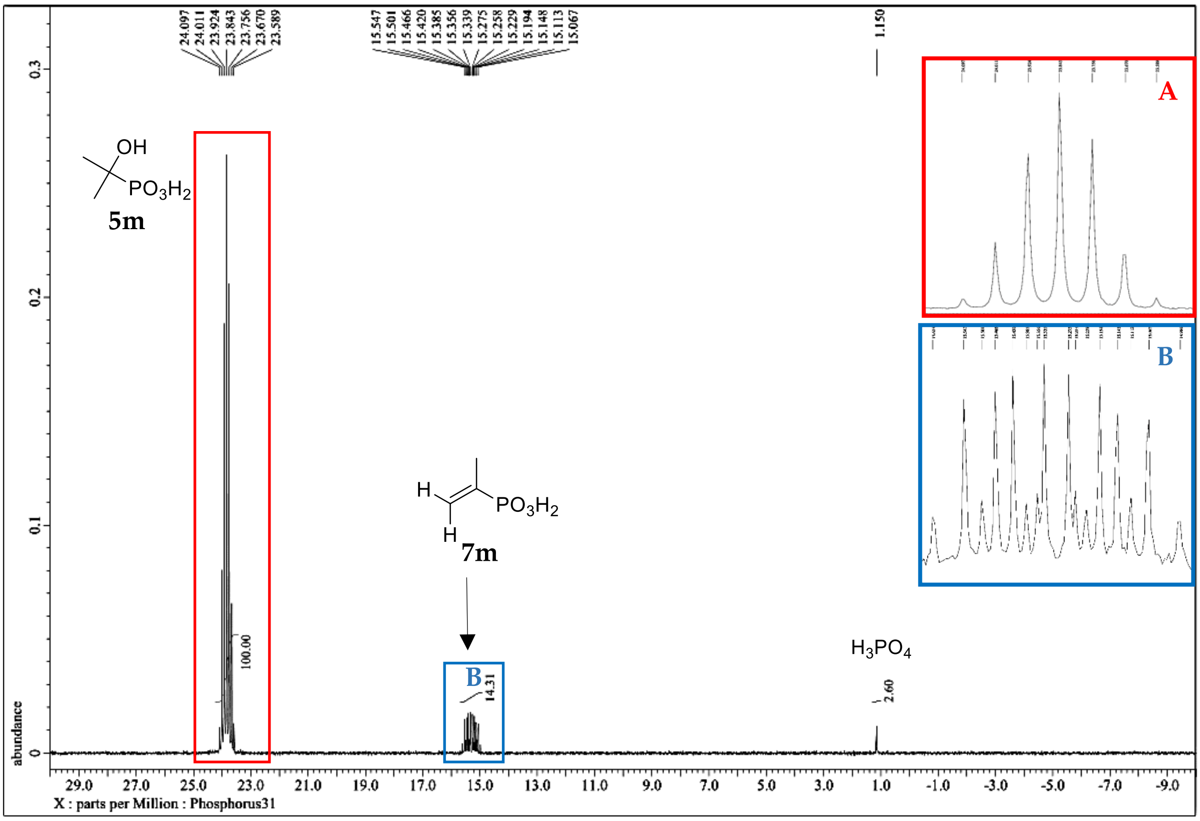

Figure S15. ^{31}P NMR (D_2O, 162MHz) spectra of the crude post-reaction mixture of 1-amino-1-methylethylphosphonic acid **(1m)** with $NaNO_2$ after 192 h**.**

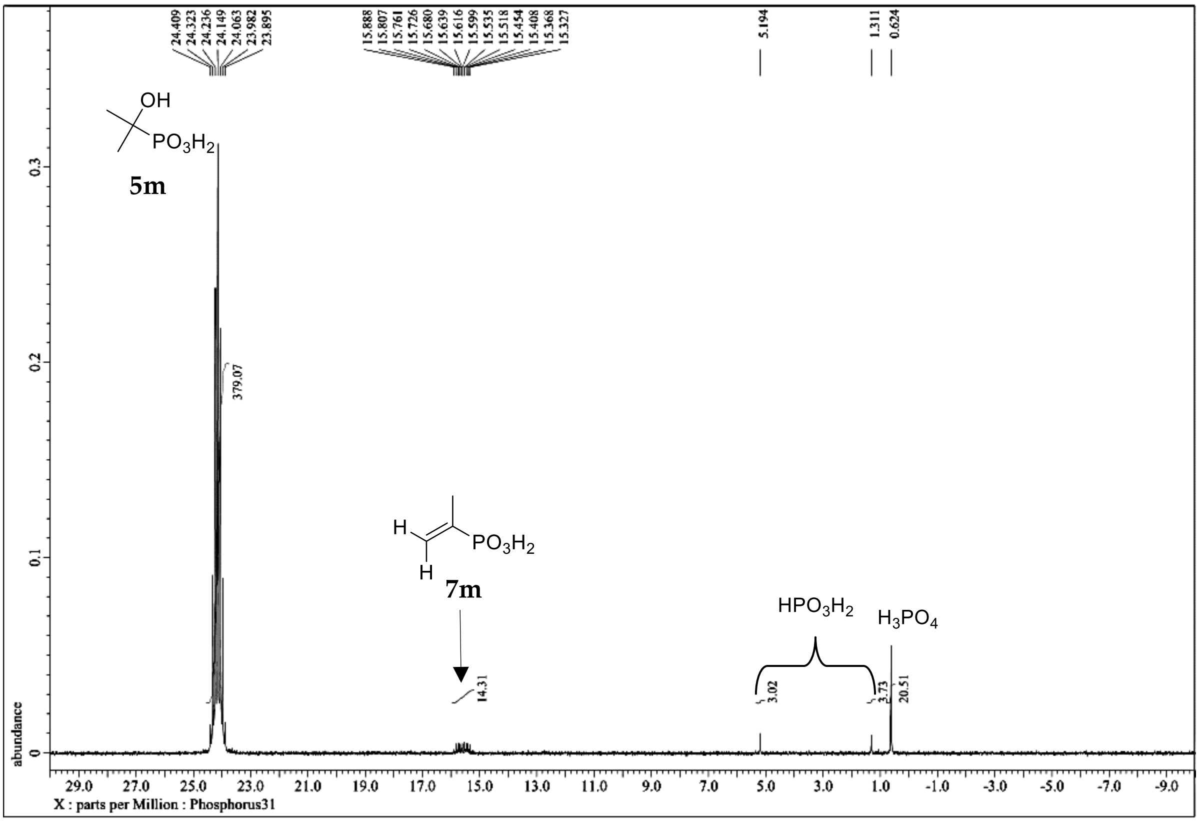

Figure S16. ^{31}P NMR (D_2O, 162MHz) spectra of the crude post-reaction mixture of 1-amino-1-methylethylphosphonic acid **(1m)** with $NaNO_2$ after 192 h with addition of 1-hydroxy-1-methylethylphosphonic acid **(5m)** as reference.

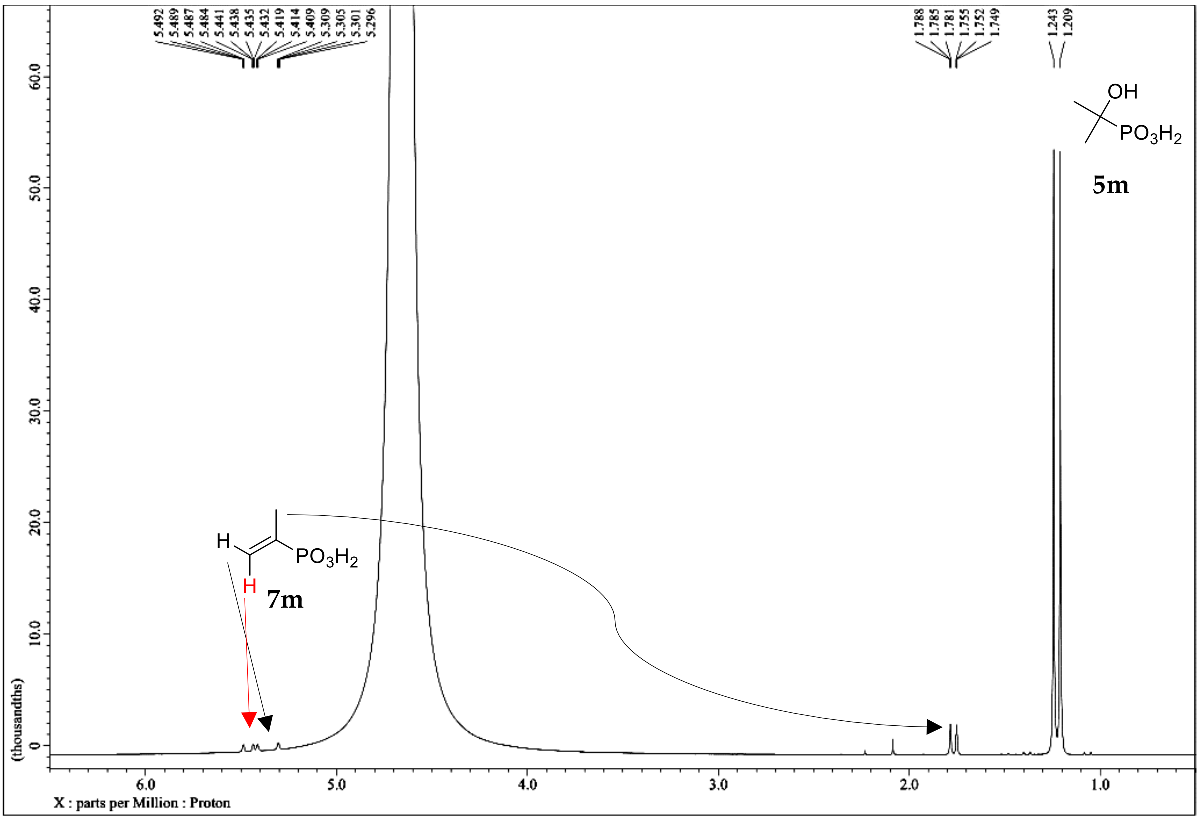

Figure S17. ^{1}H NMR (D_2O, 400MHz) spectra of the crude post-reaction mixture of 1-amino-1-methylethylphosphonic acid **(1m)** with $NaNO_2$ after 192 h.

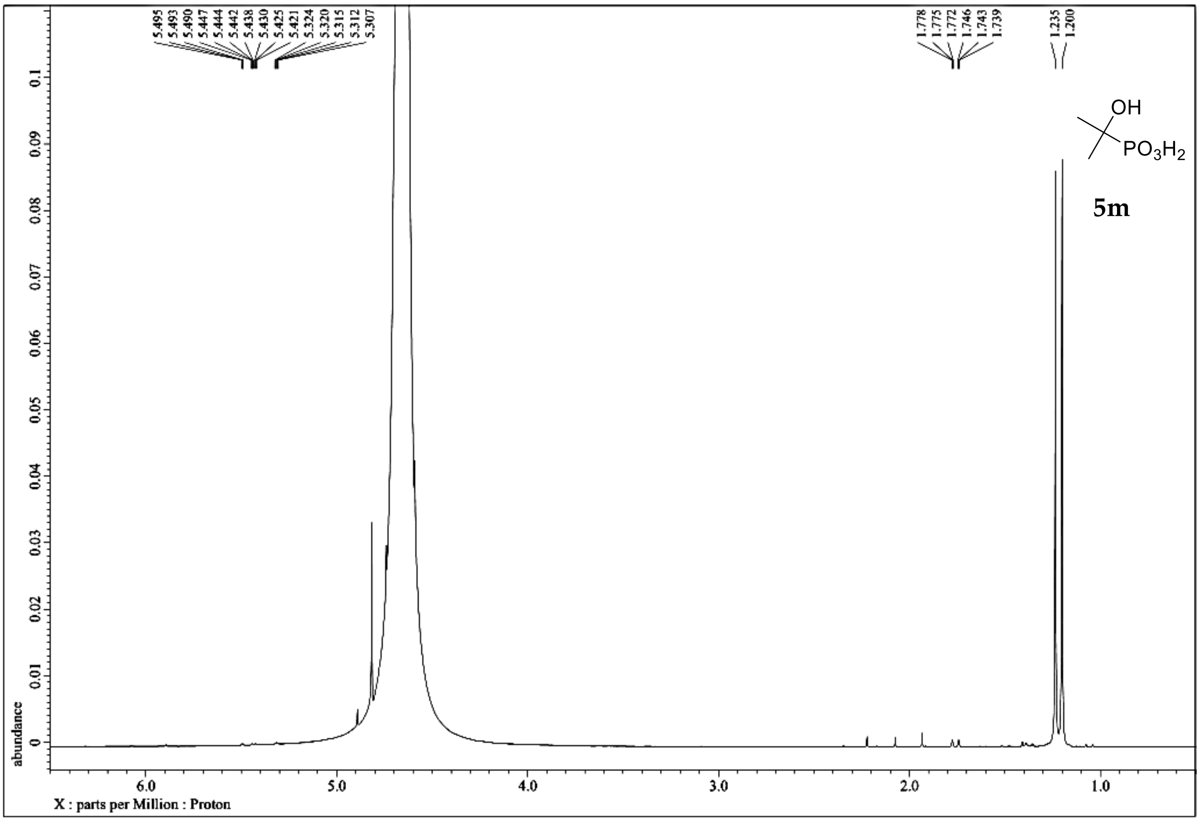

Figure S18. ^{1}H NMR (D_2O, 400MHz) spectra of the crude post-reaction mixture of 1-amino-1-methylethylphosphonic acid **(1m)** with $NaNO_2$ after 192 h with addition of 1-hydroxy-1-methylethylphosphonic acid **(5m)** as reference.

3.3. ABr1206. Deamination of ***1o*** *in Water*

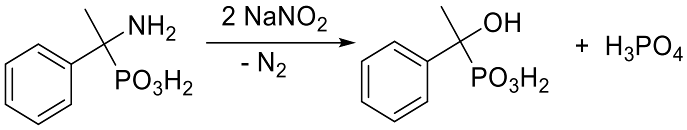

1o **5o**

48h	$\alpha = 1.00$	98%	2%
δ_P [ppm]		20.28 (q)	0.88 (s)

After 48h at 21 °C, post-reaction mixture contained 1-hydroxy-1-phenylethylphosphonic acid **(5o)** (98%molP) and orthophosphoric acid (2%molP) (Figure S19 and Figure S21).

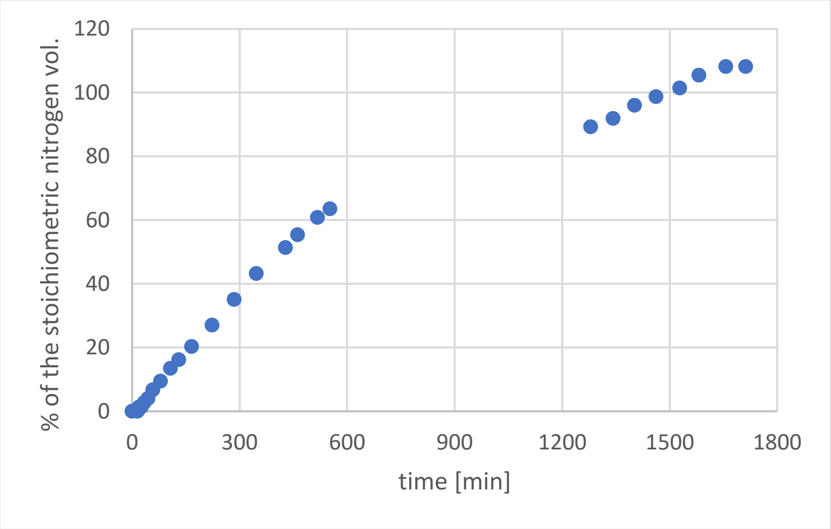

Chart 3. Dependence of the volume of the released gas (in % of the stoichiometric nitrogen volume) on the time in the reaction of 1-amino-1-phenylethylphosphonic acid **(1o)** with $NaNO_2$ in water.

The presence of 1-hydroxy-1-phenylethylphosphonic acid **(5o)** was confirmed by re-measuring NMR spectra (Figure S20 and Figure S22) with addition of 1-hydroxy-1-phenylethylphosphonic acid **(5o)** as reference material.

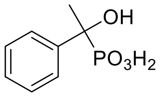

1-Hydroxy-1-phenylethylphosphonic acid (5o). ^{31}P NMR (D_2O): δ 20.28 (q, $^3J_{H\text{-}P}$ = 14.0 Hz); ^{1}H NMR (D_2O) δ: 7.43-7.48 (m, 2H), 7.26-7.32 (m, 2H), 7.18-7.24 (m, 1H), 1.63 (d, 6H, $^3J_{H\text{-}P}$ = 13.8 Hz).

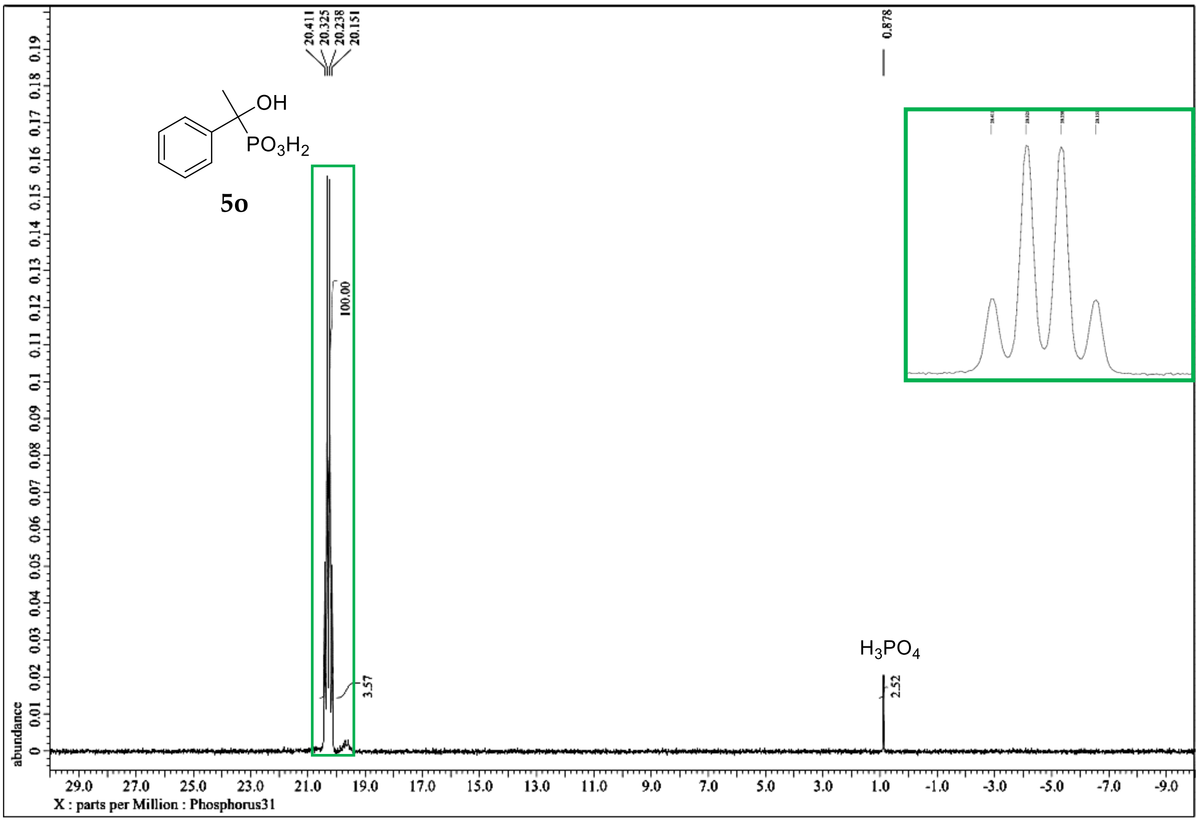

Figure S19. ^{31}P NMR (D_2O, 162MHz) spectra of the crude post-reaction mixture of 1-amino-1-phenylethylphosphonic acid **(1o)** with $NaNO_2$ after 48h.

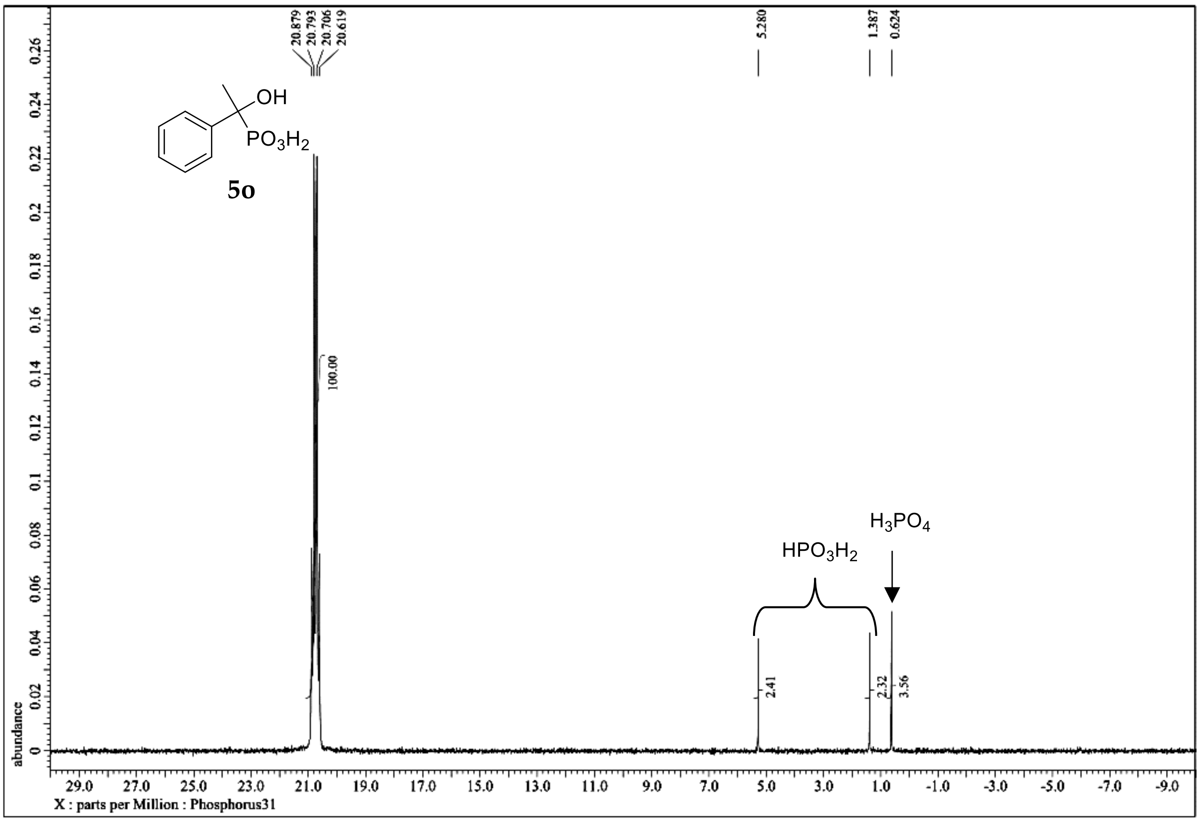

Figure S20. ^{31}P NMR (D_2O, 162MHz) spectra of the crude post-reaction mixture of 1-amino-1-phenylethylphosphonic acid **(1o)** with $NaNO_2$ after 48h with addition of 1-hydroxy-1-phenylethylphosphonic acid **(5o)** as reference material.

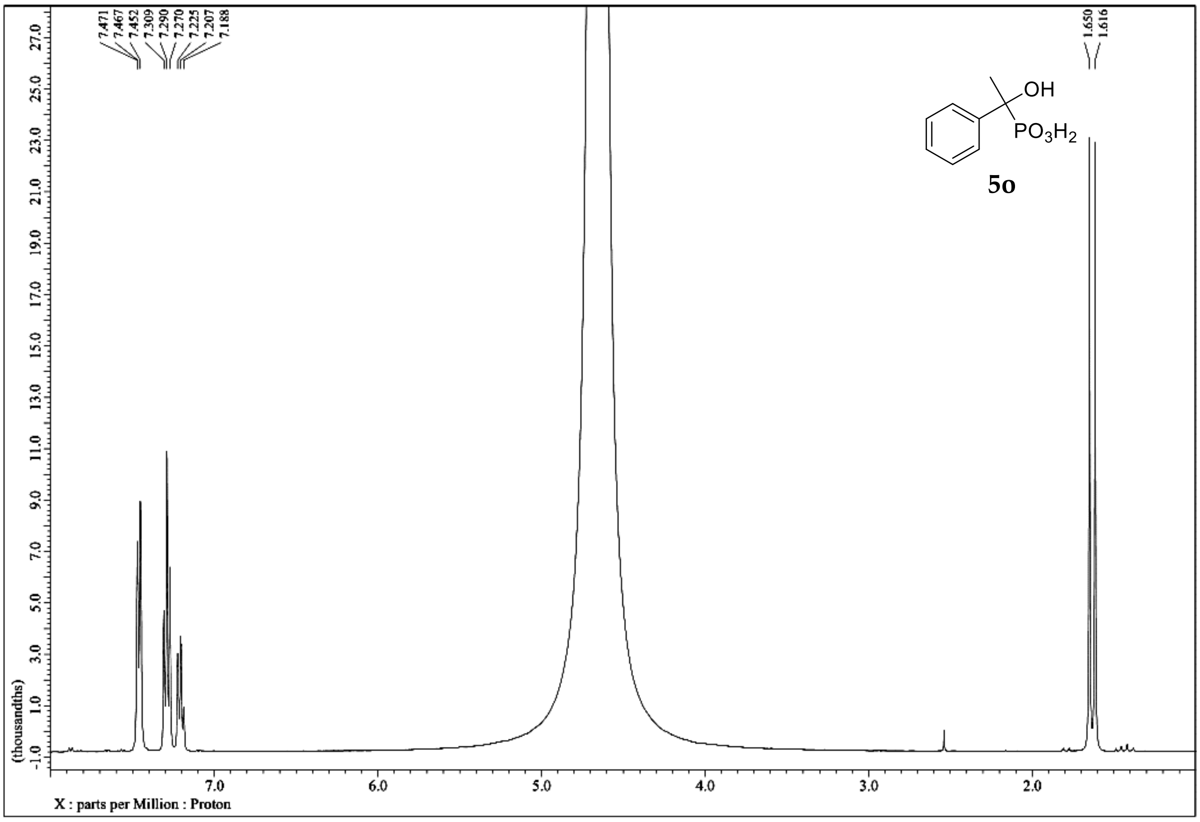

Figure S21. ^{1}H NMR (D_2O, 400MHz) spectra of the crude post-reaction mixture of 1-amino-1-phenylethylphosphonic acid **(1o)** with $NaNO_2$ after 48h.

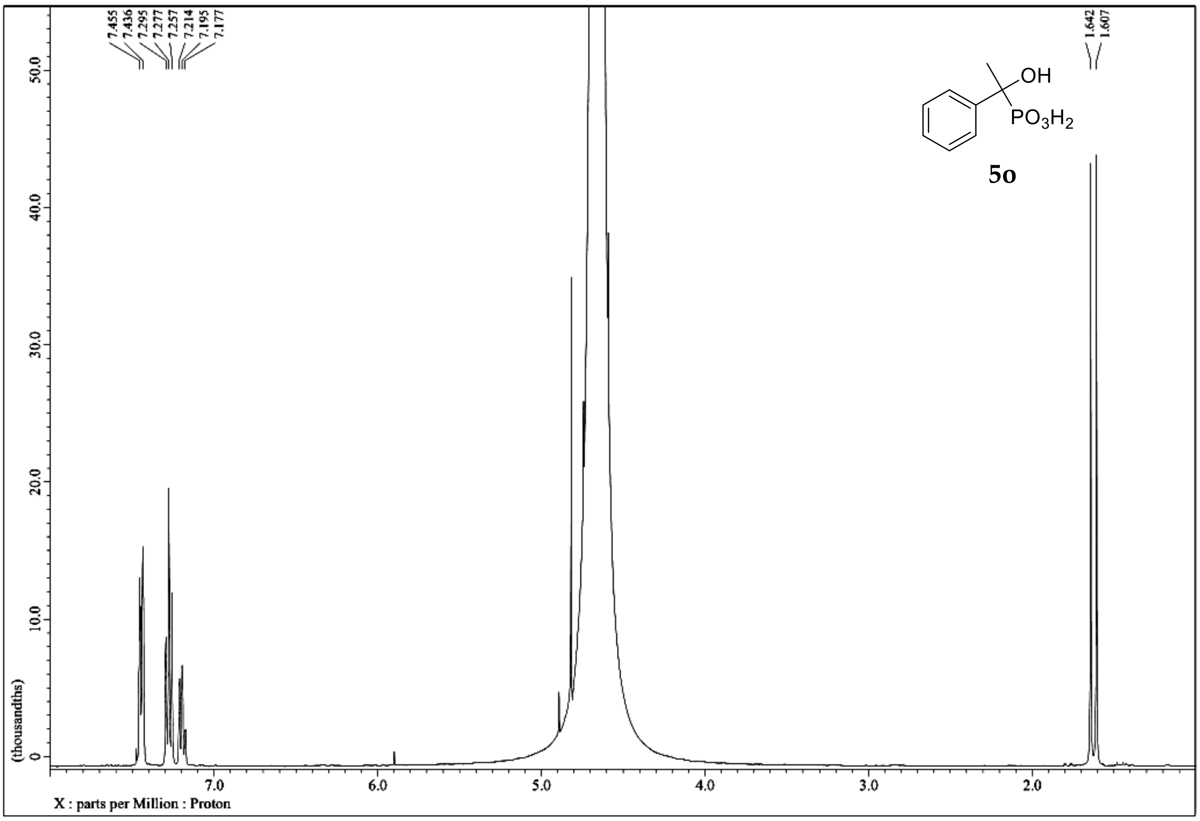

Figure S22. ^{1}H NMR (D_2O, 400MHz) spectra of the crude post-reaction mixture of 1-amino-1-phenylethylphosphonic acid **(1o)** with $NaNO_2$ after 48h with addition of 1-hydroxy-1-phenylethylphosphonic acid **(5o)** as reference material.

3.4. ABr1208. Deamination of **1b** in Water

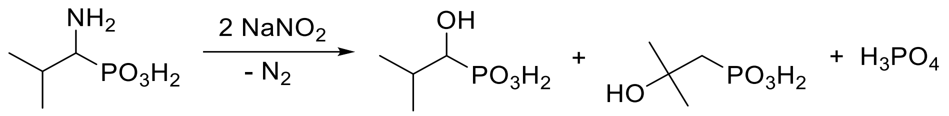

1b **5b** **5′b**

24 h	α = 1.00	34%	5%	59%
192 h	α = 1.00	33%	5%	58%
δ_P [ppm]		20.57 (dd)	21.24 (t)	0.84 (s)

After 24 h at 21 °C, post-reaction mixture contained orthophosphoric acid (59%molP), 1-hydroxy-2-methylpropylphosphonic acid **(5b)** (34%molP), 2-hydroxy-2-methylpropylphosphonic acid **(5′b)** (5%molP) and unidentified compound (2%molP).

After 192 h , post-reaction mixture contained orthophosphoric acid (58%molP), 1-hydroxy-2-methylpropylphosphonic acid **(5b)** (33%molP), 2-hydroxy-2-methylpropylphosphonic acid **(5′b)** (5%molP) and 3 unidentified compounds (2%molP, 1%molP, >1%molP) (Figure S23 and Figure S25).

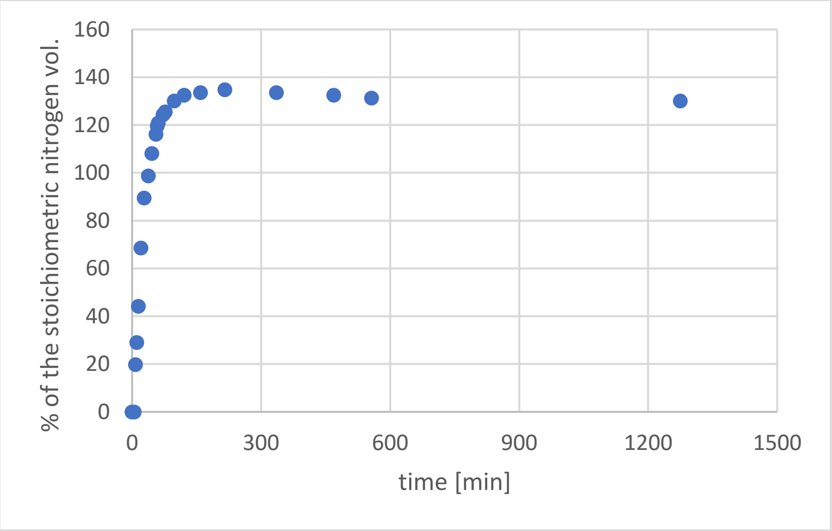

Chart 4. Dependence of the volume of the released gas (in % of the stoichiometric nitrogen volume) on the time in the reaction of 1-amino-2-methylpropylphosphonic acid **(1b)** with $NaNO_2$ in water.

The presence of 1-hydroxy-2-methylpropylphosphonic acid **(5b)** was confirmed by re-measuring NMR spectra (Figure S24 and Figure S26) with addition of 1-hydroxy-2-methylpropylphosphonic acid **(5b)** as reference material.

2-Hydroxy-2-methylpropylphosphonic acid **(5′b)** has been not described in the chemical literature, therefore it was identified by analysis of the signals multiplecity on the ^{1}H and ^{31}P NMR spectra and by comparing spectra with spectra of similar 2-hydroxypropylphosphonic acid (Table S5).

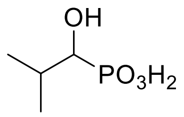

1-Hydroxy-2-methylpropylphosphonic acid (5b). ^{31}P NMR (D_2O): δ 20.57 (dd, $^{2}J_{H\text{-}P}$ = 8.4 Hz, $^{3}J_{H\text{-}P}$ = 7.5 Hz); ^{1}H NMR (D_2O) δ: 3.56 (dd, 1H, $^{2}J_{H\text{-}P}$ = 8.6 Hz, $^{3}J_{H\text{-}H}$ = 5.5Hz), 1.87 (doublet of doublets of septets, $^{3}J_{H\text{-}H}$ = 5.5Hz, $^{3}J_{H\text{-}P}$ = 6.8 Hz, $^{3}J_{H\text{-}H}$ = 5.5 Hz), 0.90 (d, 3H, $^{3}J_{H\text{-}H}$ = 7.0Hz), 0.87 (d, 3H, $^{3}J_{H\text{-}H}$ = 7.0Hz)

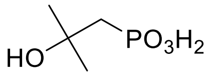

2-Hydroxy-2-methylpropylphosphonic acid (5′b). ^{31}P NMR (D_2O): δ 21.24 (t, $^{2}J_{H\text{-}P}$ = 17.8 Hz); ^{1}H NMR (D_2O) δ: 1.83 (d, 3H, $^{2}J_{H\text{-}P}$ = 17.7 Hz), 1.22 (s, 6H).

Table S5. Comparison of chemical shifts and coupling constants on ^{1}H and ^{31}P NMR spectra of 2-hydroxy-2-methylpropylphosphonic acid **(5′b)** with spectra of 2-hydroxypropylphosphonic acid described in the literature.

**Structure**	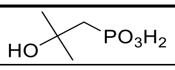	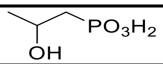
**Source**	**ABr1208**	**Reference [9]**
**Solvent**	**D_2O**	**D_2O**
**^{31}P NMR**	21.24 (t, *J* = 17.8 Hz)	19.9
**^{1}H NMR**		3.80 (1H, dm, *J* = 6.5 Hz)
	1.83 (d, 3H, *J* = 17.7 Hz)	1.39 (2H, ddd, *J* = 18.0, 6.6, 15.3 Hz)
	1.22 (s, 6H)	0.97 (3H, d, *J* = 6.5 Hz)

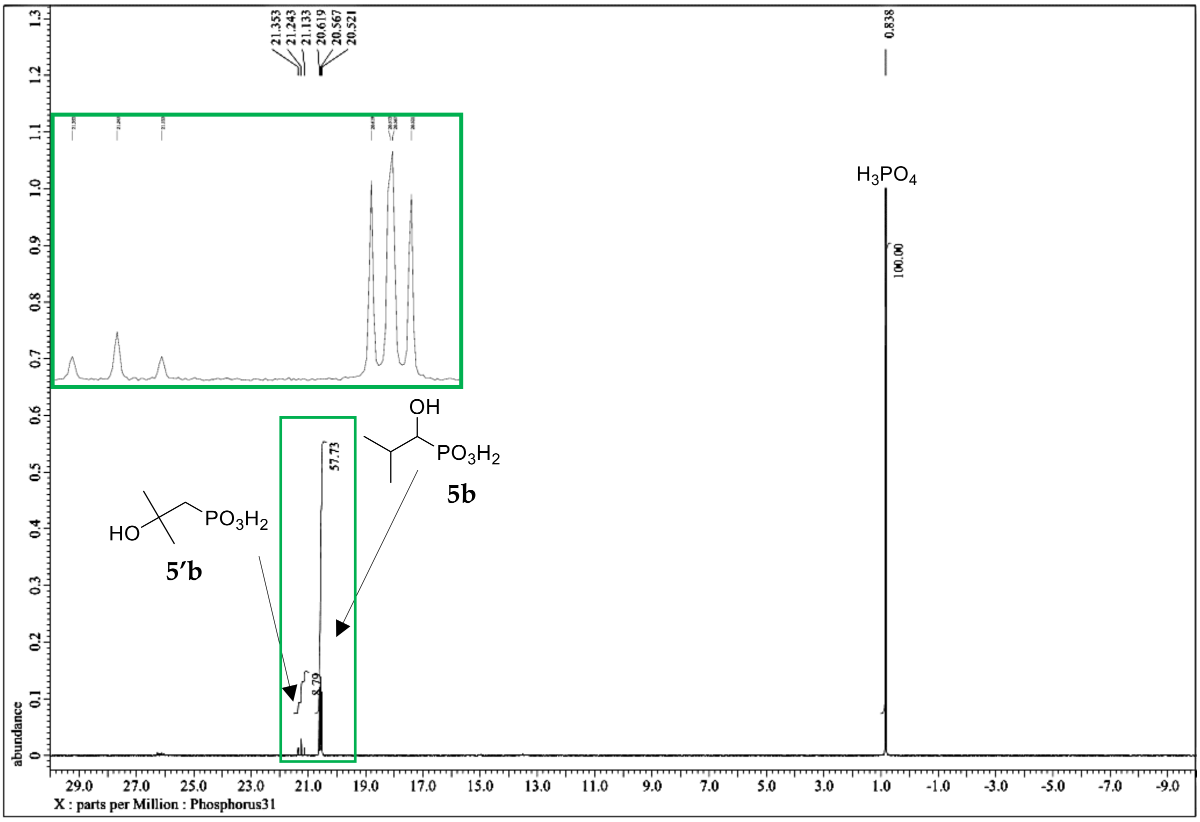

Figure S23. ^{31}P NMR (D_2O, 162MHz) spectra of the crude post-reaction mixture of 1-amino-2-methylpropylphosphonic acid **(1b)** with $NaNO_2$ after 192 h.

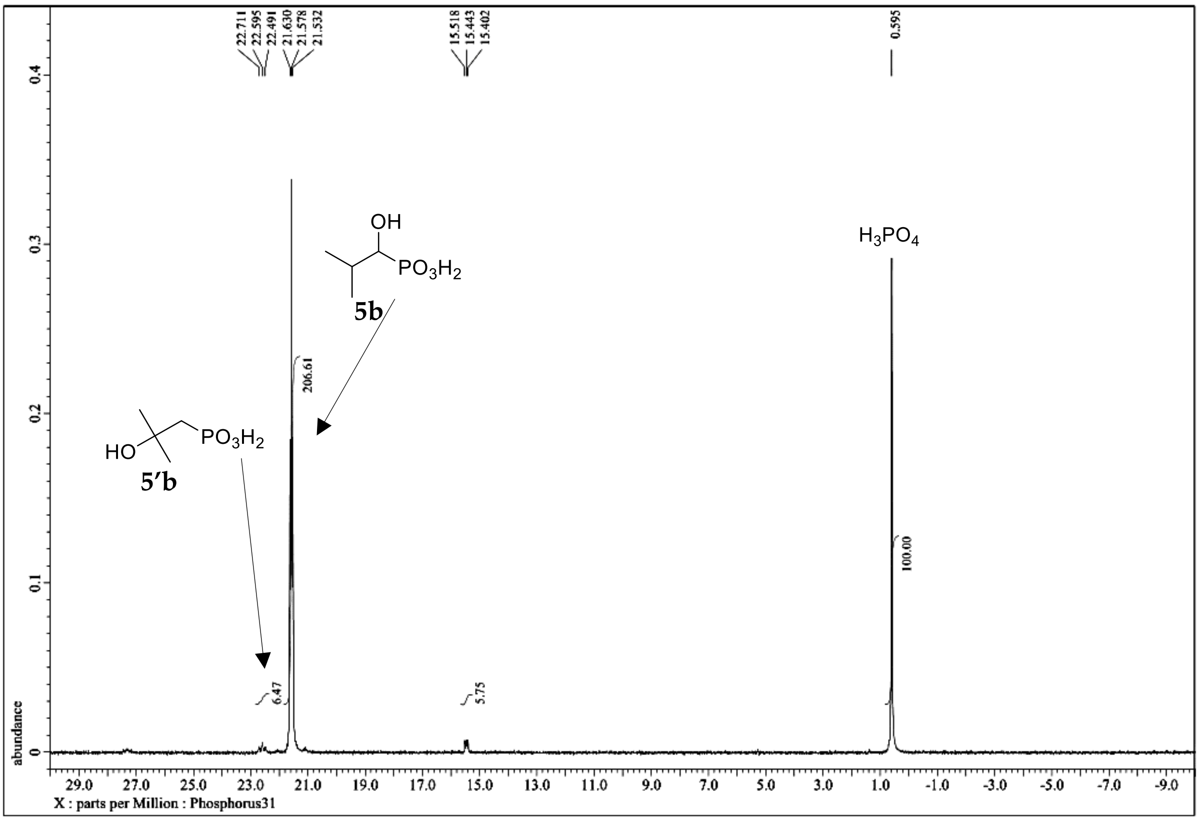

Figure S24. ^{31}P NMR (D_2O, 162MHz) spectra of the crude post-reaction mixture of 1-amino-2-methylpropylphosphonic acid **(1b)** with $NaNO_2$ after 192 h with addition of 1-hydroxy-2-methylpropylphosphonic acid **(5b)** as reference material.

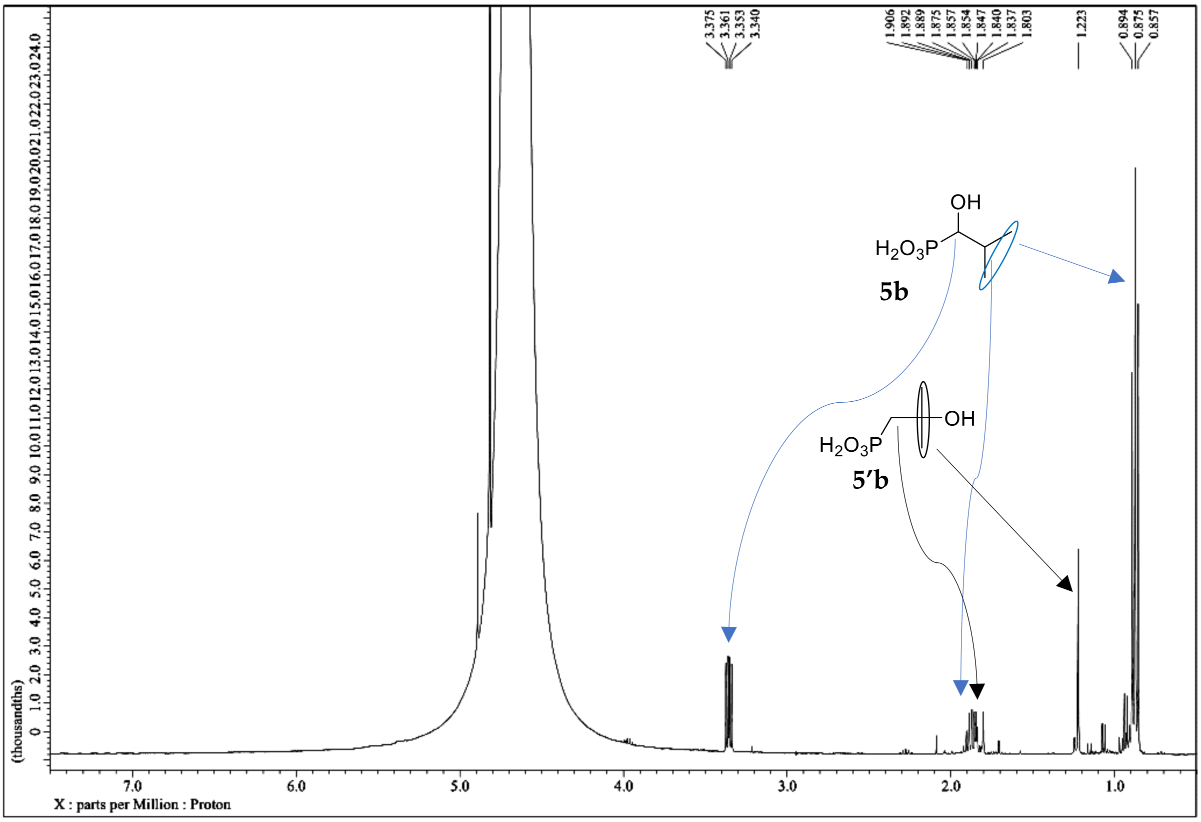

Figure S25. ^{1}H NMR (D_2O, 400MHz) spectra of the crude post-reaction mixture of 1-amino-2-methylpropylphosphonic acid **(1b)** with $NaNO_2$ after 192 h.

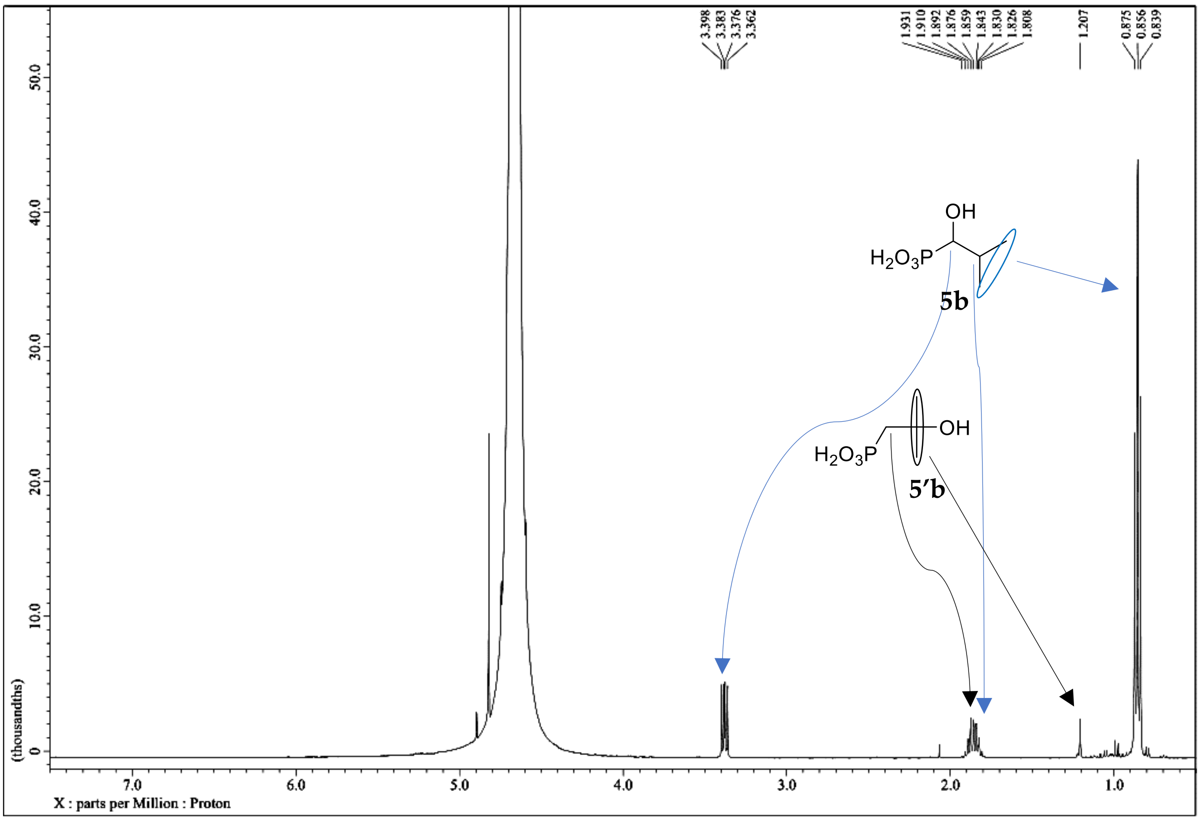

Figure S26. ^{1}H NMR (D_2O, 400MHz) spectra of the crude post-reaction mixture of 1-amino-2-methylpropylphosphonic acid **(1b)** with $NaNO_2$ after 192 h with addition of 1-hydroxy-2-methylpropylphosphonic acid **(5b)** as reference material.

3.5. ABr1210. Deamination of ***1d*** *in Water*

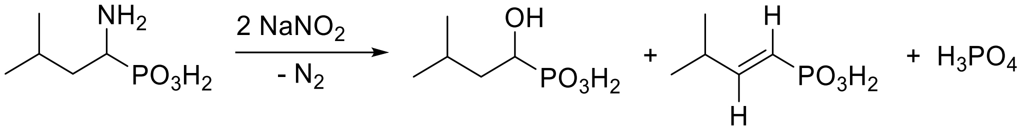

1d **5d** **7d**

24 h	α = 1.00	61%	6%	24%
168 h	α = 1.00	61%	7%	27%
δ_P [ppm]		21.58 (dd)	14.90 (ddd)	1.16 (s)

After 24 h at 21 °C, post-reaction mixture contained 1-hydroxy-3-methylbutylphosphonic acid **(5d)** (61%molP), (*E*)-3-but-1-en-1-ylphosphonic acid **(7d)** (6%molP), orthophosphoric acid (24%molP) and 5 unidentified compounds (9%molP in total).

After 168h post-reaction mixture contained 1-hydroxy-3-methylbutylphosphonic acid **(5d)** (61%molP), (*E*)-3-but-1-en-1-ylphosphonic acid **(7d)** (7%molP), orthophosphoric acid (27%molP) and 4 unidentified compounds (5%molP in total) (Figure S27 and Figure S28).

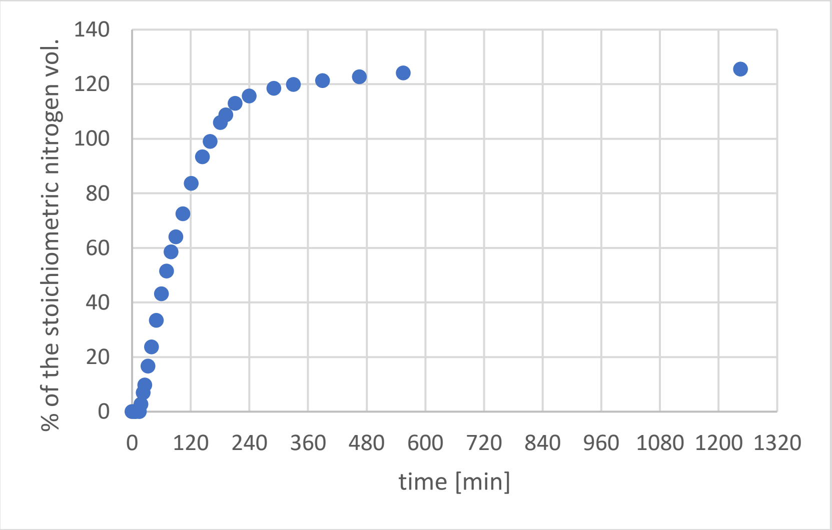

Chart 5. Dependence of the volume of the released gas (in % of the stoichiometric nitrogen volume) on the time in the reaction of 1-amino-3-methylnutylphosphonic acid **(1d)** with $NaNO_2$ in water.

1-Hydroxy-3-methylbutylphosphonic acid **(5d)** was identified by comparing chemical shifts, multiplecity and coupling constants on ^1H and ^{31}P NMR spectra with description in the literature (Table S6). NMR spectra of (*E*)-3-but-1-en-1-ylphosphonic acid **(7d)** were not described in the literature, therefore this compound was identified by comparing signals multiplecity on the ^1H and ^{31}P NMR spectra with spectra of structurally similar (*E*)-prop-1-en-1-ylphosphonic acid (Table S6).

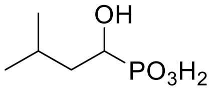

1-Hydroxy-3-methylbutylphosphonic acid (5d). ^{31}P NMR (D_2O): δ 21.61 (dt, $^{2}J_{H\text{-}P}$ = 7.5 Hz, $^{3}J_{H\text{-}P}$ = 4.7 Hz); ^{1}H NMR (D_2O) δ: 3.64 (ddd, 1H, $^{3}J_{H\text{-}H}$ = 11.3 Hz, $^{2}J_{H\text{-}P}$ = 7.8 Hz, $^{3}J_{H\text{-}H}$ = 2.8 Hz), 1.60-1.72 (m, 1H, *J* undetermined), 1.47 (dddd, 1H, $^{2}J_{H\text{-}H}$ = 14.5 Hz, $^{3}J_{H\text{-}H}$ = 11.3 Hz, $J_{H\text{-}H}$ = 8.0 Hz, $^{3}J_{H\text{-}P}$ = 4.0 Hz), 1.32 (dddd, 1H, $^{2}J_{H\text{-}H}$ = 14.6 Hz, $^{3}J_{H\text{-}H}$ = 10.1 Hz, $^{3}J_{H\text{-}P}$ = 4.9 Hz, $^{3}J_{H\text{-}H}$ = 2.8 Hz), 0.81 (d, 3H, $^{3}J_{H\text{-}H}$ = 6.4 Hz), 0.77 (d, 3H, $^{3}J_{H\text{-}H}$ = 6.7 Hz).

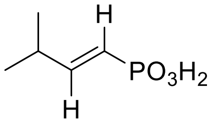

***(E)*-3-But-1-en-1-ylphosphonic acid (7d)**. ^{31}P NMR (D_2O): δ 14.90 (ddd, $^{3}J_{H\text{-}P}$ = 21.5 Hz, $^{2}J_{H\text{-}P}$ = 18.7 Hz, $^{4}J_{H\text{-}P}$ = 1.9 Hz); ^{1}H NMR (D_2O) δ: 6.30 (ddd, 1H, $^{3}J_{H\text{-}P}$ = 21.1 Hz, $^{3}J_{H\text{-}H}$ = 17.1 Hz, $^{3}J_{H\text{-}H}$ = 6.1 Hz), 5.56 (ddd, 1H, $^{2}J_{H\text{-}P}$ = 19.6 Hz, $^{3}J_{H\text{-}H}$ = 17.1 Hz, $^{4}J_{H\text{-}H}$ = 1.5 Hz), 1.74-1.88 (m, 1H, *J* undetermined), 0.87 (d, 6H, $^{3}J_{H\text{-}H}$ = 6.7 Hz).

Table S6. Comparison of chemical shifts and coupling constants on ^{1}H and ^{31}P NMR spectra of the reaction products of 1-amino-3-methylbutylphosphonic acid **(1d)** with $NaNO_2$ with spectra of compounds described in the literature.

**Structure**	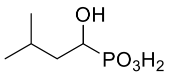		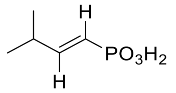	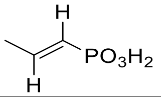
**Source**	**ABr1210**	**Reference [11]**	**ABr1210**	**Reference [12]**
**Solvent**	**D_2O**	**D_2O**	**D_2O**	**D_2O**
**^{31}P NMR**	21.61 (dt, *J* = 7.5, *J* = 4.7 Hz)	25.5	14.90 (ddd, *J* = 21.5, 18.7, 1.9 Hz)	13.31
**^{1}H NMR**	3.64 (ddd, 1H, *J* = 11.3, 7.8, 2.8 Hz)	3.82 (m, 1H)	6.30 (ddd, 1H, *J* = 21.1, 17.1, 6.1 Hz)	6.46 – 6.35 (m, 1H)
	1.60-1.72 (m, 1H, *J* undetermined)	1.73 (m, 1H)	5.56 (ddd, 1H, *J* = 19.6, 17.1, 1.5 Hz)	5.78 – 5.69 (m, 1H)
	1.47 (dddd, 1H, *J* = 14.5, 11.3, 8.0, 4.0 Hz)	1.55 (m, 1H)	1.74-1.88 (m, 1H, *J* undetermined)	1.79 (dt, 3H *J* = 6.5, 2.0 Hz)
	1.32 (dddd, 1H, *J* = 14.6, 10.1, 4.9, 2.8 Hz)	1.40 (m, 1H)	0.87 (d, 6H, *J* = 6.7 Hz)	
	0.81 (d, 3H, *J* = 6.4 Hz)	0.89 (d, 3H, *J* = 6.6 Hz)		
	0.77 (d, 3H, *J* = 6.7 Hz)	0.85 (d, 3H, *J* = 6.6 Hz)		

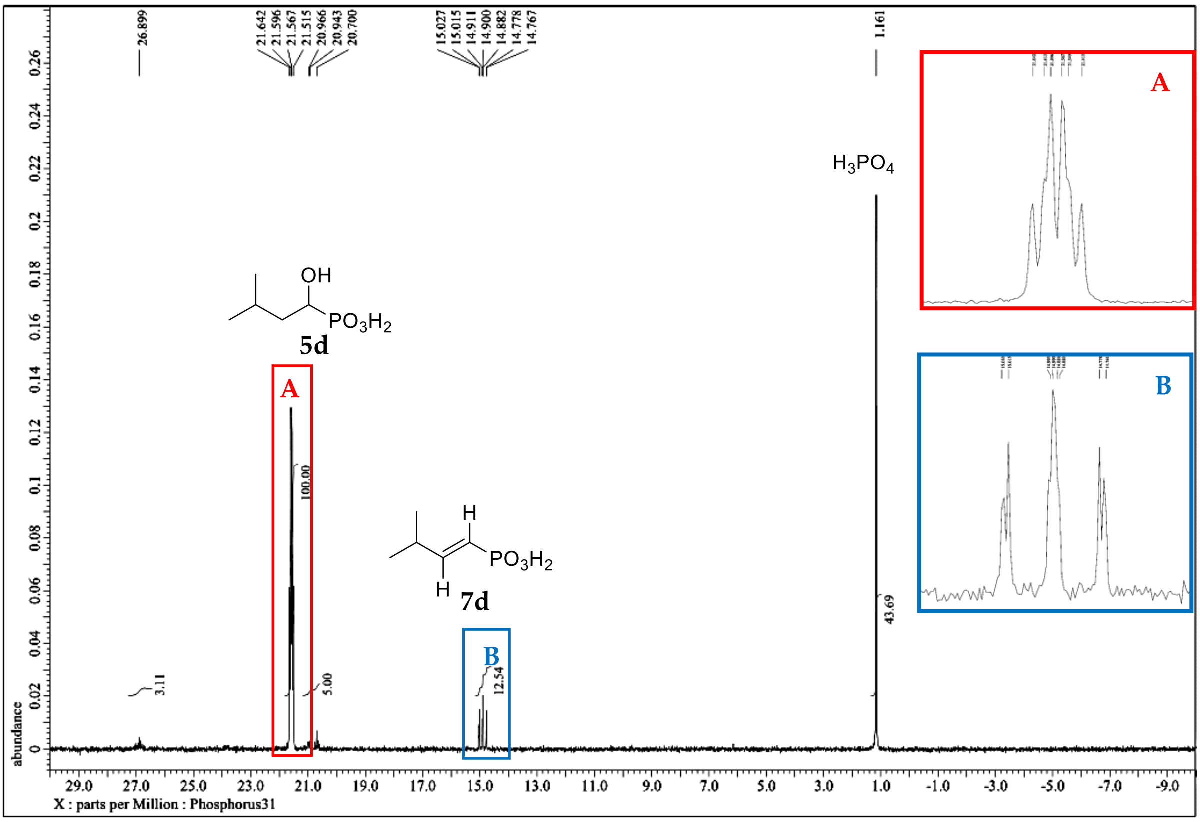

Figure S27. ^{31}P NMR (D_2O, 162MHz) spectra of the crude post-reaction mixture of 1-amino-3-methylbutylphosphonic acid **(1d)** with $NaNO_2$ after 168h.

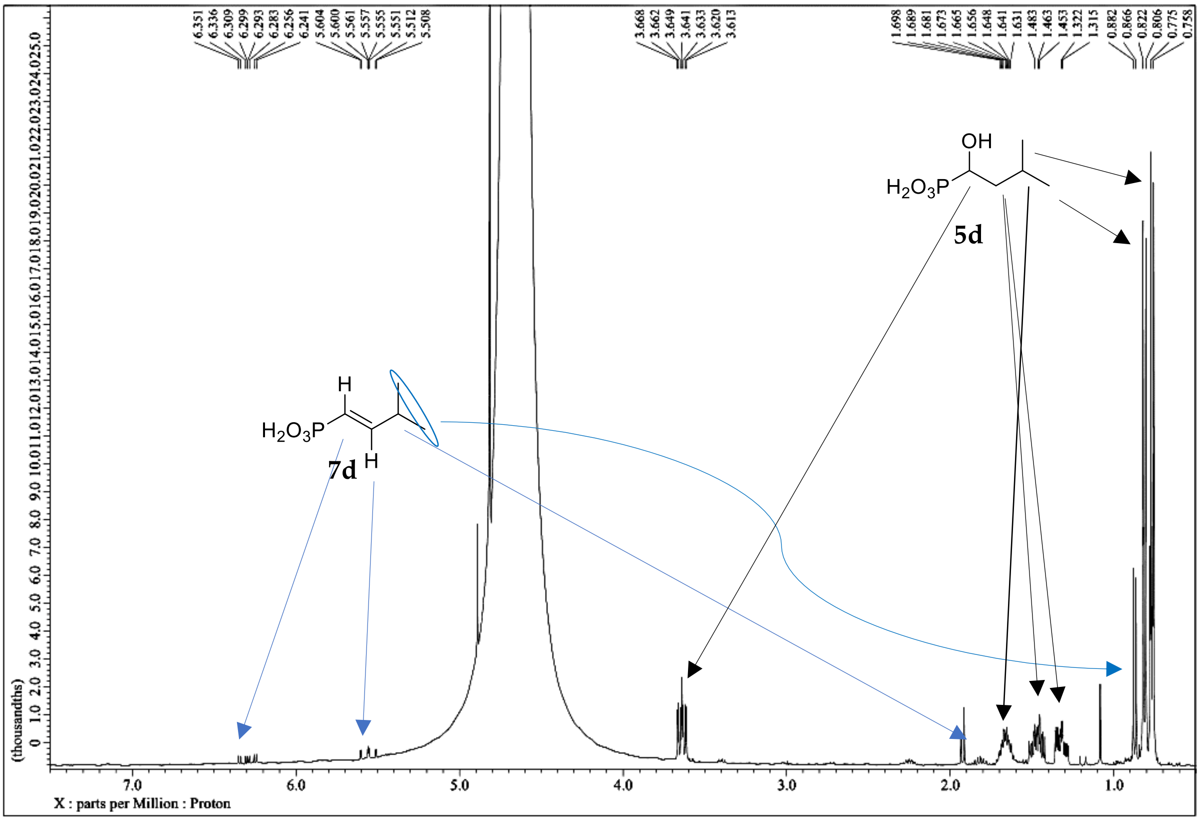

Figure S28. ^{1}H NMR (D_2O, 400MHz) spectra of the crude post-reaction mixture of 1-amino-3-methylbutylphosphonic acid **(1d)** with $NaNO_2$ after 168h.

3.6. *ABr1212. Deamination of* **1c** *in Water*

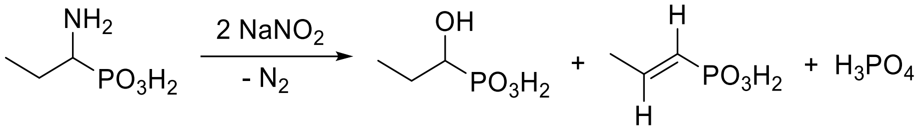

1c **5c** **7c**

24 h	α = 1.00	64%	7%	26%
168h	α = 1.00	63%	7%	28%
δ_P [ppm]		20.96 (dt)	13.84 (ddq)	1.06 (s)

After 24 h at 21 °C, post-reaction mixture contained 1-hydroxypropylphosphonic acid **(5c)** (64%molP), (*E*)-prop-1-en-1ylphosphonic acid **(7c)** (7%molP), orthophosphoric acid (26%molP) and 3 unidentified compounds (3%molP in total).

After 168h post-reaction contained 1-hydroxypropylphosphonic acid **(5c)** (63%molP), (*E*)-prop-1-en-1-ylphosphonic acid **(7c)** (7%molP), orthophosphoric acid (28%molP) and 2 unidentified compounds (3%molP in total) (Figure S29 and Figure S30).

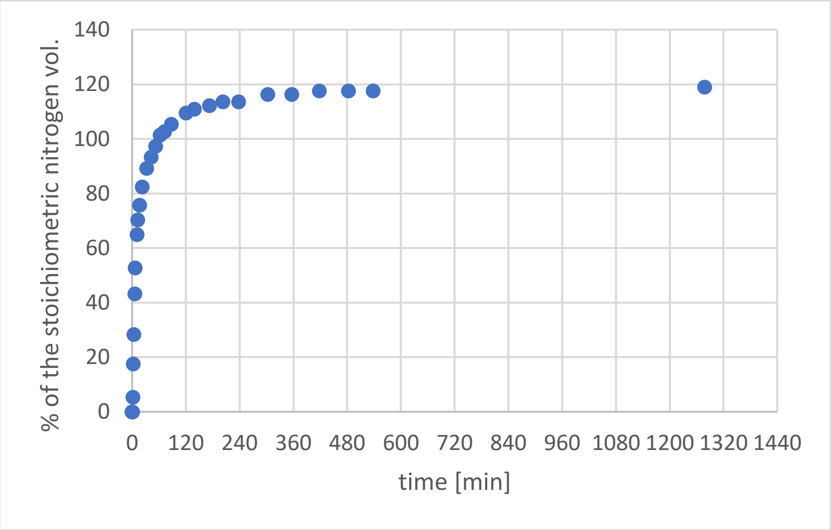

Chart 6. Dependence of the volume of the released gas (in % of the stoichiometric nitrogen volume) on the time in the reaction of 1-aminopropylphosphonic acid **(1c)** with $NaNO_2$ in water.

1-Hydroxypropylphosphonic acid **(5c)** and (*E*)-3-prop-1-en-1-ylphosphonic acid **(7c)** were identified by comparing chemical shifts, multiplecity and coupling constants on ^{1}H and ^{31}P NMR spectra with description in the literature (Table S7).

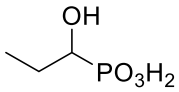

1-Hydroxypropylphosphonic acid (5c). ^{31}P NMR (D_2O): δ 21.61 (dt, $^2J_{H\text{-}P}$ = 7.5 Hz, $^3J_{H\text{-}P}$ = 4.7 Hz); ^{1}H NMR (D_2O) δ: 3.44 (ddd, 1H, $^3J_{H\text{-}H}$ = 10.2 Hz, $^2J_{H\text{-}P}$ = 6.7 Hz, $^3J_{H\text{-}H}$ = 3.4 Hz), 1.60-1.73 (m, 1H, *J* undetermined), 1.44 (dddq, 1H, *J* = 14.0 Hz, *J* = 10.2 Hz, *J* = 9.0 Hz, *J* = 7.0 Hz), 0.88 (t, 3H, $J_{H\text{-}H}$ = 7.3 Hz).

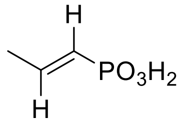

***(E)*-Prop-1-en-1-ylphosphonic acid (7c).** ^{31}P NMR (D_2O): δ 13.84 (ddq, $^{2}J_{H\text{-}P}$ = 20.6 Hz, $^{3}J_{H\text{-}P}$ = 20.3 Hz, $^{4}J_{H\text{-}H}$ = 1.9 Hz); ^{1}H NMR (D_2O) δ: 6.29 (ddq, 1H, $^{3}J_{H\text{-}P}$ = 20.3 Hz, $^{3}J_{H\text{-}H}$ = 17.0 Hz, $^{3}J_{H\text{-}H}$ = 6.5 Hz), 5.63 (ddq, 1H, $^{2}J_{H\text{-}P}$ = 20.6 Hz, $^{3}J_{H\text{-}H}$ = 17.0 Hz, $^{3}J_{H\text{-}H}$ = 1.5 Hz), 1.60-1.73 (m, 3H, *J* undetermined).

Table S7. Comparison of chemical shifts and coupling constants on ^{1}H and ^{31}P NMR spectra of the reaction products of 1-aminopropylphosphonic acid **(1c)** with $NaNO_2$ with compounds described in the literature. .

**Structure**	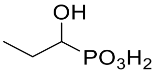		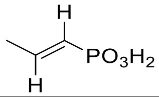	
**Source**	**ABr1212**	**Reference [13]**	**ABr1212**	**Reference [12]**
**Solvent**	**D_2O**	**D_2O**	**D_2O**	**D_2O**
**^{31}P NMR**	21.61 (dt, *J* = 7.5, 4.7 Hz)	23.9	13.84 (ddq, *J* = 20.6, 20.3, 1.9 Hz)	13.31
**^{1}H NMR**	3.44 (ddd, 1H, *J* = 10.2, 6.7, 3.4 Hz)	2.85 - 3.45 (1H, m)	6.29 (ddq, 1H, *J* = 20.3, 17.0, 6.5 Hz)	6.46 – 6.35 (m, 1H)
	1.60-1.73 (m, 1H, *J* undetermined)	1.45 - 2.25 (2H, m)	5.63 (ddq, 1H, *J* = 20.6, 17.0, 1.5 Hz)	5.78 – 5.69 (m, 1H)
	1.44 (dddq, 1H, *J* = 14.0, 10.2, 9.02, 7.0 Hz)		1.60-1.73 (m, 3H)	1.79 (dt, 3H, *J* = 6.5, 2.0 Hz)
	0.88 (t, 3H, *J* = 7.3 Hz)	1.20 (t, 3H, *J* = 6.2 Hz)		

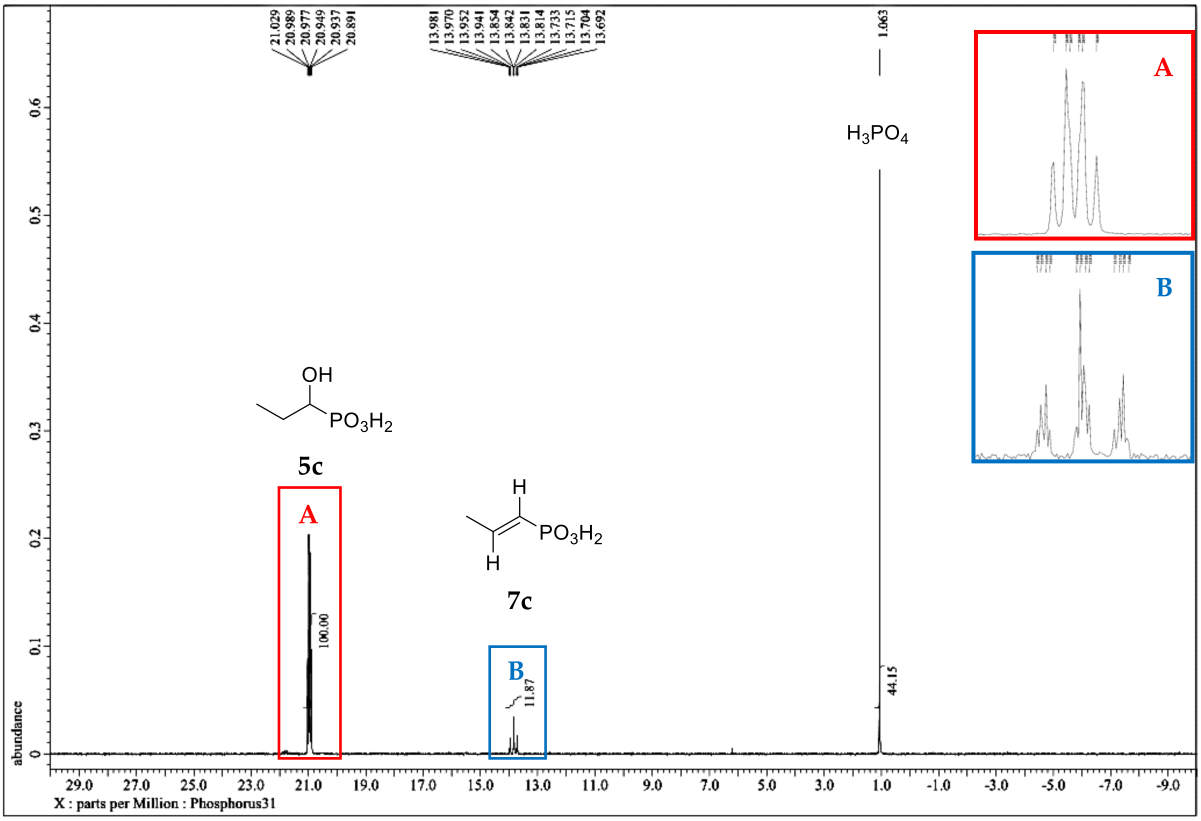

Figure S29. ^{31}P NMR (D_2O, 162MHz) spectra of the crude post-reaction mixture of 1-aminopropylohosphonic acid **(1c)** with $NaNO_2$ after 168h.

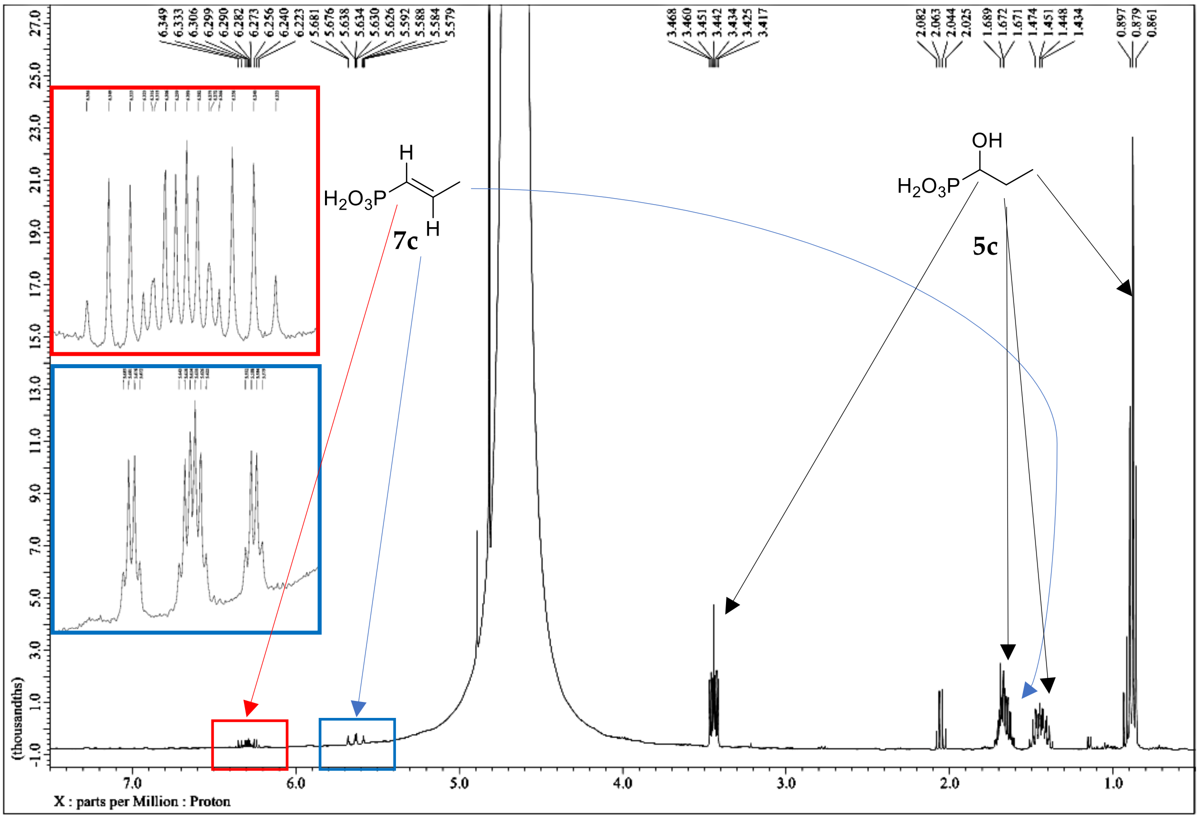

Figure S30. ^{1}H NMR (D_2O, 400MHz) spectra of the crude post-reaction mixture of 1-aminopropylohosphonic acid **(1c)** with $NaNO_2$ after 168h.

*3.7. ABr1214. Deamination of **1i** in Water*

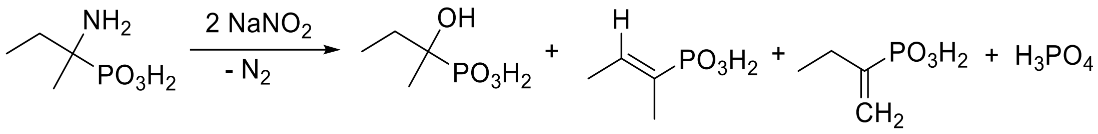

1i **5i** **7i** **7′i**

48h	$\alpha = 0.97$	43%	41%	5%	6%
192 h	$\alpha = 1.00$	43%	42%	8%	7%
δ_P [ppm]	16.82	24.18 (ddq)	17.47 (dqq)	15.63 (ddt)	1.05

After 24 h at 21 °C, post-reaction mixture contained 1-hydroxy-1-methylpropylphosphonic acid **(5i)** (43%molP), *(E)*-1-methylprop-1-en-1-ylphosphonic acid **(7i)** (41%molP), 1-ethylvinylphosphonic acid **(7′i)** (5%molP), orthophosphoric acid (6%molP), unreacted substrate (3%molP) and 2 unidentified compounds (2%molP in total).

After 192 h post-reaction mixture contained 1-hydroxy-1-methylpropylphosphonic acid **(5i)** (43%molP), *(E)*-1-methylprop-1-en-1-ylphosphonic acid **(7i)** (42%molP), 1-ethylvinylphosphonic acid **(7′i)** (8%molP), orthophosphoric acid (7%molP) and trace amounts of 2 unidentified compounds (Figure S31 and Figure S32).

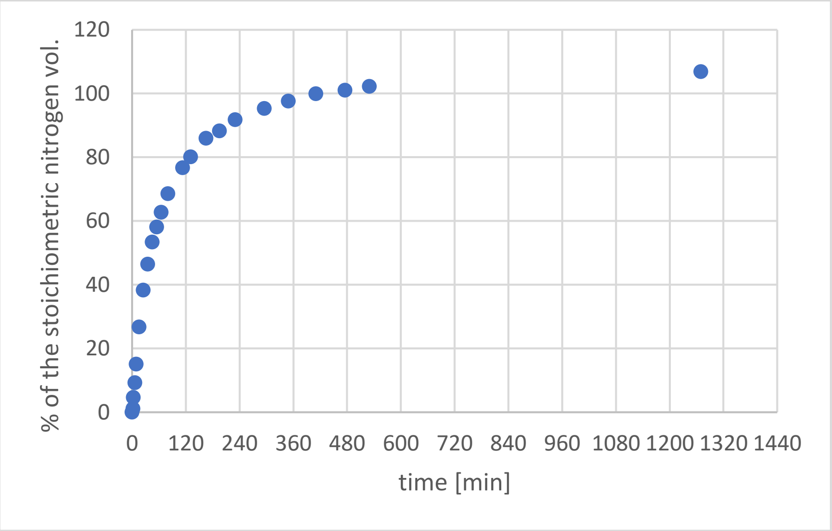

Chart 7. Dependence of the volume of the released gas (in % of the stoichiometric nitrogen volume) on the time in the reaction of 1-amino-1-methylpropylphosphonic acid **(1i)** with $NaNO_2$ in water.

(E)-1-Methylprop-1-en-1-ylphosphonic acid **(7i)** was identified by comparing chemical shifts, multiplecity and coupling constants on ^{1}H and ^{31}P NMR spectra with description in the literature of structurally similar *(E)*-2-(N-hydroxylamino)-1-methyl-pent-1-en-1-ylphosphonic acid (Table S8).

Spectra of 1-hydroxy-1-methylpropylphosphonic acid **(5i)** and 1-ethylvinylphosphonic acid **(7′i)** have been not described in the literature. Therefore these compounds were identified by identified by comparing chemical shifts, multiplecity and coupling constants on ^{1}H and ^{31}P NMR spectra with description in the literature with spectra of structurally simiar 1-hydroxy-1-methylethylphosphonic acid **(5m)** and 1-methylvinylphosphonic acid **(7m)** (experiment ABr1204) (Table S9).

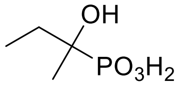

1-Hydroxy-1-methylpropylphosphonic acid (5i). ^{31}P NMR (D_2O): δ 24.18 (ddq, $^3J_{H\text{-}P}$ = 14.5 Hz, $^3J_{H\text{-}P}$ = 11.1 Hz, $^3J_{H\text{-}P}$ = 7.0 Hz); ^{1}H NMR (D_2O) δ: 1.49-1.70 (m, 2H, *J* undetermined), 1.17 (d, 3H, $^3J_{H\text{-}P}$ = 14.4 Hz), 0.81 (t, 3H, $^3J_{H\text{-}H}$ = 7.3 Hz).

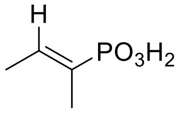

***(E)*-1-Methylprop-1-en-1-ylphosphonic acid (7i).** ^{31}P NMR (D_2O): δ 17.47 (dqq, $^3J_{H\text{-}P(cis)}$ = 21.7 Hz, $^3J_{H\text{-}P}$ = 13.6 Hz, $^4J_{H\text{-}P}$ = 3.7 Hz); ^{1}H NMR (D_2O) δ: 6.15 (ddq, 1H, $^3J_{H\text{-}P}$ = 21.7 Hz, $^3J_{H\text{-}H}$ = 6.7 Hz, $^4J_{H\text{-}H}$ = 1.5 Hz), 1.63 (ddq, 3H, $^3J_{H\text{-}P}$ = 13.6 Hz, $^4J_{H\text{-}H}$ = 1.2 Hz, $^5J_{H\text{-}H}$ =1.2 Hz), 1.57 (ddq, 3H, $^3J_{H\text{-}H}$ = 6.7 Hz, $^4J_{H\text{-}P}$ = 3.7 Hz, $^5J_{H\text{-}H}$ = 1.2 Hz).

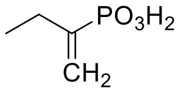

1-Ethylvinylphosphonic acid (7'i). ^{31}P NMR (D_2O): δ 15.63 (ddt, $^3J_{H\text{-}P\,(trans)}$ = 44.4 Hz, $^3J_{H\text{-}P\,(cis)}$ = 21.5 Hz, $^3J_{H\text{-}P}$ = 10.3 Hz); ^{1}H NMR (D_2O) δ: 5.56 (ddt, 1H, $^3J_{H\text{-}P\,(cis)}$ = 21.4 Hz, $^2J_{H\text{-}H}$ = 1.5 Hz, $^4J_{H\text{-}H}$ = 1.2 Hz), 5.39 (ddt, 1H, $^3J_{H\text{-}P\,(trans)}$ = 44.3 Hz, $^4J_{H\text{-}H}$ = 1.8 Hz, $^2J_{H\text{-}H}$ = 1.5 Hz), 2.12 (dddq, 2H, $^3J_{H\text{-}P}$ = 10.3 Hz, $^3J_{H\text{-}H}$ = 7.3 Hz, $^4J_{H\text{-}H}$ = 1.8 Hz, $^4J_{H\text{-}H}$ = 1.2 Hz), 0.95 (t, 3H, $^3J_{H\text{-}H}$ = 7.3 Hz).

Table S8. Comparison of chemical shifts and coupling constants on ^{1}H and ^{31}P NMR spectra of *(E)*-1-methylprop-1-en-1-ylphosphonic acid **(7i)** with spectra of structurally similar *(E)*-2-(N-hydroxylamino)-1-methyl-pent-1-en-1-ylphosphonic acid.

**Structure**	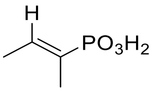	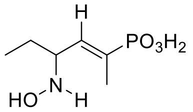
**Source**	**ABr1214**	**Reference [14]**
**Solvent**	**D_2O**	**D_2O**
**^{31}P NMR**	17.47 (dqq, J = 21.7, 13.6, 3.7 Hz)	n. d.
**^{1}H NMR**	6.15 (ddq, 1H, J = 21.7, 6.7, 1.5 Hz)	6.12 (dd, 1H, *J* = 9.8, 21.2 Hz)
		4.35 (m, 1H)
		2.05 (m, 2H)
	1.63 (ddq, 3H, *J* = 13.6, 1.2, 1.2 Hz)	2.02 (d, 3H, *J* = 13.8 Hz)
	1.57 (ddq, 3H, J = 6.7, 3.7, 1.2 Hz)	
		1.04 (t, 3H, *J* = 7.4 Hz)

Table S9. Comparison of chemical shifts and coupling constants on ^{1}H and ^{31}P NMR spectra of the reaction products of 1-amino-1-methylpropylphosphonic acid **(1i)** with $NaNO_2$ with spectra of 1-hydroxy-1-methylethylphosphonic acid **(5m)** and 1-methylvinylphosphonic acid **(7′m).**

**Structure**	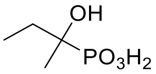	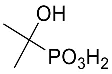	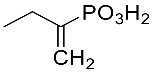	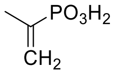
**Source**	**ABr1214**	**ABr1204**	**ABr1214**	**ABr1204**
**Solvent**	D_2O	D_2O	D_2O	D_2O
**^{31}P NMR**	24.18 (ddq, *J* = 14.5, 11.1, 7.0 Hz)	23.84 (septet, *J* = 14.0 Hz)	15.63 (ddt, *J* = 44.4, 21.5, 10.3 Hz)	15.21 (ddq, *J* = 43.9, 20.6, 13.1 Hz)
**^{1}H NMR**	1.49-1.70 (m, 2H, *J* undetermined)		5.56 (ddt, 1H, *J* = 21.4, 1.5, 1.2 Hz)	5.46 (ddq, 1H, *J* = 20.3, 2.1, 1.2 Hz)
	1.17 (d, 3H, *J* = 14.4 Hz)	1.23 (d, 6H, *J* = 13.8 Hz)	5.39 (ddt, 1H, *J* = 44.3, 1.8, 1.5 Hz)	5.36 (tq, 1H, *J* = 43.4, 1.8 Hz)
	0.81 (t, 3H, *J* = 7.3 Hz)		2.12 (dddq, 2H, *J* = 10.3, 7.3, 1.8, 1.2 Hz)	1.77 (dt, 3H, *J* = 13.1, 1.2 Hz)
			0.95 (t, 3H, *J* = 7.3 Hz).	

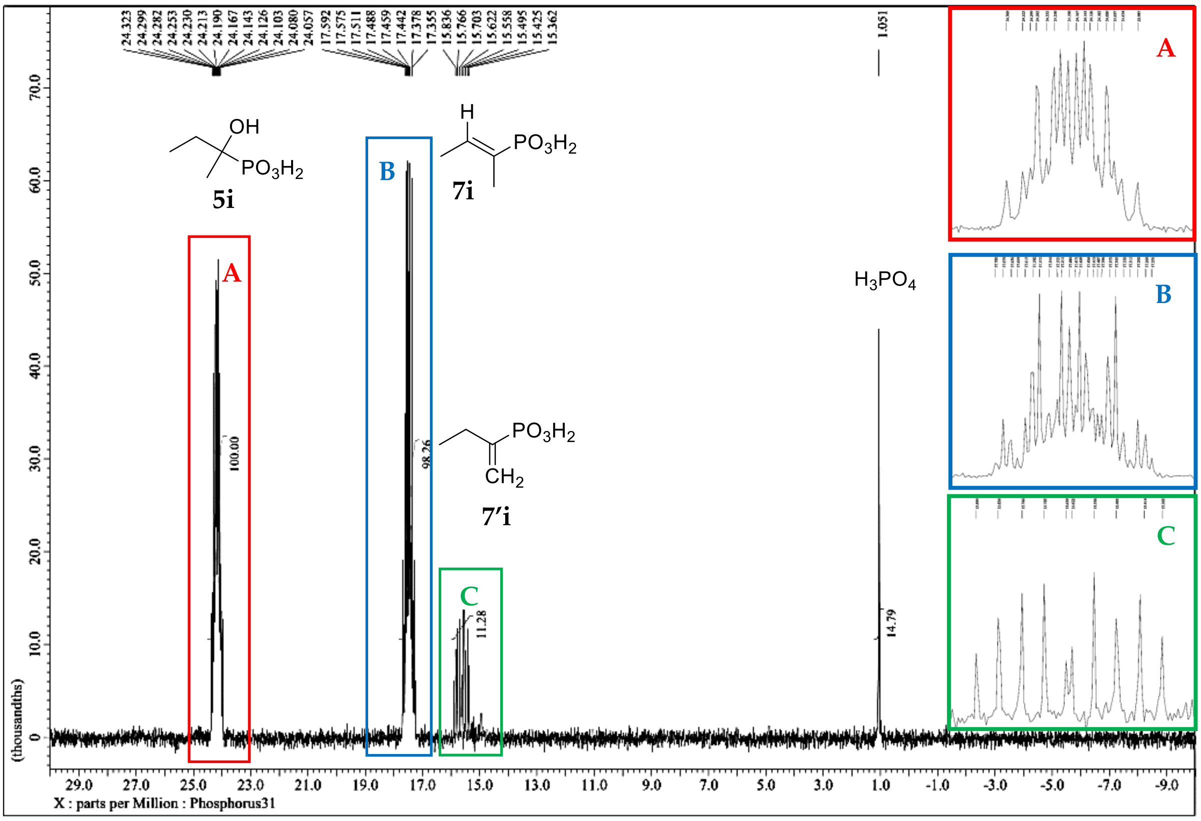

Figure S31. ^{31}P NMR (D_2O, 162MHz) spectra of the crude post-reaction mixture of 1-amino-1-methylpropylphosphonic acid **(1i)** with $NaNO_2$ after 192 h.

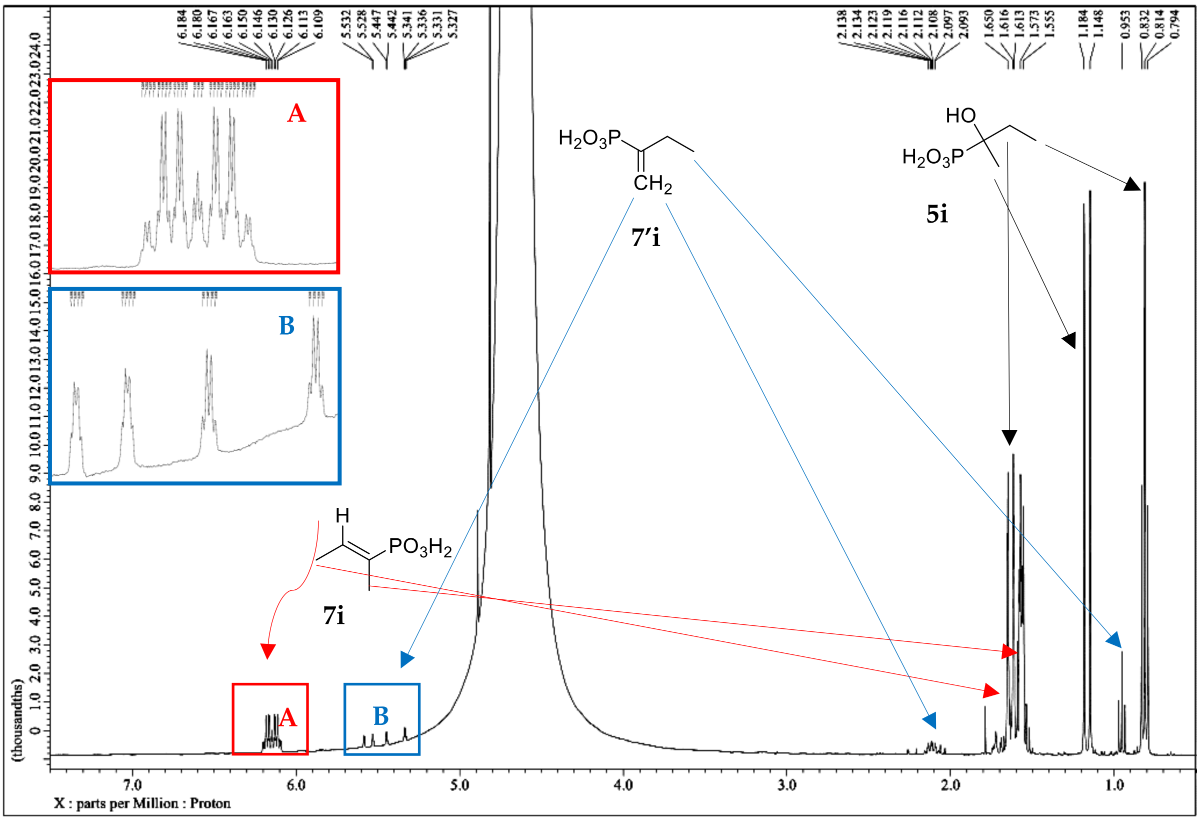

Figure S32. ^{1}H NMR (D_2O, 400MHz) spectra of the crude post-reaction mixture of 1-amino-1-methylpropylphosphonic acid **(1i)** with $NaNO_2$ after 192 h.

3.8. ABr1216. Deamination of ***1l*** *in Water*

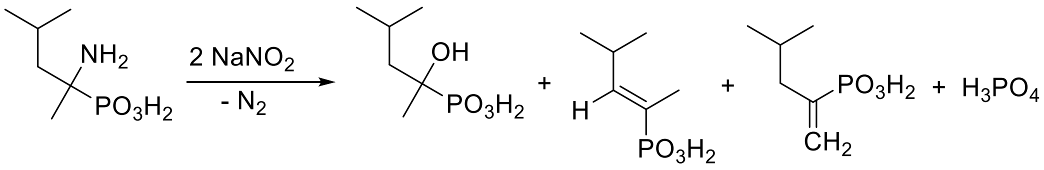

1l **5l** **7l** **7′l**

24 h	α = 0.97	29%	57%	7%	3%
192 h	α = 1.00	30%	59%	7%	4%
δ_P [ppm]	16.82	24.18 (tq)	17.87 (ddq)	15.6 (ddt)	1.09

After 24 h at 21 °C, post-reaction mixture contained 1-hydroxy-1,3-dimethylbutylphosphonic acid **(5l)** (29%molP), *(E)*-1,3-dimethylbut-1-en-1-ylphosphonic acid **(7l)** (57%molP), 4-methylpent-1-en-2-ylphosphonic acid **(7′l)** (7%molP), orthophosphoric acid (3%molP), unreacted substrate **(1l)** (3%molP) and unidentified compound (ca. 1%molP).

After 192 h post-reaction mixture contained 1-hydroxy-1,3-dimethylbutylphosphonic acid **(5l)** (30%molP), *(E)*-1,3-dimethylbut-1-en-1-ylphosphonic acid **(7l)** (59%molP), 4-methylpent-1-en-2-ylphosphonic acid **(7′l)** (7%molP), orthophosphoric acid (4%molP) and trace amounts of unidentified compound (>1%molP) (Figure S33 and Figure S34).

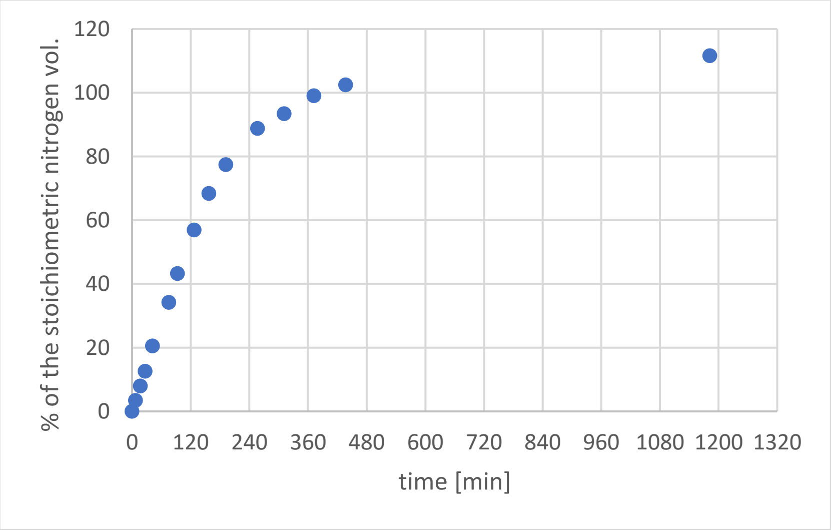

Chart 8. Dependence of the volume of the released gas (in % of the stoichiometric nitrogen volume) on the time in the reaction of 1-amino-1,3-dimethylbutylphosphonic acid **(1l)** with $NaNO_2$ in water.

Spectra of *(E)*-1,3-dimethylbut-1-en-1-ylphosphonic acid **(7l)** have been not described in the literature. Therefore, this compound was identified by comparing chemical shifts, multiplecity and coupling constants on ^1H and ^{31}P NMR spectra with spectra of *(E)*-1-methylprop-1-en-1-ylphosphonic acid **(7i)**, which was obtained in the reaction of 1-amino-1-methylpropylphosphonic acid **(1i)** with $NaNO_2$ (ABr1214) (Table S10).

Spectra of 1-hydroxy-1,3-dimethylbutylphosphonic acid **(5l)** and 4-methylpent-1-en-2-ylphosphonic acid **(7′l)** have been not described in the literature. Therefore, these compounds were identified by comparing chemical shifts, multiplecity and coupling constants on ^1H and ^{31}P NMR spectra with spectra of 1-hydroxy-1-methylethylphosphonic

acid **(5m)** and 1-methylvinylphosphonic acid **(7m)**, which were obtained in the reaction of 1-amino-1-methylethylphosphonic acid **(1m)** with $NaNO_2$ (ABr1204) (Table S11).

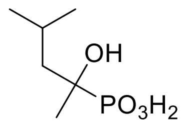

1-Hydroxy-1,3-dimethylbutylphosphonic acid (5l). ^{31}P NMR (D_2O): δ 24.18 (tq, $^3J_{H\text{-}P}$ = 15.0 Hz, $^3J_{H\text{-}P}$ = 7.5 Hz); ^{1}H NMR (D_2O) δ: 1.78 (doublet of septets, 1H, $^3J_{H\text{-}H}$ = 7.0 Hz, $^3J_{H\text{-}H}$ = 6.4 Hz), 1.43-1.55 (m, 2H), 1.23 (d, 3H, $^3J_{H\text{-}P}$ = 15.0 Hz), 0.81 (d, 3H, $^3J_{H\text{-}H}$ = 6.4 Hz), 0.79 (d, 3H, $^3J_{H\text{-}H}$ = 6.1 Hz).

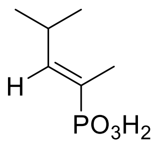

***(E)*-1,3-Dimethylbut-1-en-1-ylphosphonic acid (7l).** ^{31}P NMR (D_2O): δ 17.87 (ddq, $^3J_{H\text{-}P}$ = 22.4 Hz, $^3J_{H\text{-}P}$ = 14.0 Hz, $^4J_{H\text{-}P}$ = 2.8 Hz); ^{1}H NMR (D_2O) δ: 5.88 (ddq, 1H, $^3J_{H\text{-}P}$ = 22.2 Hz, $^3J_{H\text{-}H}$ = 9.3 Hz, $^4J_{H\text{-}H}$ = 1.8 Hz), 2.51 (doublet of doublets of septets, 1H, $^3J_{H\text{-}H}$ = 9.3 Hz, $^3J_{H\text{-}H}$ = 6.6 Hz, $^3J_{H\text{-}H}$ = 2.8 Hz), 1.65 (dd, 3H, $^3J_{H\text{-}P}$ = 13.9 Hz, $^4J_{H\text{-}H}$ = 1.8 Hz), 0.83 (d, 6H, $^3J_{H\text{-}H}$ = 6.7 Hz).

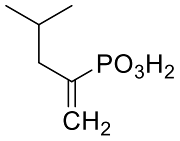

4-Methylpent-1-en-2-ylphosphonic acid (7′l). ^{31}P NMR (D_2O): δ 15.6 (ddt, $^3J_{H\text{-}P\,(trans)}$ = 44.1 Hz, $^3J_{H\text{-}P\,(cis)}$ = 21.0 Hz, $^3J_{H\text{-}P}$ = 14.2 Hz); ^{1}H NMR (D_2O) δ: 5.61 (ddd, 1H, $^3J_{H\text{-}P\,(cis)}$ = 21.1 Hz, $^4J_{H\text{-}H}$ = 2.1 Hz, *J* undetermined), 5.37 (ddd, 1H, $^3J_{H\text{-}P\,(trans)}$ = 44.5 Hz, $^4J_{H\text{-}H}$ = 3.4 Hz, $^4J_{H\text{-}H}$ = 1.5 Hz), 1.98 (br dddd, 2H, $^3J_{H\text{-}P}$ = 14.1 Hz, $^3J_{H\text{-}H}$ = 7.0 Hz, J nieozn), 1.70-1.85 (m, 1H, *J* undetermined), 0.72 (d, 6H, $^3J_{H\text{-}H}$ = 6.7 Hz).

Table S10. Comparison of chemical shifts and coupling constants on ^{1}H and ^{31}P NMR spectra of *(E)*-1,3-7ldimethylbut-1-en-1-ylphosphonic acid **(7l)** with *(E)*-1-methylprop-1-en-1-ylphosphonic acid **(7i)** (ABr1214).

**Structure**	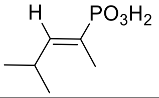	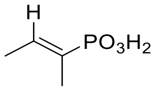
**Source**	**ABr1216**	**ABr1214**
**Solvent**	**D_2O**	**D_2O**
**^{31}P NMR**	17.87 (ddq, *J* = 22.4, 14.0, 2.8 Hz)	17.47 (dqq, *J* = 21.7, 13.6, 3.7 Hz)
**^{1}H NMR**	5.88 (ddq, 1H, *J* = 22.2, 9.3, 1.8 Hz),	6.15 (ddq, 1H, *J* = 21.7, 6.7, 1.5 Hz)
	2.51 (doublet of doublets of septets, 1H, *J* = 9.3, 6.6, 2.8 Hz)	
	1.65 (dd, 3H, *J* = 13.9, 1.8 Hz)	1.63 (ddq, 3H, *J* = 13.6, 1.2, 1.2 Hz)
	0.83 (d, 6H, *J* = 6.7 Hz)	
		1.57 (ddq, 3H, J = 6.7, 3.7, 1.2 Hz)

Table S11. Comparison of chemical shifts and coupling constants on ^{1}H and ^{31}P NMR spectra of post-reaction mixture of 1-hydroxy-1,3-dimethylbutylphosphonic acid **(1l)** with $NaNO_2$ with 1-hydroxy-1-methylethylphosphonic acid **(5m)** and 1-methylvinylphosphonic acid **(7m)** (ABr1204).

**Structure**	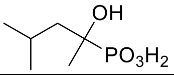	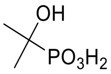	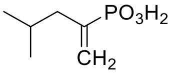	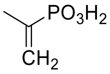
**Source**	**ABr1216**	**ABr1204**	**ABr1216**	**ABr1204**
**Solvent**	**D_2O**	**D_2O**	**D_2O**	**D_2O**
**^{31}P NMR**	24.18 (tq, *J* = 15.0, 7.5 Hz)	23.84 (septet, *J* = 14.0 Hz)	15.6 (ddt, *J* = 44.1, 21.0, 14.2 Hz);	15.21 (ddq, *J* = 43.9, 20.6, 13.1 Hz)
**^{1}H NMR**	1.78 (doublet of septets, 1H, *J* = 7.0, 6.4 Hz),		5.61 (ddd, 1H, *J* = 21.1, 2.1 Hz, *J* undetermined)	5.46 (ddq, 1H, *J* = 20.3, 2.1, 1.2 Hz)
	1.43-1.55 (m, 2H),		5.37 (ddd, 1H, *J* = 44.5, 3.4, 1.5 Hz)	5.36 (tq, 1H, *J* = 43.4, 1.8 Hz)
	1.23 (d, 3H, *J* = 15.0 Hz)	1.23 (d, 6H, *J* = 13.8 Hz)	1.98 (br dddd, 2H, *J* = 14.1, 7.0 Hz)	1.77 (dt, 3H, *J* = 13.1, 1.2 Hz)
	0.81 (d, 3H, *J* = 6.4 Hz)		1.70-1.85 (m, 1H, J undetermined)	
	0.79 (d, 3H, *J* = 6.1 Hz)		0.72 (d, 6H, *J* = 6.7 Hz)	

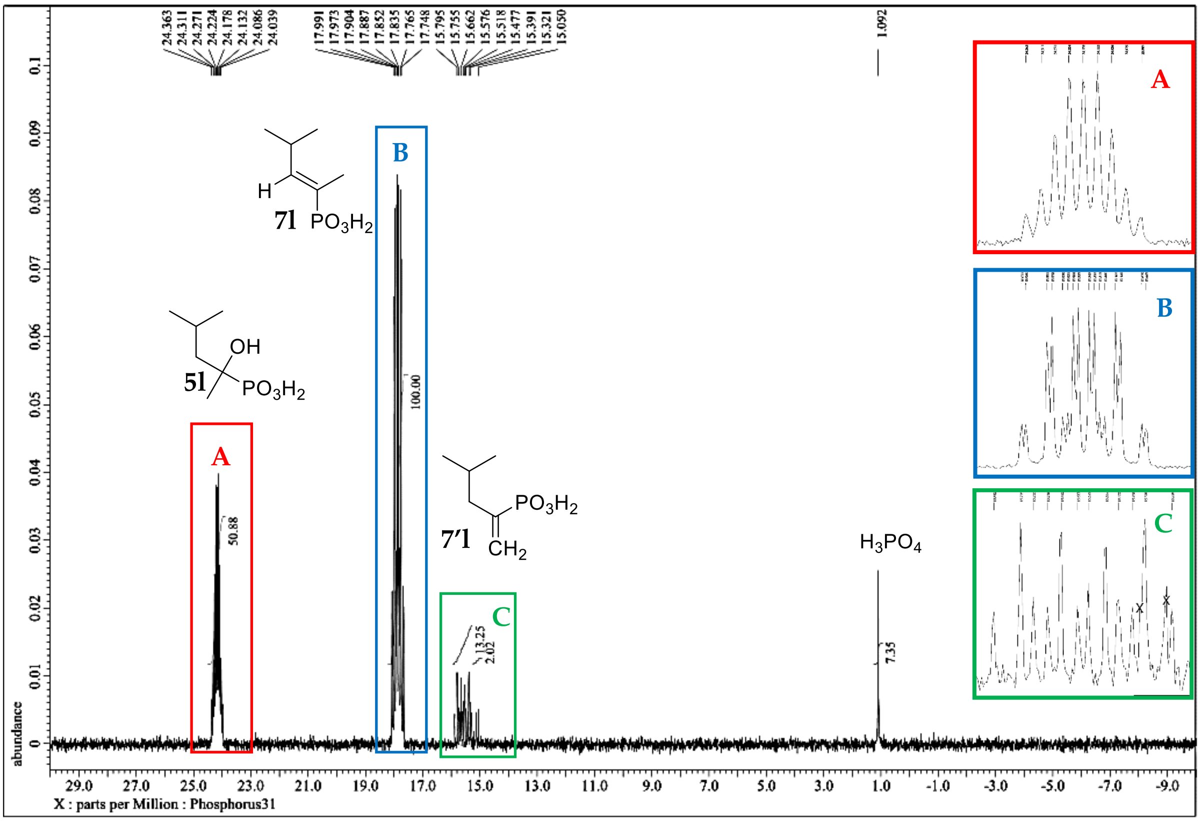

Figure S33. ^{31}P NMR (D_2O, 162MHz) spectra of the crude post-reaction mixture of 1-amino-1,3-dimethylbutylphosphonic acid **(1l)** with $NaNO_2$ after 192 h.

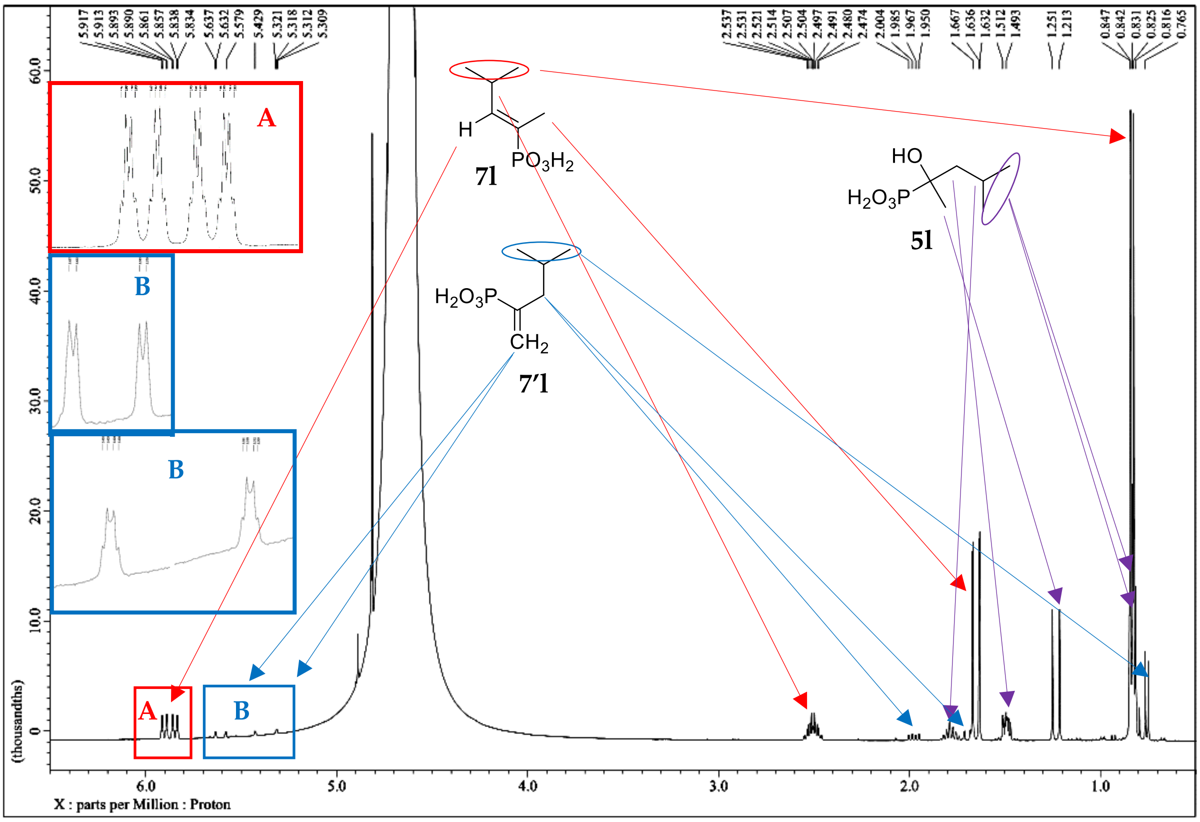

Figure S34. ^{1}H NMR (D_2O, 400MHz) spectra of the crude post-reaction mixture of 1-amino-1,3-dimethylbutylphosphonic acid **(1l)** with $NaNO_2$ after 192 h.

3.9. ABr1218. Deamination of ***1k*** *in Water*

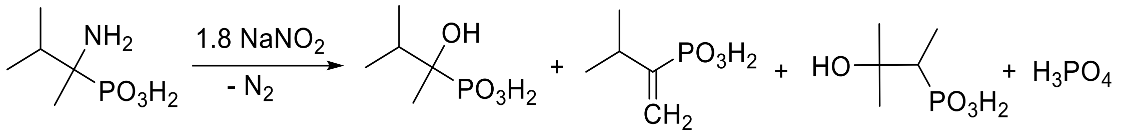

1k **5k** **7k** **5′k**

**96h**	**α = 0.97**	**39%**	**8%**	**6%**	**39%**
168h	α = 1.00	40%	8%	6%	40%
δ_P [ppm]	16.82	24.06 (dq)	15.84 (ddd)	26.36 (dq)	1.13

After 96h at 21 °C, post-reaction mixture contained 1-hydroxy-1,2-dimethylpropylphosphonic acid **(5k)** (39%molP), 3-methylbut-1-en-2-ylphosphonic acid **(7k)** (8%molP), 2-hydroxy-2,3-dimethylpropylphosphonic acid **(5′k)** (6%molP), orthophosphoric acid (39%molP) and 2 unidentified compounds (8%molP in total).

After 168h post-reaction mixture contained 1-hydroxy-1,2-dimethylpropylphosphonic acid **(5k)** (40%molP), 3-methylbut-1-en-2-ylphosphonic acid **(7k)** (8%molP), 2-hydroxy-2,3-dimethylpropylphosphonic acid **(5′k)** (6%molP), orthophosphoric acid (40%molP) and 2 unidentified compounds (6%molP in total) (Figure S35 and Figure S36).

^{31}P and ^{1}H NMR:

1-Hydroxy-1,2-dimethylpropylphosphonic acid (5k). ^{31}P NMR (D_2O): δ 24.06 (dq, $^3J_{H\text{-}P}$ = 14.0 Hz, $^3J_{H\text{-}P}$ = 8.4 Hz); ^{1}H NMR (D_2O) δ: 1.91 (dqq, 1H, $^3J_{H\text{-}P}$ = 8.3 Hz, $^3J_{H\text{-}H}$ = 7.0 Hz, $^3J_{H\text{-}H}$ = 6.7 Hz), 1.10 (d, 3H, $^3J_{H\text{-}P}$ = 14.4 Hz), 0.86 (d, 3H, $^3J_{H\text{-}H}$ = 7.0 Hz), 0.83 (d, 3H, $^3J_{H\text{-}H}$ = 6.7 Hz).

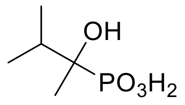

3-Methylbut-1-en-2-ylphosphonic acid (7k). ^{31}P NMR (D_2O): δ 15.84 (ddd, $^3J_{H\text{-}P(trans)}$ = 45.1 Hz, $^3J_{H\text{-}P(cis)}$ = 22.0 Hz, $^3J_{H\text{-}P}$ = 12.2 Hz); ^{1}H NMR (D_2O) δ: 5.59 (ddd, 1H, $^3J_{H\text{-}P\,(trans)}$ = 45.2 Hz, $^3J_{H\text{-}H}$ = 1.5 Hz, $^4J_{H\text{-}H}$ = 1.2 Hz), 5.45 (ddd, 1H, $^3J_{H\text{-}P(cis)}$ = 22.0 Hz, $^3J_{H\text{-}H}$ = 1.2 Hz, *J* undetermined), 2.46 (br septet, 1H, $^3J_{H\text{-}H}$ = 6.7 Hz), 0.96 (d, 6H, $^3J_{H\text{-}H}$ = 6.7 Hz).

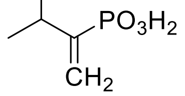

2-Hydroxy-2,3-dimethylpropylphosphonic acid (5′k). ^{31}P NMR (D_2O): δ 26.36 (dq, $^2J_{H\text{-}P}$ = 17.8 Hz, $^3J_{H\text{-}P}$ = 16.8 Hz); ^{1}H NMR (D_2O) δ: 1.74 (dq, 1H, $^2J_{H\text{-}P}$ = 18.3 Hz, $^3J_{H\text{-}H}$ = 7.6 Hz), 1.19 (s, 3H), 1.11 (s, 3H), 1.00 (dd, 3H, $^3J_{H\text{-}P}$ = 16.7 Hz, $^3J_{H\text{-}H}$ = 7.6 Hz).

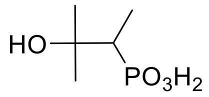

1-Hydroxy-1,2-dimethylpropylphosphonic acid **(5k)** and 3-methylbut-1-en-2-ylphosphonic acid **(7k)** were identified by comparing chemical shifts, multiplecity and coupling constants on ^{1}H and ^{31}P NMR spectra with z description of structurally similar 1-hydroxy-1-methylethylphosphonic acid **(5m)** and 1-methylvinylphosphonic acid **(7m)**, which were obtained in the reaction of 1-amino-1-methylethylphosphonic acid **(1m)** with $NaNO_2$ (ABr1204) (Table S12).

2-hydroxy-2,3-dimethylpropylphosphonic acid **(5′k)** was identified by comparing chemical shifts, multiplecity and coupling constants on ^{1}H and ^{31}P NMR spectra with z description of structurally similar 2-hydroxypropylphosphonic acid (Table S13).

Table S12. Comparison of chemical shifts and coupling constants on ^{1}H and ^{31}P NMR spectra of the reaction products of 1-amino-1,2-dimethylpropylphosphonic acid **(1k)** z $NaNO_2$ with spectra of similar 1-hydroxy-1-methylethylphosphonic acid **(5m)** and 1-methylvinylphosphonic acid **(7m)** (ABr1204).

**Structure**	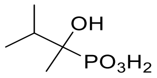	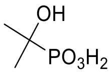	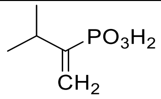	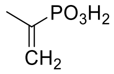
**Source**	**ABr1218**	**ABr1204**	**ABr1218**	**ABr1204**
**Solvent**	**D_2O**	**D_2O**	**D_2O**	**D_2O**
**^{31}P NMR**	24.06 (dq, *J* = 14.0, 8.4 Hz)	23.84 (septet, *J* = 14.0 Hz)	15.84 (ddd, *J* = 45.1, 22.0, 12.2 Hz)	15.21 (ddq, *J* = 43.9, 20.6, 13.1 Hz)
**^{1}H NMR**	1.91 (dqq, 1H, *J* = 8.3, 7.0, 6.7 Hz)		5.59 (ddd, 1H, *J* = 45.2, 1.5, 1.2 Hz)	5.46 (ddq, 1H, *J* = 20.3, 2.1, 1.2 Hz)
	1.10 (d, 3H, *J* = 14.4 Hz)	1.23 (d, 6H, *J* = 13.8 Hz)	5.45 (ddd, 1H, *J* = 22.0, 1.2 Hz, *J* undetermined),	5.36 (tq, 1H, *J* = 43.4, 1.8 Hz)
	0.86 (d, 3H, *J* = 7.0 Hz)		2.46 (br septet, 1H, *J* = 6.7 Hz)	1.77 (dt, 3H, *J* = 13.1, 1.2 Hz)
	0.83 (d, 3H, *J* = 6.7 Hz)		0.96 (d, 6H, *J* = 6.7 Hz)	

Table S13. Comparison of chemical shifts and coupling constants on ^{1}H and ^{31}P NMR spectra of the reaction products of 1-hydroxy-1,2-dimethylpropylphosphonic acid **(5′k)** with spectra of similar 2-hydroxypropylphosphonic acid.

**Structure**	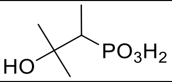	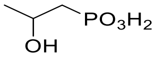
**Source**	**ABr1218**	**Reference [9]**
**Solvent**	**D_2O**	**D_2O**
**^{31}P NMR**	26.36 (dq, *J* = 17.8, 16.8 Hz)	19.9
**^{1}H NMR**		3.80 (1H, dm, *J* = 6.5 Hz)
	1.74 (dq, 1H, *J* = 18.3, 7.6 Hz),	1.39 (2H, ddd, *J* = 18.0, 6.6, 15.3 Hz)
	1.19 (s, 3H),	0.97 (3H, d, *J* = 6.5 Hz)
	1.11 (s, 3H),	
	1.00 (dd, 3H, *J* = 16.7, *J* = 7.6 Hz)	

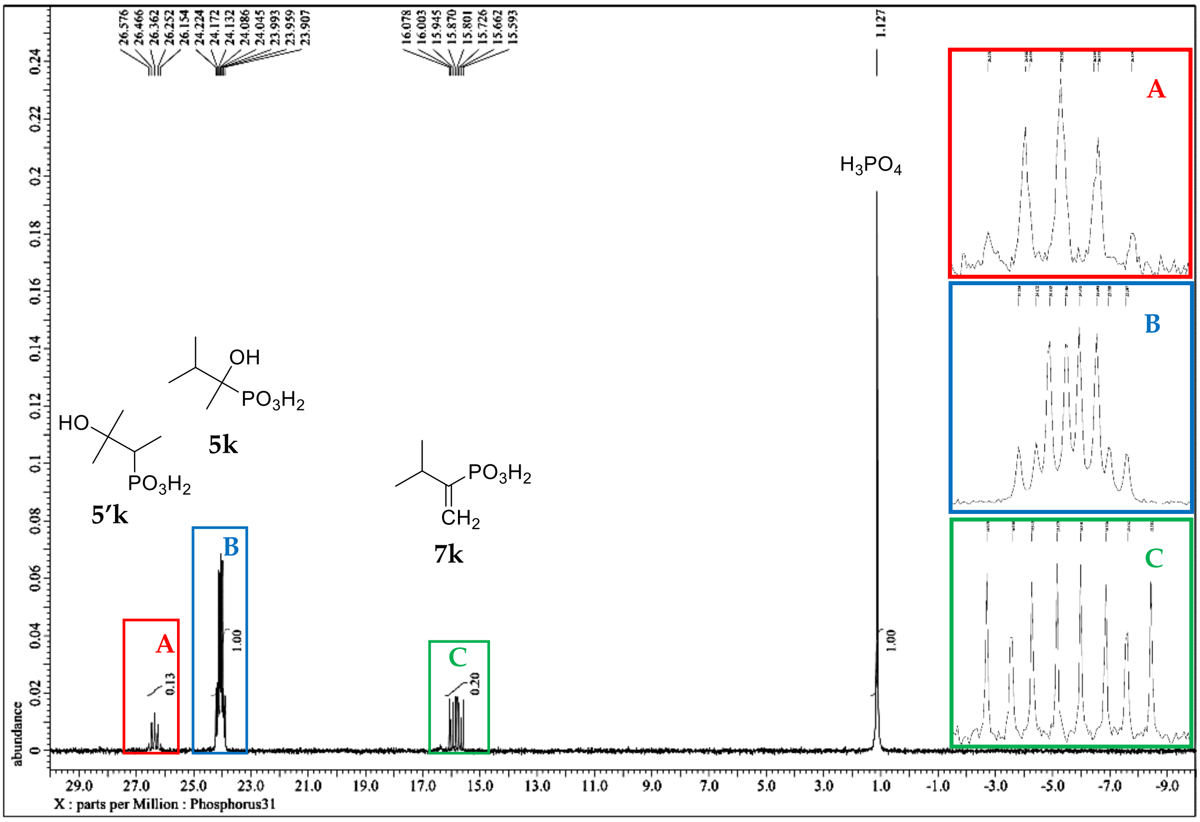

Figure S35. ^{31}P NMR (D_2O, 162MHz) spectra of the crude post-reaction mixture of 1-amino-1,2-dimethylpropylphosphonic acid **(1k)** z $NaNO_2$ after168h.

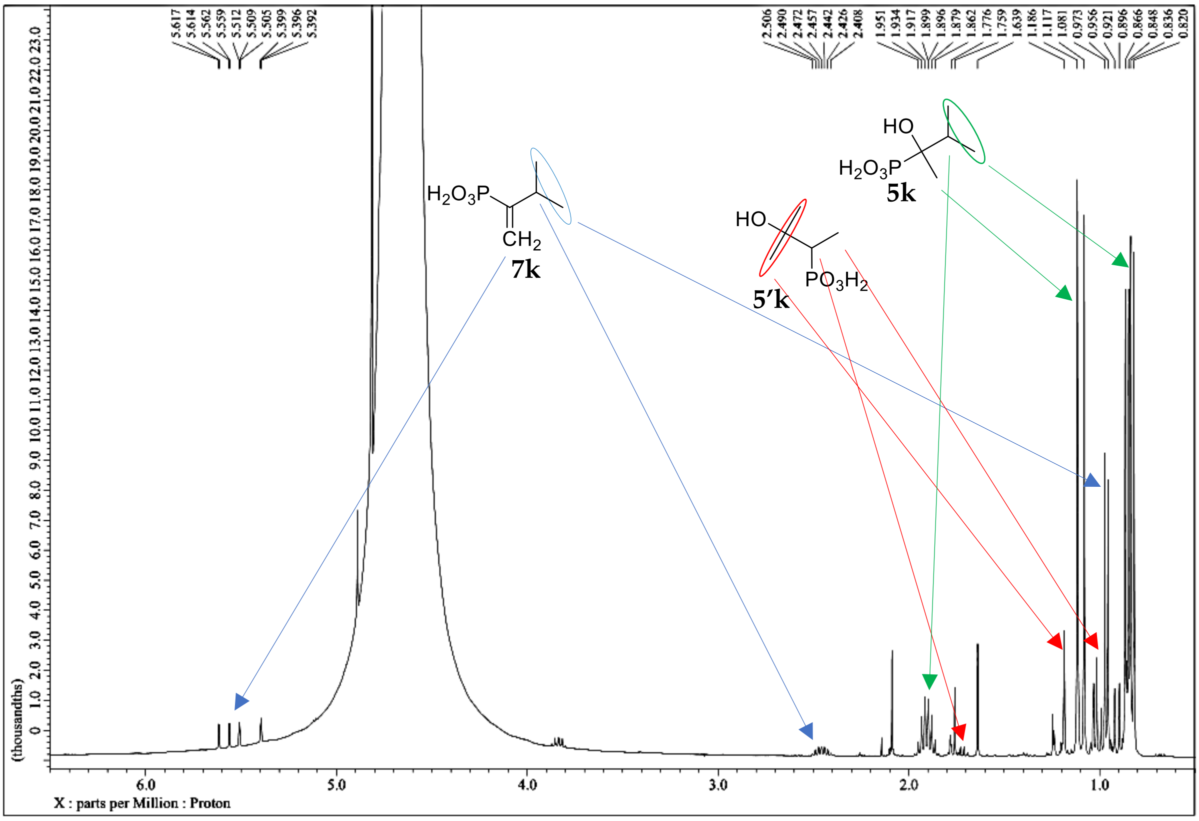

Figure S36. ^{1}H NMR (D_2O, 400MHz) spectra of the crude post-reaction mixture of 1-amino-1,2-dimethylpropylphosphonic acid **(1k)** z $NaNO_2$ after168h.

*3.10. ABr1220. Deamination of **1j** in Water*

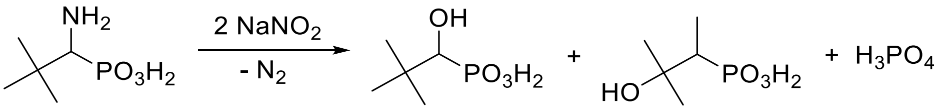

1j **5j** **5′j**

96h	α = 1.00	7%	6%	86%
δ_P [ppm]		19.91 (d)	26.44 (dq)	0.86

After 96h at 21 °C, post-reaction mixture contained orthophosphoric acid (86%molP), 1-hydroxy-2,2-dimethylpropylphosphonic acid **(5j)** (7%molP), 2-hydroxy-1,2-dimethylpropyl-phosphonic acid **(5′j)** (6%molP) and unidentified compound (1%molP) (Figure S37 and Figure S38).

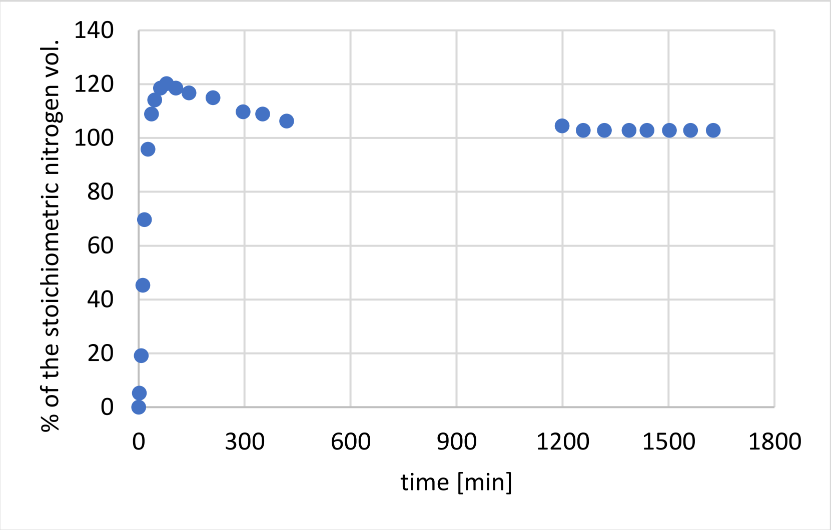

Chart 9. Dependence of the volume of the released gas (in % of the stoichiometric nitrogen volume) on the time in the reaction of 1-amino-2,2-dimethylpropylphosphonic acid **(1j)** with $NaNO_2$ in water.

1-Hydroxy-2,2-dimethylpropylphosphonic acid **(5j)** was identified by comparing chemical shifts, multiplecity and coupling constants on ^{1}H and ^{31}P NMR spectra with description in the literature (Table S14). 2-hydroxy-1,2-dimethylpropylphosphonic acid **(5′j)** was identified by comparing chemical shifts, multiplecity and coupling constants on ^{1}H and ^{31}P NMR spectra with description in the literature with description for structuraally similar 1-hydroxy-2,2-dimethylpropylphosphonic acid **(5k)**, which was obtained in the reaction of 1-amino-1,2-dimethylpropylphosphonic acid **(1k)** with $NaNO_2$ (ABr1218) (Table S14).

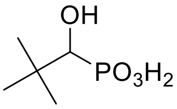

1-Hydroxy-2,2-dimethylpropylphosphonic acid (5j). ^{31}P NMR (D_2O): δ 19.91 (d, $^{2}J_{H-P}$ = 9.4 Hz); ^{1}H NMR (D_2O) δ: 3.29 (d, 1H, $^{2}J_{H-P}$ = 9.8 Hz), 0.90 (s, 9H).

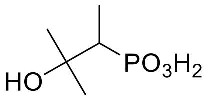

2-Hydroxy-1,2-dimethylpropylphosphonic acid (5′j). ^{31}P NMR (D_2O): δ 26.44 (dq, $^{2}J_{H\text{-}P}$ = 17.8 Hz, $^{3}J_{H\text{-}P}$ = 17.8 Hz); ^{1}H NMR (D_2O) δ: 1.75 (dq, 1H, $^{2}J_{H\text{-}P}$ = 18.8 Hz, $^{3}J_{H\text{-}H}$ = 7.6 Hz), 1.18 (s, 3H), 1.11 (s, 3H), 1.00 (dd, 3H, $^{3}J_{H\text{-}P}$ = 16.8 Hz, $^{3}J_{H\text{-}H}$ = 7.7 Hz).

Table S14. Comparison of chemical shifts and coupling constants on ^{1}H and ^{31}P NMR spectra of post-reaction mixture of 1-amino-2,2-dimethylpropylphosphonic acid **(1j)** z $NaNO_2$ with experimental spectra and description in the literature.

**Structure**	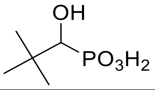		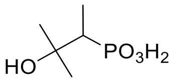	
**Source**	**ABr1220**	**Reference [15]**	**ABr1220**	**ABr1218**
**Solvent**	**D_2O**	**DMSO- d_6**	**D_2O**	**D_2O**
**^{31}P NMR**	19.91 (d, *J* = 9.4 Hz)	n.d.	26.44 (dq, *J* = 17.8 Hz, *J* = 17.8 Hz)	26.36 (dq, *J* = 17.8, 16.8 Hz)
**^{1}H NMR**	3.29 (d, 1H, *J* = 9.8 Hz)	3.24 (d, 1H, *J* = 8.8 Hz)	1.75 (dq, 1H, *J* = 18.8, 7.6 Hz)	1.74 (dq, 1H, *J* = 18.3, 7.6 Hz),
	0.90 (s, 9H)	0.98 (s, 9H)	1.18 (s, 3H)	1.19 (s, 3H),
			1.11 (s, 3H)	1.11 (s, 3H),
			1.00 (dd, 3H, *J* = 16.8, 7.7 Hz).	1.00 (dd, 3H, *J* = 16.7, *J* = 7.6 Hz)

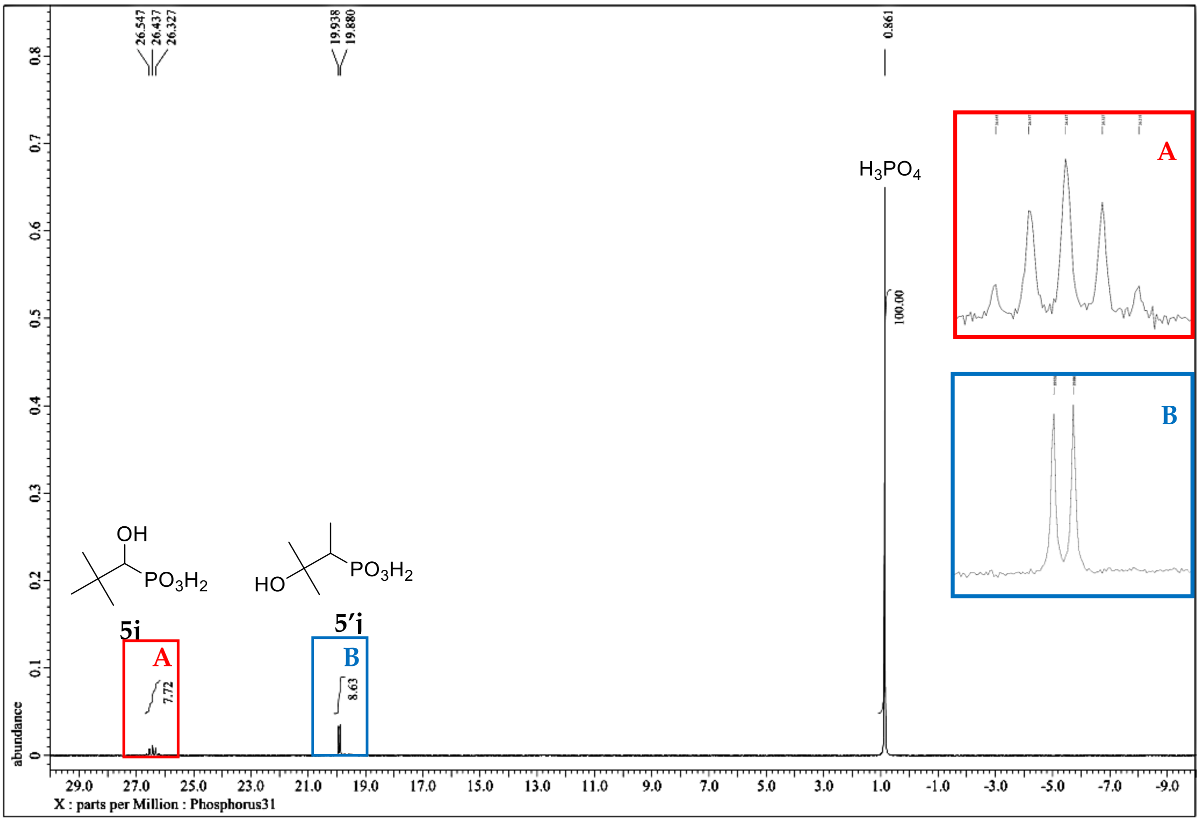

Figure S37. ^{31}P NMR (D_2O, 162MHz) spectra of the crude post-reaction mixture of 1-amino-2,2-dimethylpropylphosphonic acid **(1j)** with $NaNO_2$ after 96h.

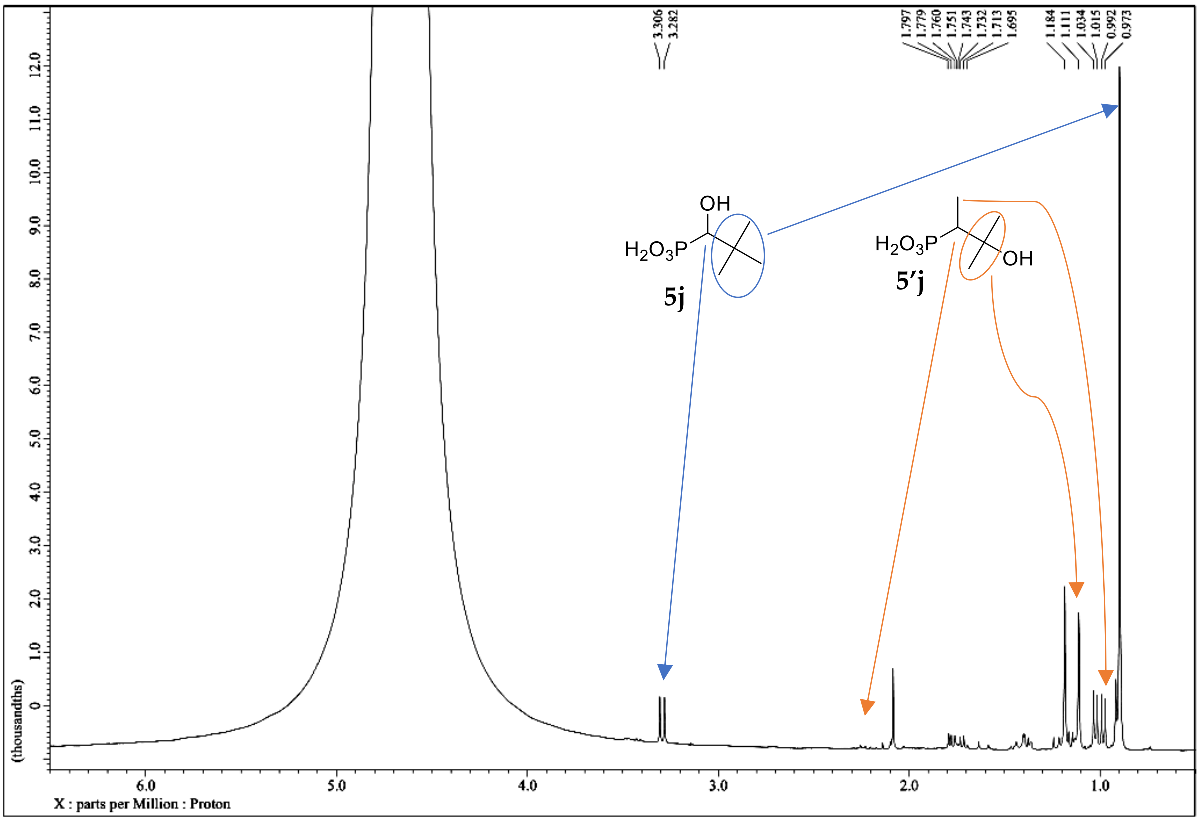

Figure S38. ^{1}H NMR (D_2O, 400MHz) spectra of the crude post-reaction mixture of 1-amino-2,2-dimethylpropylphosphonic acid **(1j)** with $NaNO_2$ after 96h.

3.11. ABr1222. Deamination of ***1q*** *in Water*

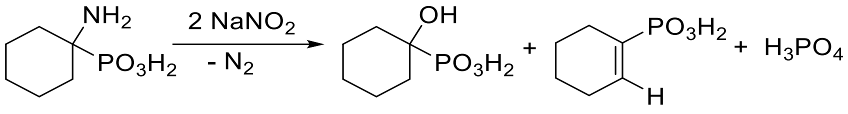

1q **5q** **7q**

98h	$\alpha = 1.00$	16%	75%	7%
δ_P [ppm]		23.88 (br s)	16.25 (doublet of quintets)	1.02

After 98h at 21 °C, post-reaction mixture contained 1-hydroxycyclohexylphosphonic acid **(5q)** (16%molP), cyclohex-1-en-1-ylphosphonic acid **(7q)** (75%molP), orthophosphoric acid (7%molP) and 2 unidentified compounds (2%molP in total) (Figure S39 and Figure S41).

The presence of 1-hydroxycyclohexylphosphonic acid **(5q)** was confirmed by re-measuring the NMR spectra of the reaction mixture with the addition of reference material – 1-hydroxycyclohexylphosphonic acid **(5q) (**Figure S40 and Figure S42).

Cyclohex-1-en-1-ylphosphonic acid **(7q)** was identified by comparing chemical shifts, multiplecity and coupling constants on ^{1}H and ^{31}P NMR spectra with description in the literature (Table S15).

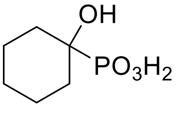

1-Hydroxycyclohexylphosphonic acid (5q). ^{31}P NMR (D_2O): δ 23.88 (br s); ^{1}H NMR (D_2O) δ: 1.60-1.70 (m, 2H), 1.36-1.58 (m, 7H), 1.00-1.12 (m, 1H).

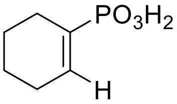

Cyclohex-1-en-1-ylphosphonic acid (7q). ^{31}P NMR (D_2O): δ 16.25 (doublet of quintets, $^{3}J_{H\text{-}P}$ = 20.6 Hz, $^{3}J_{H\text{-}P}$ = 3.7 Hz); ^{1}H NMR (D_2O) δ: 6.28 (dtt, 1H, $^{3}J_{H\text{-}P}$ = 21.1 Hz, $^{3}J_{H\text{-}H}$ = 3.7 Hz, $^{4}J_{H\text{-}H}$ = 1.8 Hz), 1.93-2.04 (m, 4H), 1.38-1.52 (m, 4H).

Table S15. Comparison of chemical shifts and coupling constants on experimental ^{1}H and ^{31}P NMR spectra of cyclohex-1-en-1-ylphosphonic acid **(7q)** with description in the literature.

**Structure**	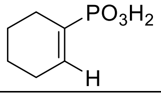	
**Source**	**ABr1222**	**Reference [16]**
**Solvent**	**D_2O**	**D_2O**
**^{31}P NMR**	16.25 (doublet of quintets, J = 20.6, 3.7 Hz)	14.0 (d, J = 19 Hz)
**^{1}H NMR**	6.28 (dtt, 1H, J = 21.1, 3.7, 1.8 Hz)	6.52 (d, J = 19 Hz)
	1.93-2.04 (m, 4H)	
	1.38-1.52 (m, 4H)	

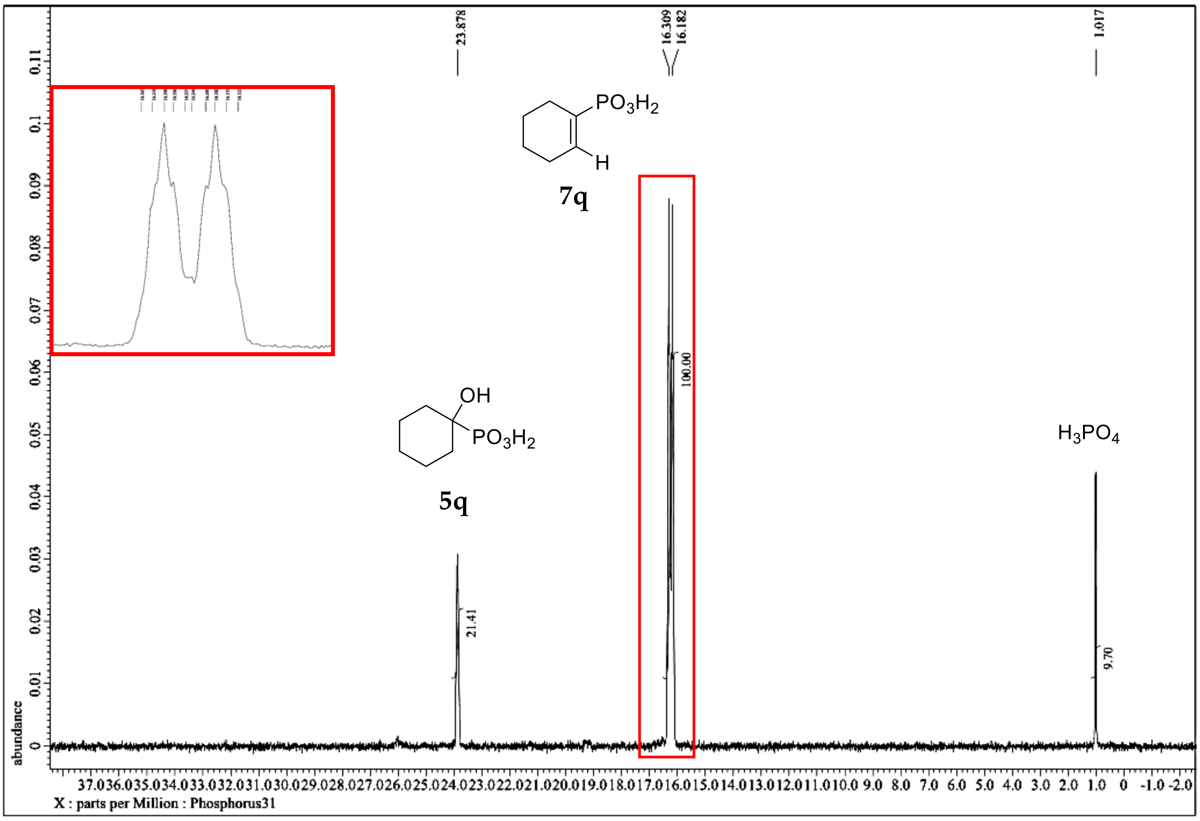

Figure S39. ^{31}P NMR (D_2O, 162MHz) spectra of the crude post-reaction mixture of 1-aminocyclohexylphosphonic acid **(1q)** with $NaNO_2$ after 98h.

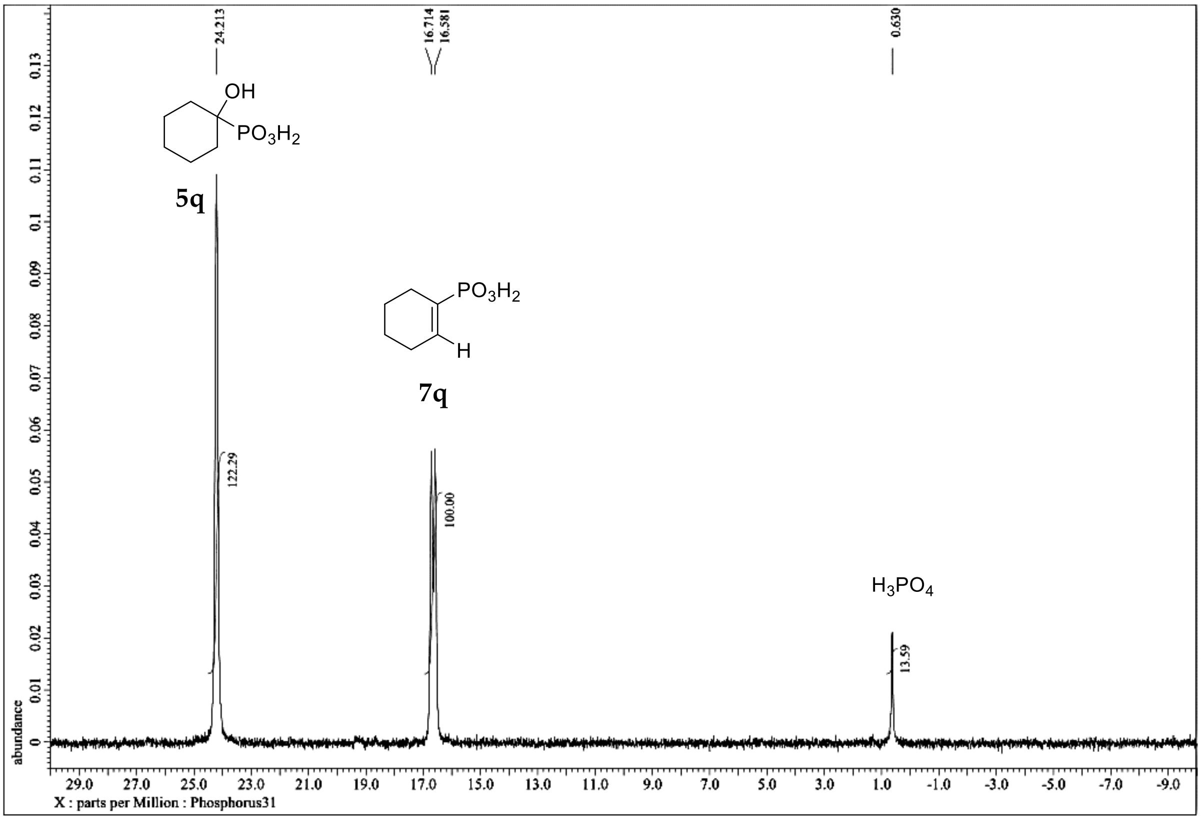

Figure S40. ^{31}P NMR (D_2O, 162MHz) spectra of the crude post-reaction mixture of 1-aminocyclohexylphosphonic acid **(1q)** with $NaNO_2$ after 98h with addition of reference material - 1-hydroxycyclohexylphosphonic acid **(5q)**.

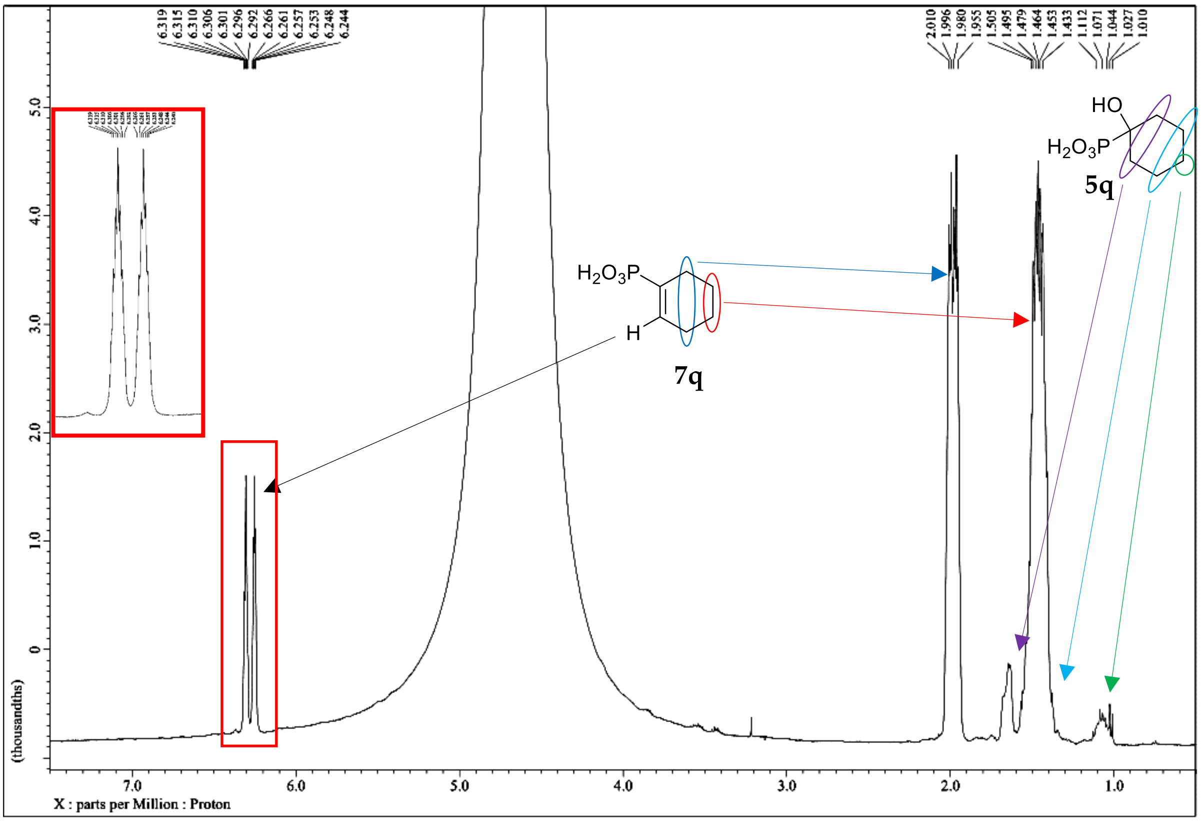

Figure S41. ^{1}H NMR (D_2O, 400MHz) spectra of the crude post-reaction mixture of 1-aminocyclohexylphosphonic acid **(1q)** with $NaNO_2$ after 98h.

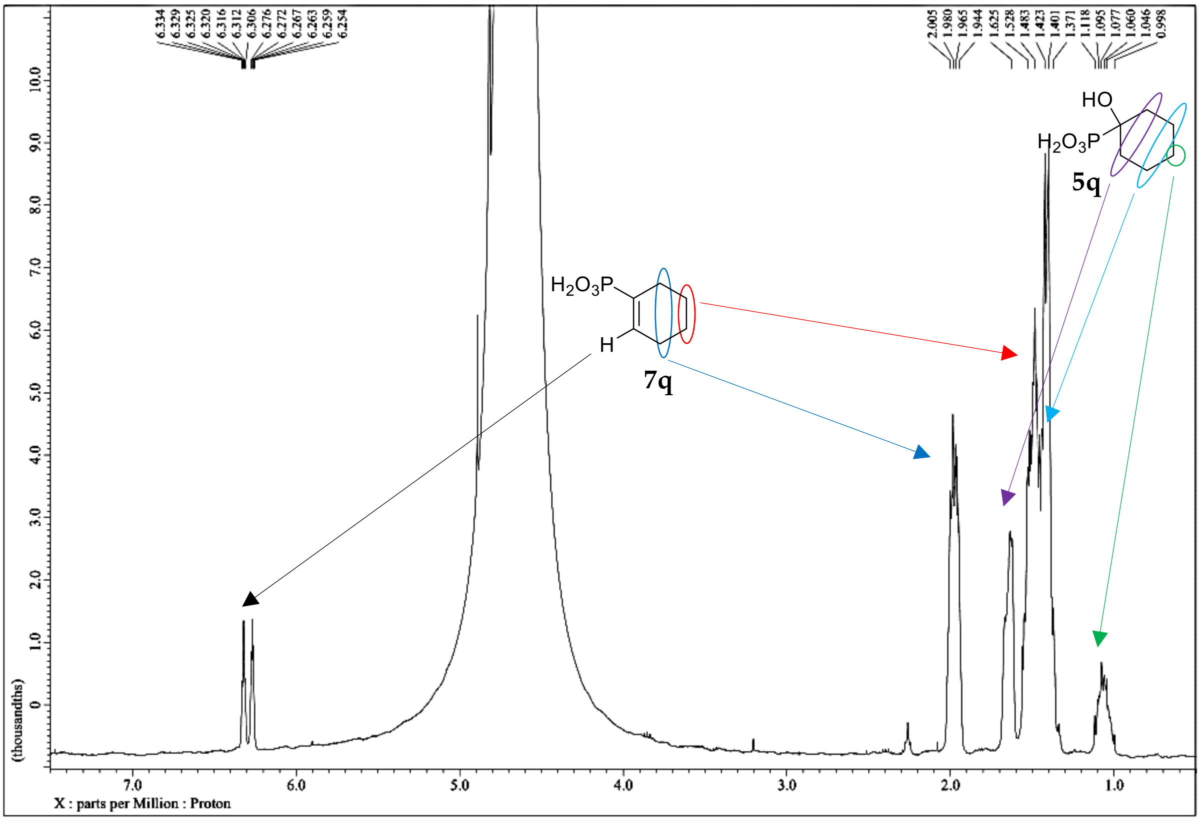

Figure S42. ^{1}H NMR spectra of the crude post-reaction mixture of 1-aminocyclohexylphosphonic acid (1q) with $NaNO_2$ after 98h with addition of reference material - 1-hydroxycyclohexylphosphonic acid (5q).

3.12. ABr1224. Deamination of **1f** in Water

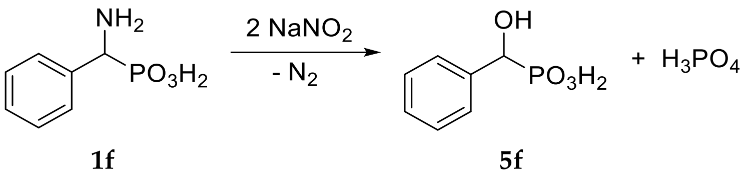

24 h	α = 1.00	97%	3%
δ_P [ppm]		17.27 (d)	0.83

After 24 h at 21 °C, post-reaction mixture contained hydroxy(phenyl)methylphosphonic acid **(5f)** (97%molP) and orthophosphoric acid (3%molP) (Figure S43 and Figure S45).

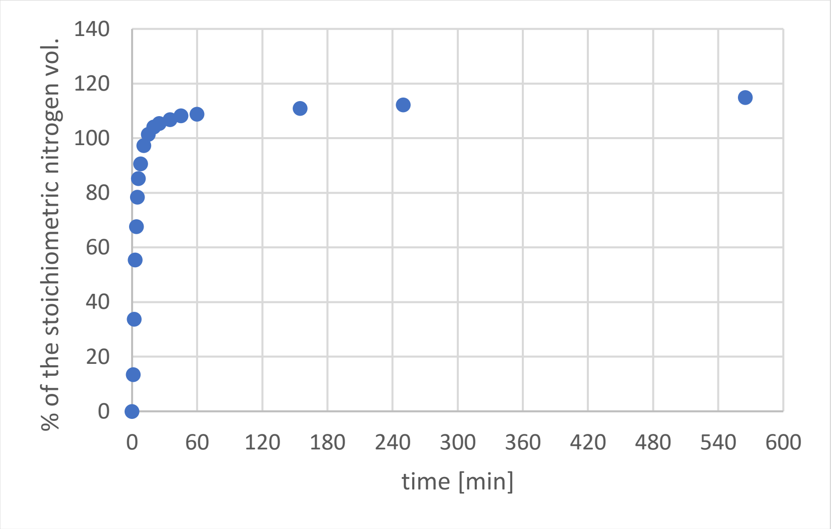

Chart 10. Dependence of the volume of the released gas (in % of the stoichiometric nitrogen volume) on the time in the reaction of amino(phenyl)methylphosphonic acid **(1f)** with $NaNO_2$ in water.

The presence of phenyl(hydroxy)methylphosphonic acid **(5f)** was confirmed by re-measuring the NMR spectra of the reaction mixture with the addition of reference material – phenyl(hydroxy)methylphosphonic acid **(5f)** (Figure S44 and Figure S46).

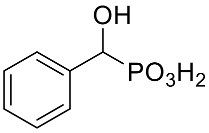

Hydroxy(phenyl)methylphosphonic acid (5f). ^{31}P NMR (D_2O): δ 17.27 (d, $^{2}J_{H\text{-}P}$ = 12.2 Hz); ^{1}H NMR (D_2O) δ: 7.32-7.38 (m, 2H), 7.26-7.32 (m, 2H), 7.20-7.26 (m, 1H), 4.72 (d, 1H, $^{2}J_{H\text{-}P}$ = 12.2 Hz).

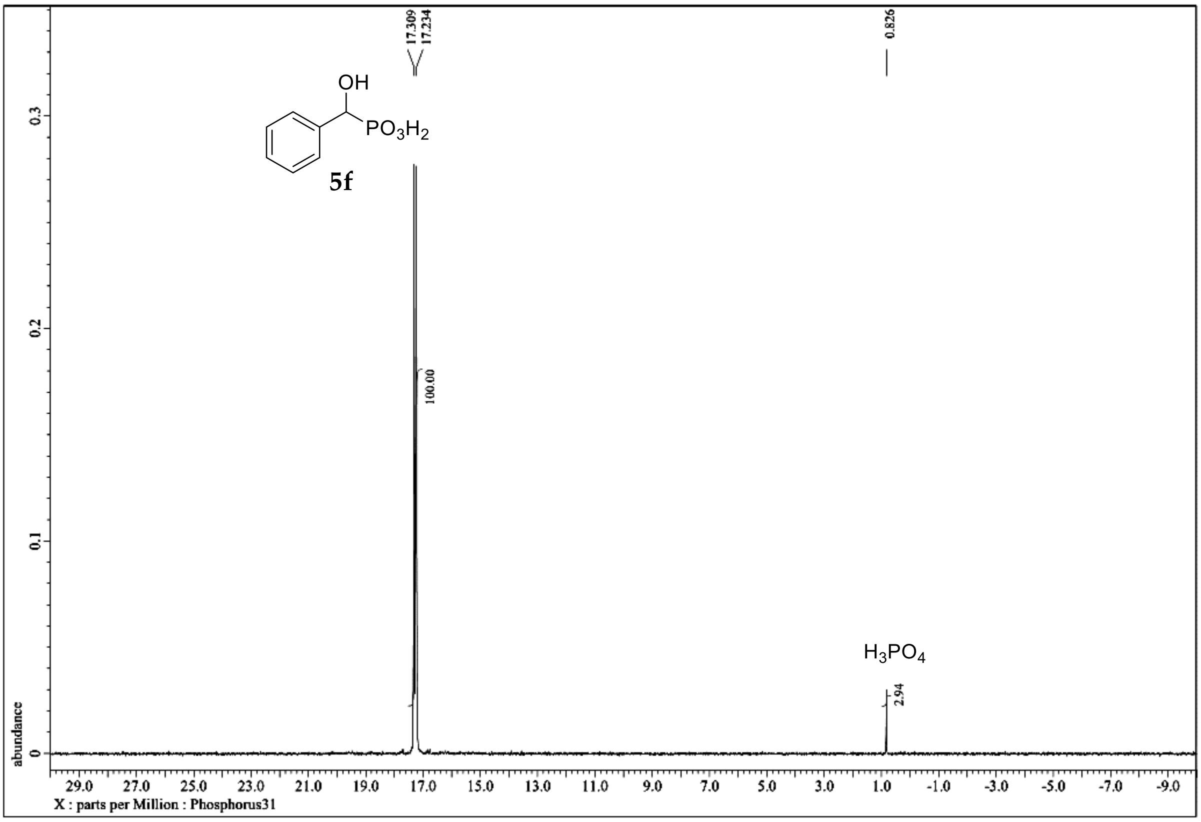

Figure S43. ^{31}P NMR (D_2O, 162MHz) spectra of the crude post-reaction mixture of amino(phenyl)methylphosphonic acid **(1f)** with $NaNO_2$ after 24 h.

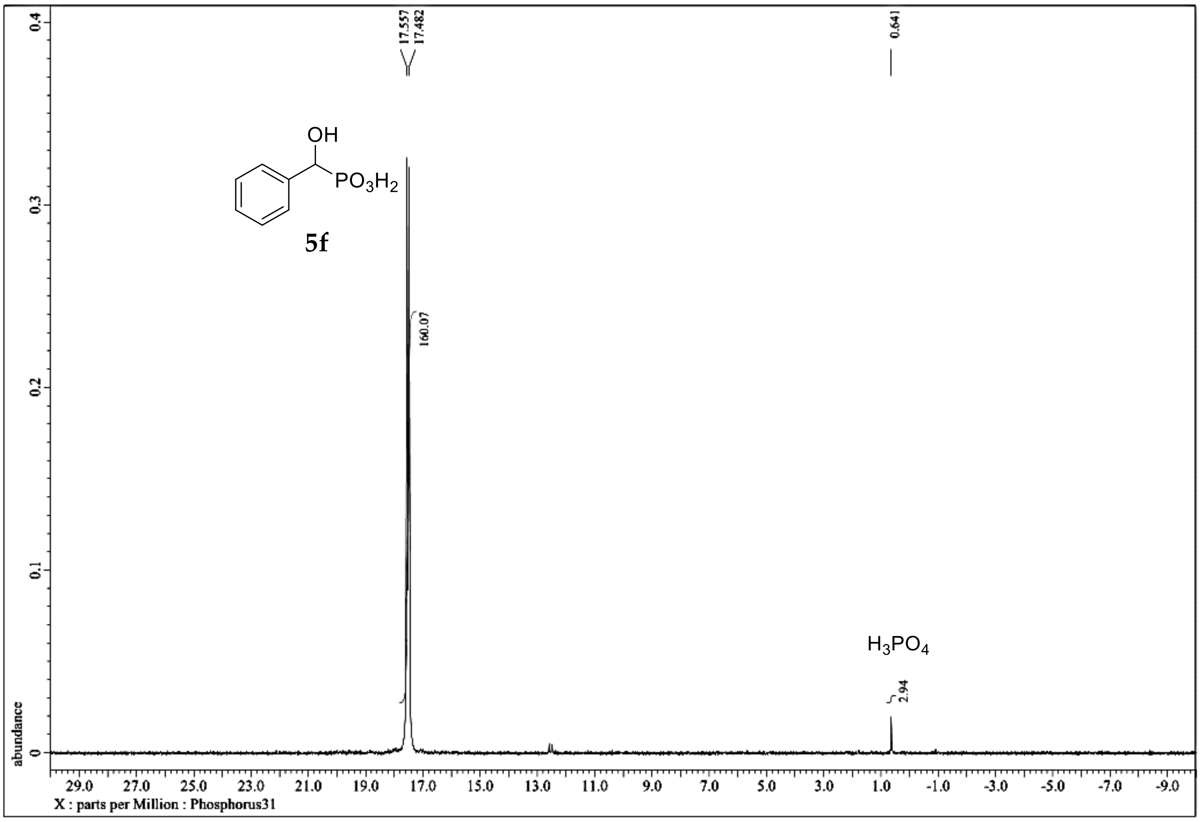

Figure S44. ^{31}P NMR (D_2O, 162MHz) spectra of the crude post-reaction mixture of amino(phenyl)methylphosphonic acid **(1f)** with $NaNO_2$ after 24 h with addition of reference material - hydroxy(phenyl)methylphosphonic acid **(5f)**.

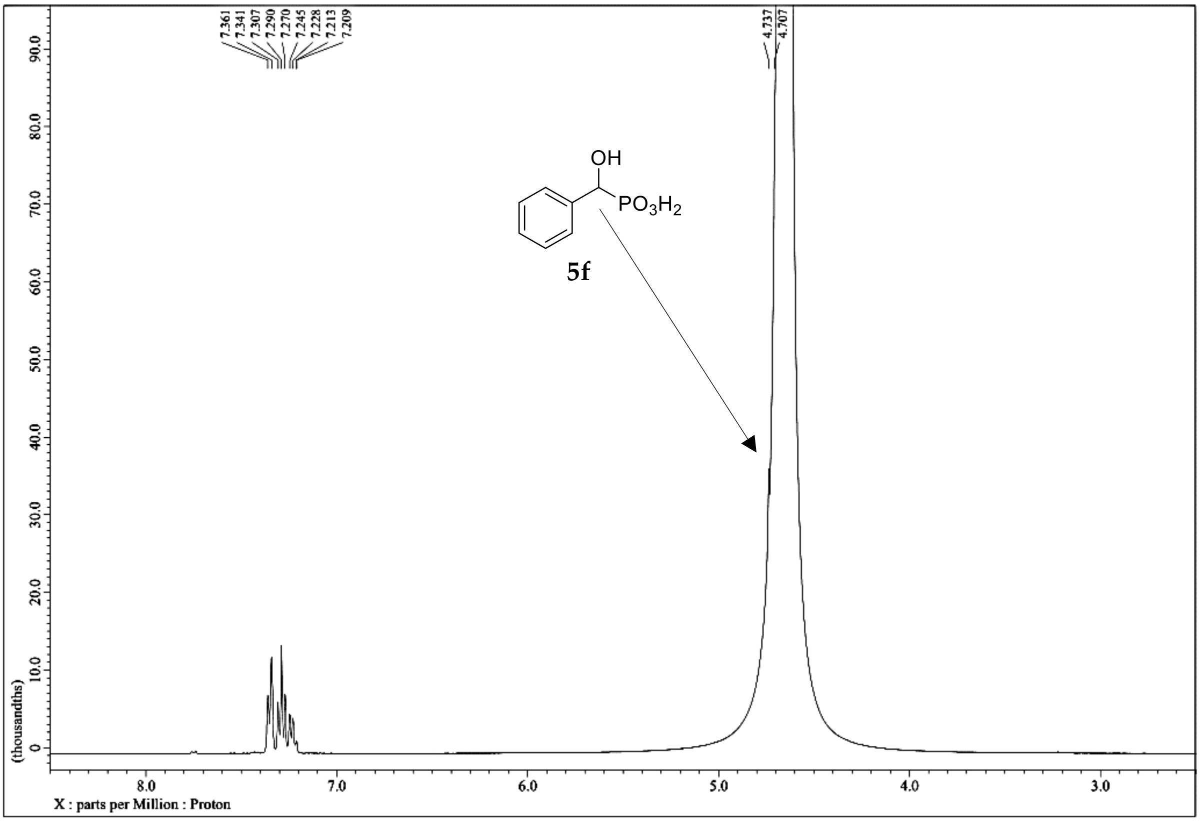

Figure S45. ^{1}H NMR (D_2O, 400MHz) spectra of the crude post-reaction mixture of amino(phenyl)methylphosphonic acid **(1f)** with $NaNO_2$ after 24 h.

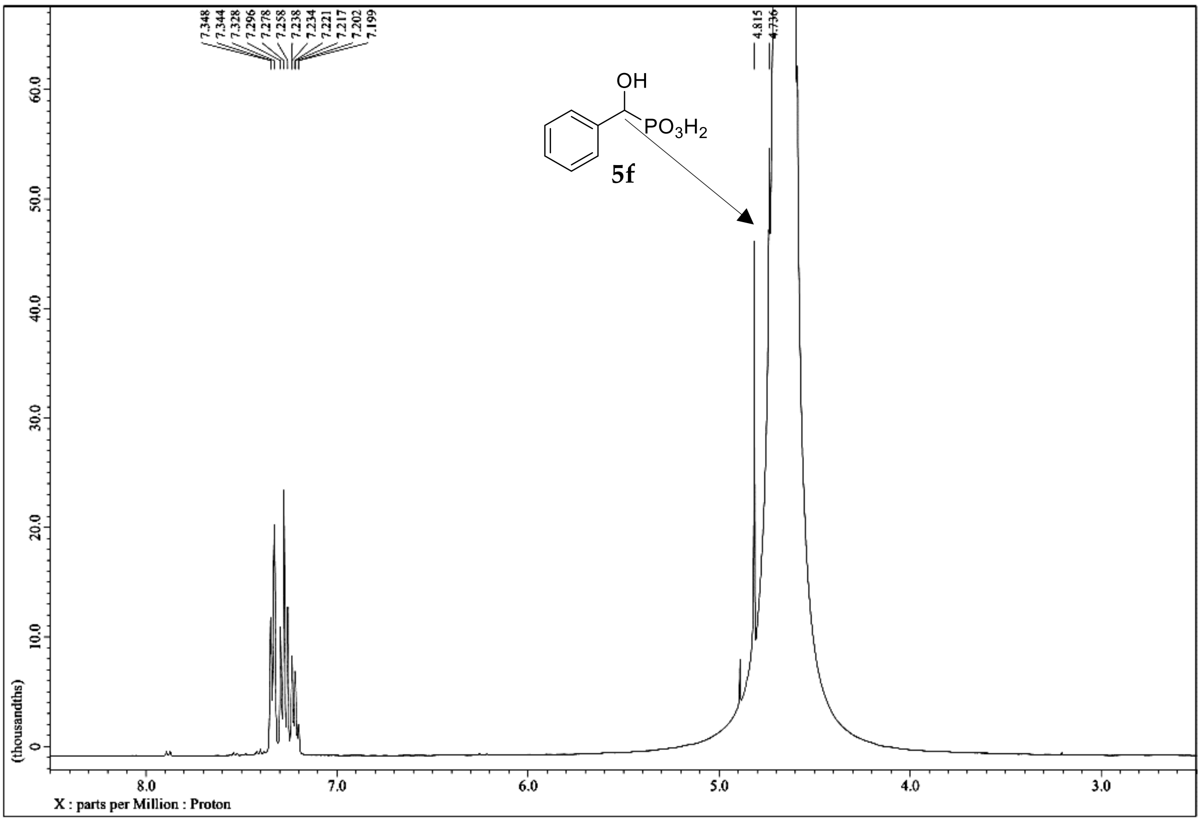

Figure S46. ^{1}H NMR (D_2O, 400MHz) spectra of the crude post-reaction mixture of amino(phenyl)methylphosphonic acid **(1f)** with $NaNO_2$ after 24 h with addition of reference material - hydroxy(phenyl)methylphosphonic acid **(5f)**.

3.13. ABr1226. Deamination of ***1n*** *in Water*

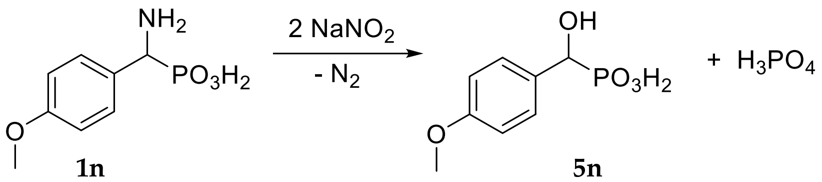

96h	α = 1.00	72%	25%
δ_P [ppm]		17.23 (d)	1.68

After 144h at 21 °C a suspension was formed. Liquid phase contained hydroxy(4-metoxyphenyl)methylphosphonic acid **(5n)** (72%molP), orthophosphoric acid (25%molP) and 3 unitentified compounds (3%molP in total) (Figure S47 and Figure S48). Precipitate was filtred, washed with water (2 x 1 mL), dried under reduced pressure and orange solid (30mg) was obtained (Figure S51 and Figure S52).

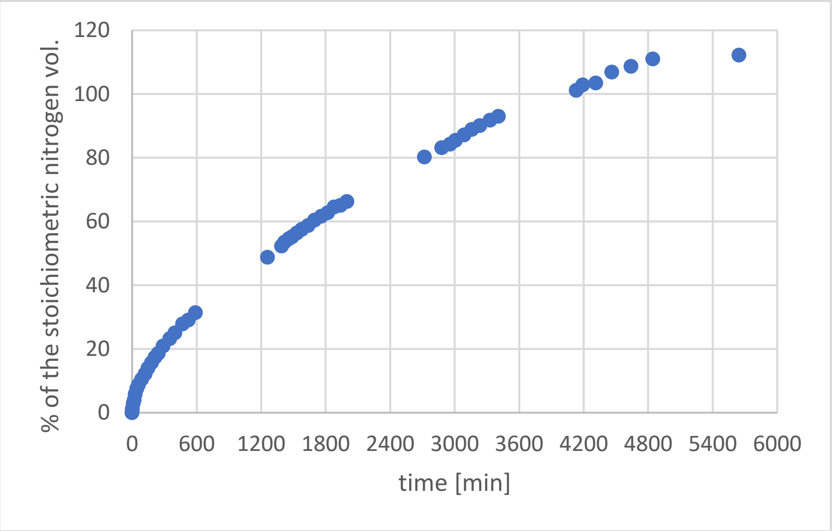

Chart 11. Dependence of the volume of the released gas (in % of the stoichiometric nitrogen volume) on the time in the reaction of amino(4-methoxyphenyl)methylphosphonic acid **(1n)** with $NaNO_2$ in water.

Hydroxy(4-metoxyphenyl)methylphosphonic acid **(5n)** was identified by comparing chemical shifts, multiplecity and coupling constants on ^{1}H and ^{31}P NMR spectra with description in the literature (Table S16). Spectra of crude hydroxy(4-metoxyphenyl)methylphosphonic acid **(5n)** was additionally re-measured with addition of D_2SO_4, in order to shift HOD signal overlapped on -C(PO_3H_2)H- (Figure S49 and Figure 50).

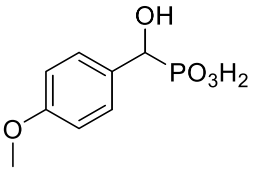

Hydroxy(4-metoxyphenyl)methylphosphonic acid (5n). ^{31}P NMR (D_2O): δ 17.23 (d, $^{2}J_{H\text{-}P}$ = 12.2 Hz); ^{1}H NMR (D_2O) δ: 7.27 (dd, 2H, $^{3}J_{H\text{-}H}$ = 8.9 Hz, J = 1.8 Hz), 6.83-6.86 (m, 2H), 3.69 (s, 3H).

Table S16. Comparison of chemical shifts and coupling constants on ^{1}H and ^{31}P NMR spectra of the reaction products of amino(4-metoxyphenyl)methylphosphonic acid **(1n)** with $NaNO_2$ in water with description in the literature.

**Structure**	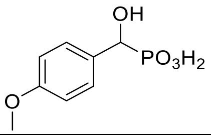		
	**ABr1226**	**ABr1226**	**Reference [17]**
**Solvent**	**D_2O**	**$D_2O + D_2SO_4$**	**D_2O**
**^{31}P NMR**	17.23 (d, J = 12.2 Hz)	20.90 (d, J = 11.2 Hz)	20.8
**^{1}H NMR**	7.27 (dd, 2H, *J* = 8.9, 1.8 Hz)	6.78 (dd, 2H, *J* = 8.8, 2.1 Hz)	7.12 (d, 2H, *J* = 8.0 Hz)
	6.83-6.86 (m, 2H)	6.38 (d, 2H, *J* = 8.6 Hz)	6.67 (d, 2H, *J* = 8.0 Hz)
	Overlapped with HOD	4.38 (d, 1H, *J* = 11.6 Hz)	4.71 (d, 1H, *J* = 12.0 Hz)
	3.69 (s, 3H)	3.19 (s, 3H)	3.44 (s, 3H)

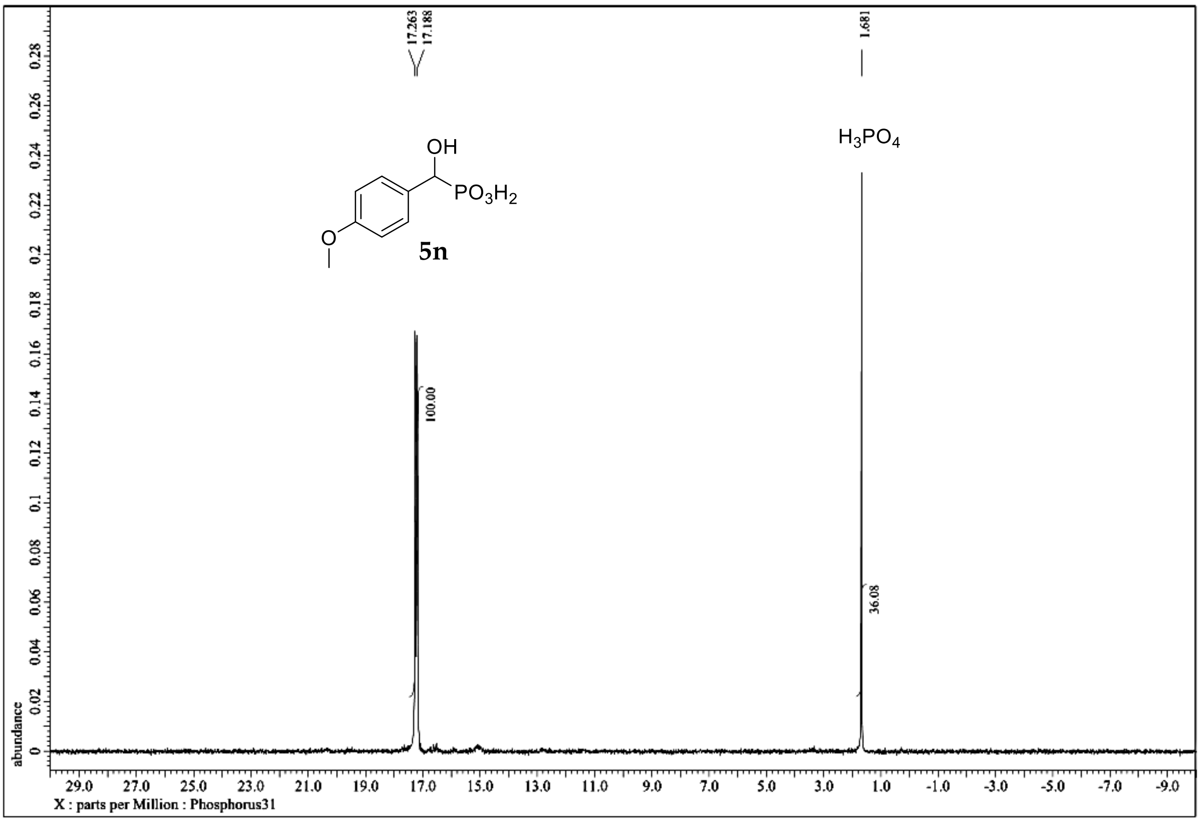

Figure S47. ^{31}P NMR (D_2O, 162MHz) spectra of the crude post-reaction mixture of amino(4-metoxyphenyl)methylphosphonic acid **(1n)** with $NaNO_2$ after 96h.

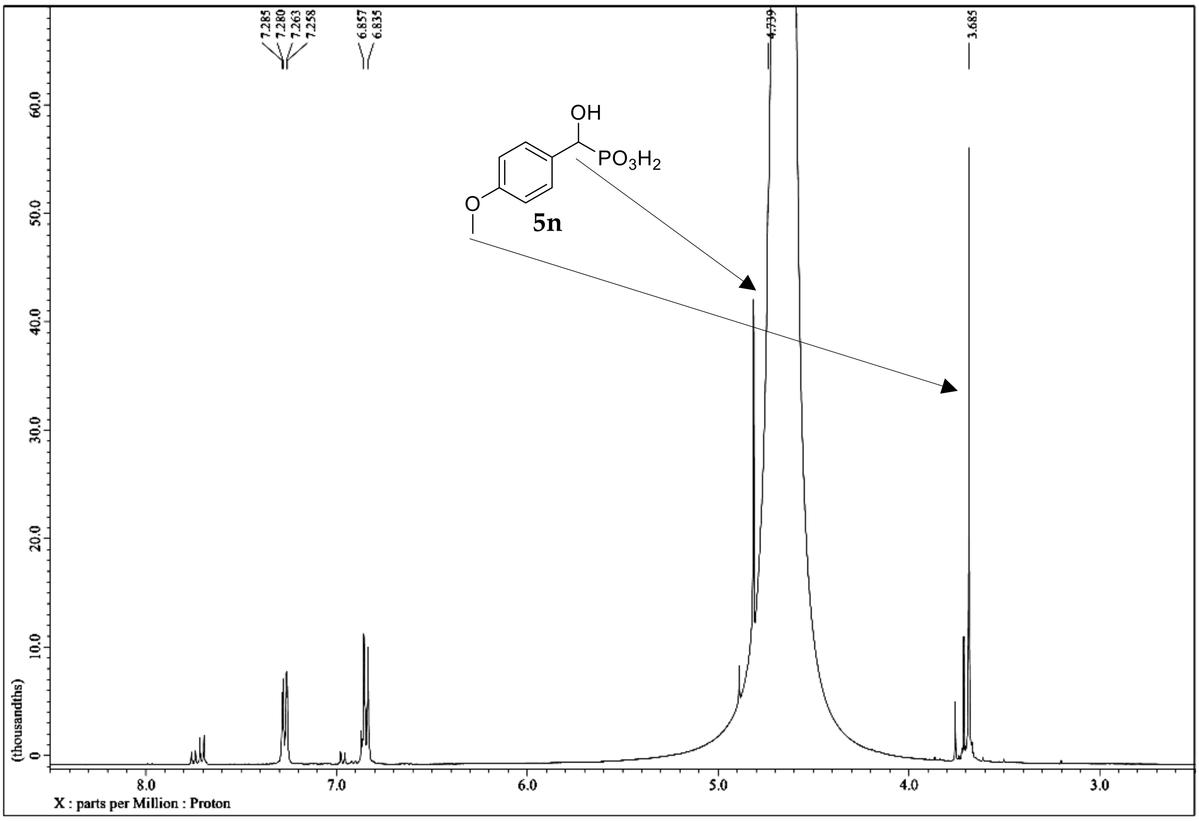

Figure S48. ^{31}P NMR (D_2O, 162MHz) spectra of the crude post-reaction mixture of amino(4-metoxyphenyl)methylphosphonic acid **(1n)** with $NaNO_2$ after 96h.

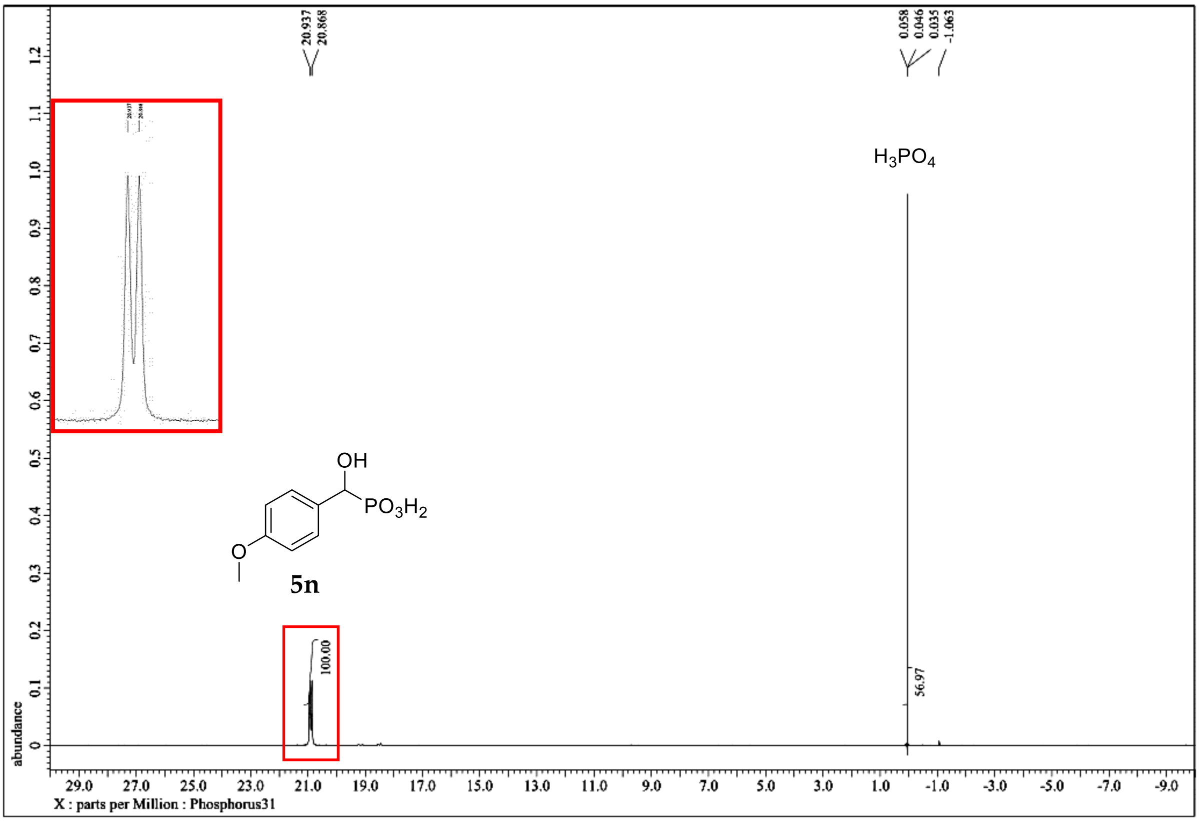

Figure S49. ^{31}P NMR (D_2O, 162MHz) spectra of the crude post-reaction mixture of amino(4-metoxyphenyl)methylphosphonic acid **(1n)** with $NaNO_2$ after 96h with addition of D_2SO_4.

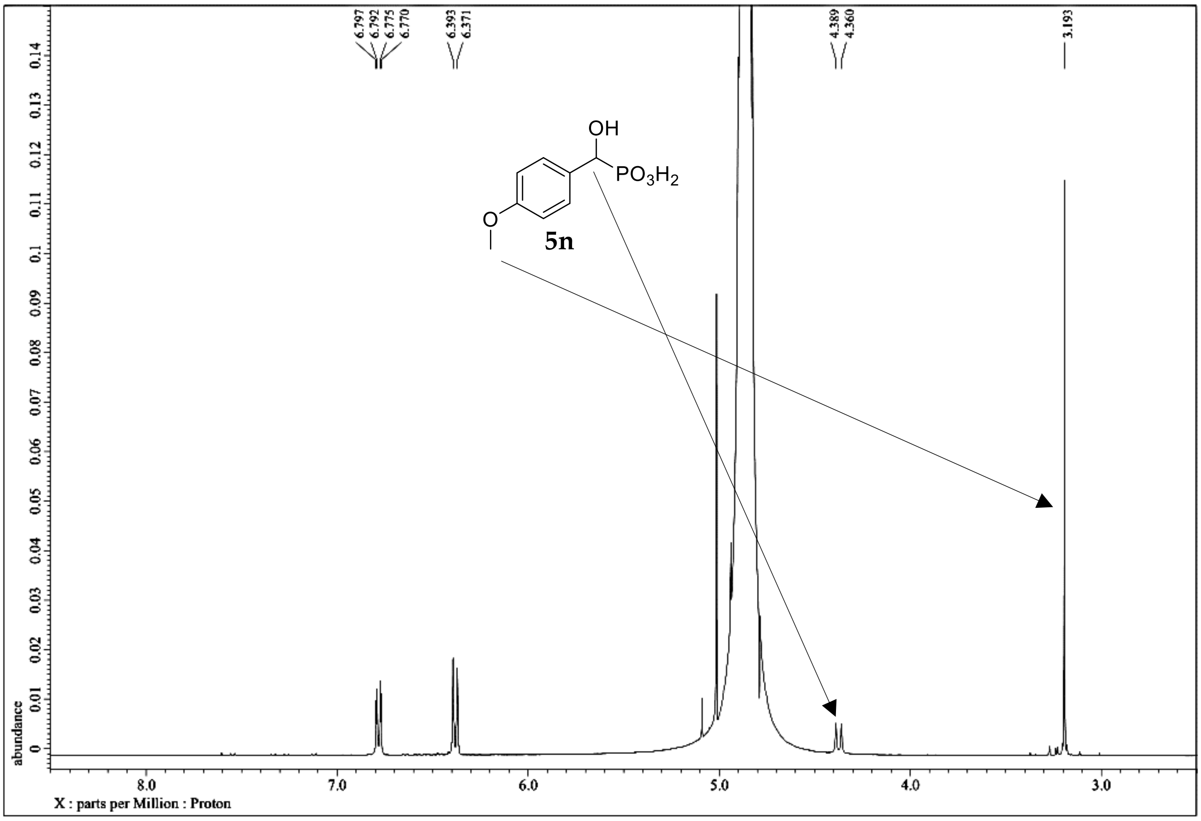

Figure 50. ^{1}H NMR (D_2O, 400MHz) spectra of the crude post-reaction mixture of amino(4-metoxyphenyl)methylphosphonic acid **(1n)** with $NaNO_2$ after 96h with addition of D_2SO_4.

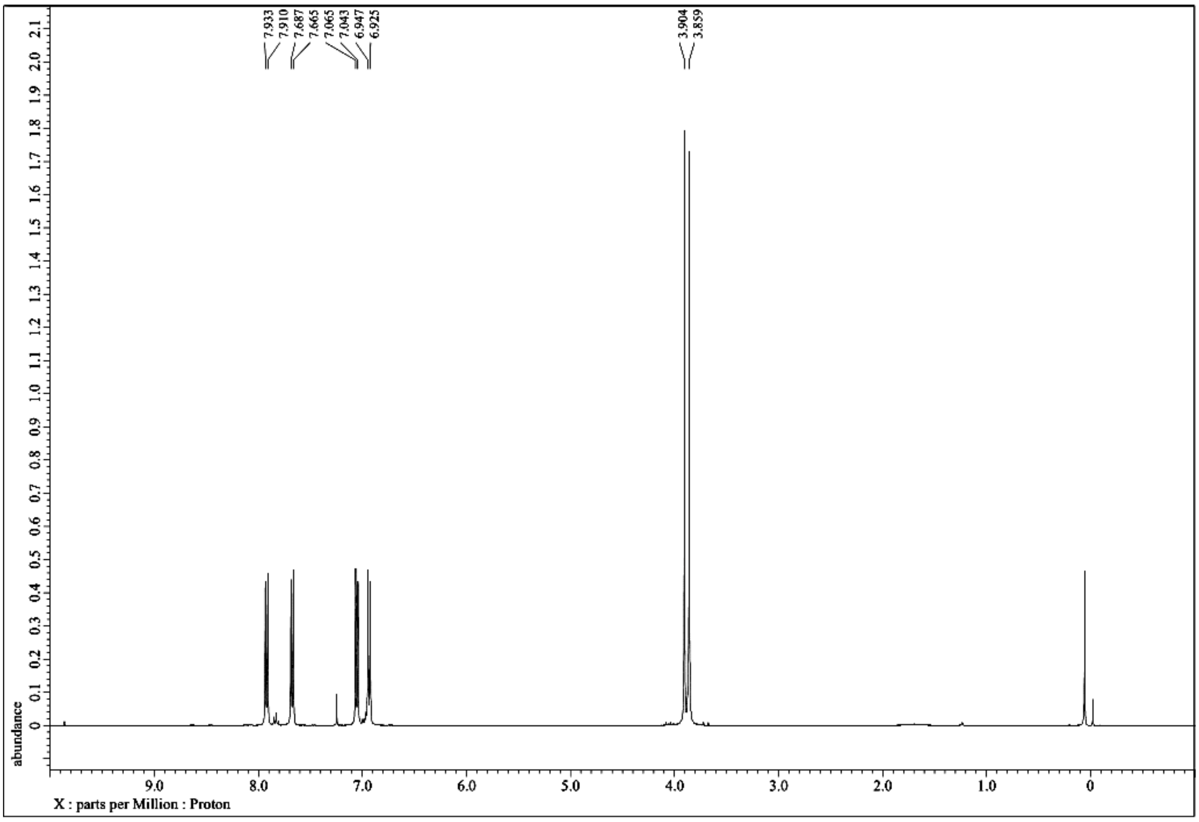

Figure S51. ^{1}H NMR (D_2O, 400MHz) spectra of the side product of the reaction of amino(4-metoxyphenyl)methylphosphonic acid **(1n)** with $NaNO_2$.

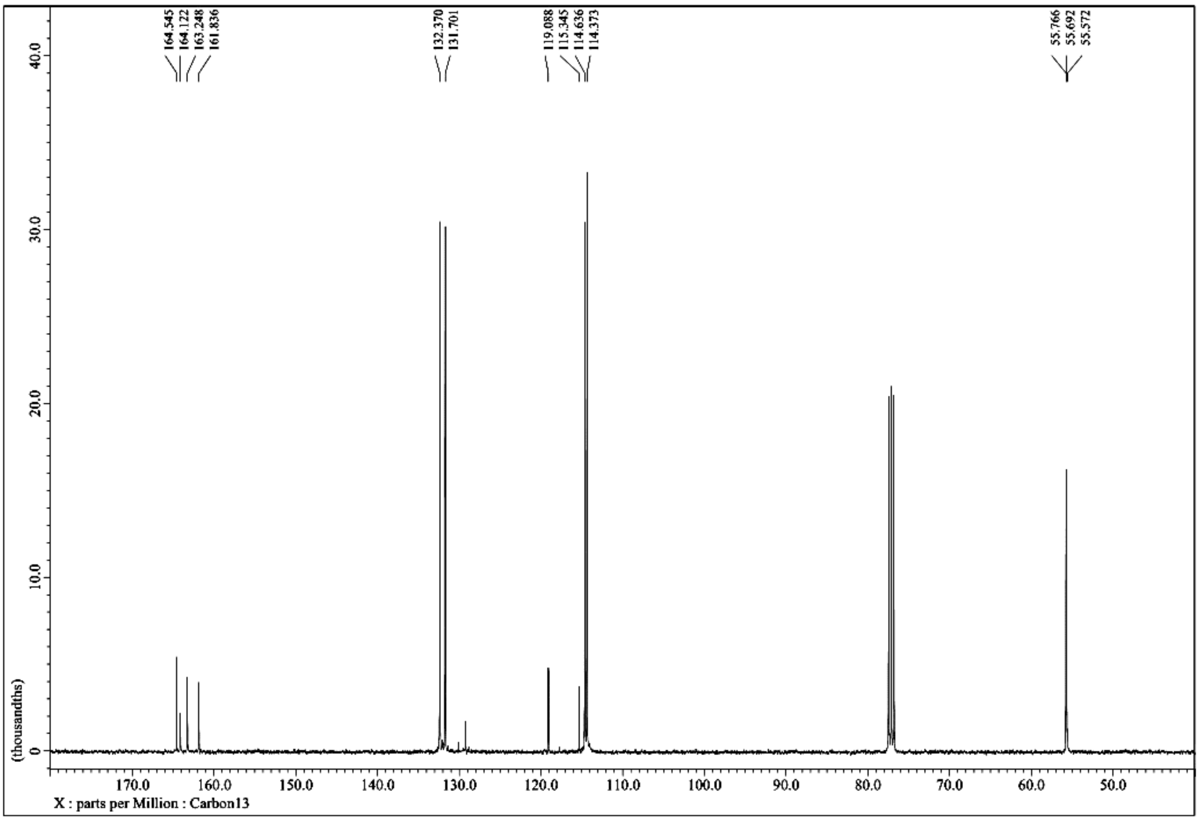

Figure S52. ^{13}C{^{1}H} NMR (D_2O, 100MHz) spectra of the side product of the reaction of amino(4-metoxyphenyl)methylphosphonic acid **(1n)** with $NaNO_2$.

*3.14. ABr1228. Deamination of **1h** in Water*

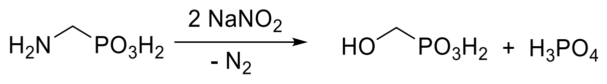

1h **5h**

24 h	α = 1.00	74%	9%
δ_P [ppm]		18.50 (t)	1.36

After 24 h at 21 °C, post-reaction mixture contained hydroxymethylphosphonic acid **(5h)** (74%molP), orthophosphoric acid (9%molP) and 8 unidentified compounds (17%molP in total) (Figure S60 and Figure S61).

Hydroxymethylphosphonic acid **(5h)** was identified by comparing chemical shifts, multiplecity and coupling constants on ^{1}H and ^{31}P NMR spectra with description in the literature (Table S18).

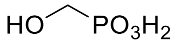

Hydroxymethylphosphonic acid (5h). ^{31}P NMR (D_2O): δ 18.50 (t, $^2J_{H-P}$ = 7.5 Hz); ^{1}H NMR (D_2O) δ: 3.53 (d, 2H, $^2J_{H-P}$ = 7.4 Hz).

Table S18. Comparison of chemical shifts and coupling constants on ^{1}H and ^{31}P NMR spectra of hydroxymethylphosphonic acid **(5h)** with description in the literature.

**Structure**	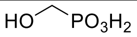	
	**ABr1228**	**Reference [18]**
**Solvent**	**D_2O**	**D_2O (pH = 6.8)**
**^{31}P NMR**	18.50 (t, J = 7.5 Hz)	17.5 (J = 7.9 Hz)

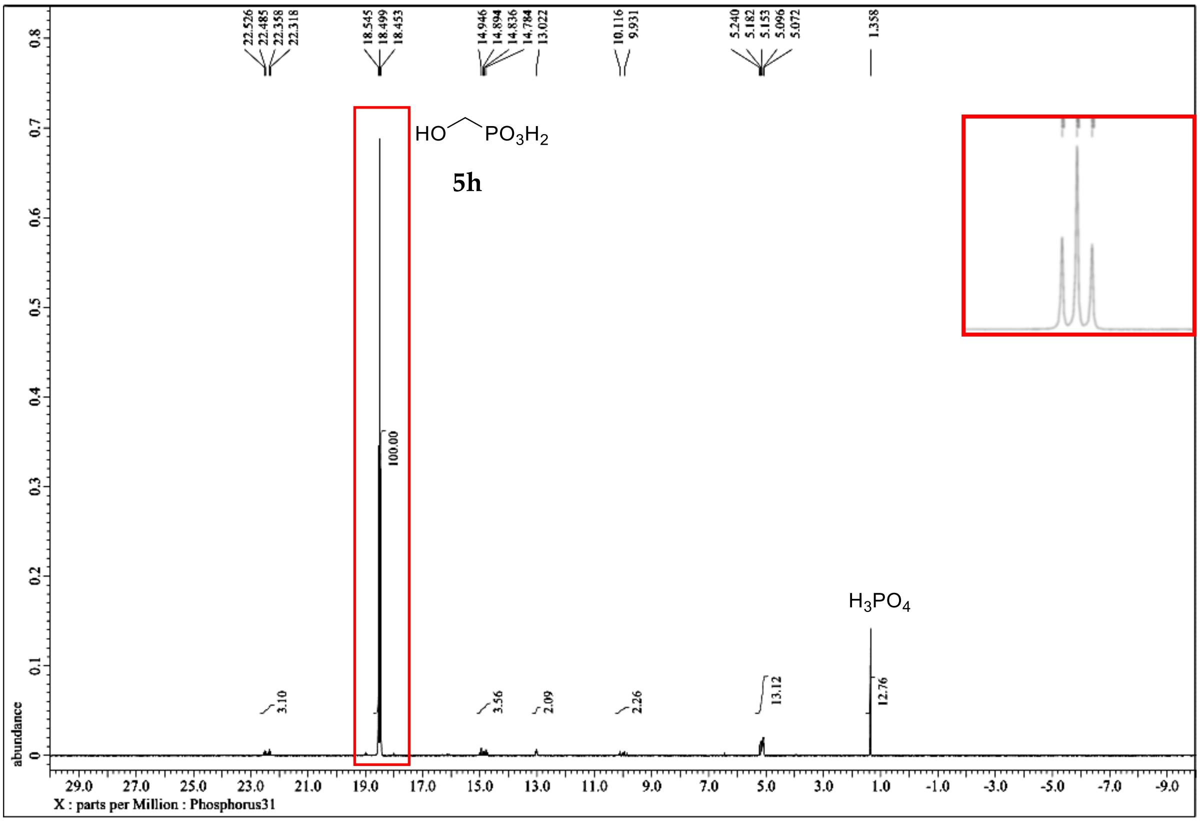

Figure S60. ^{31}P NMR (D_2O, 162MHz) spectra of the crude post-reaction mixture of 1aminomethylphosphonic acid **(1h)** with $NaNO_2$ after 24 h.

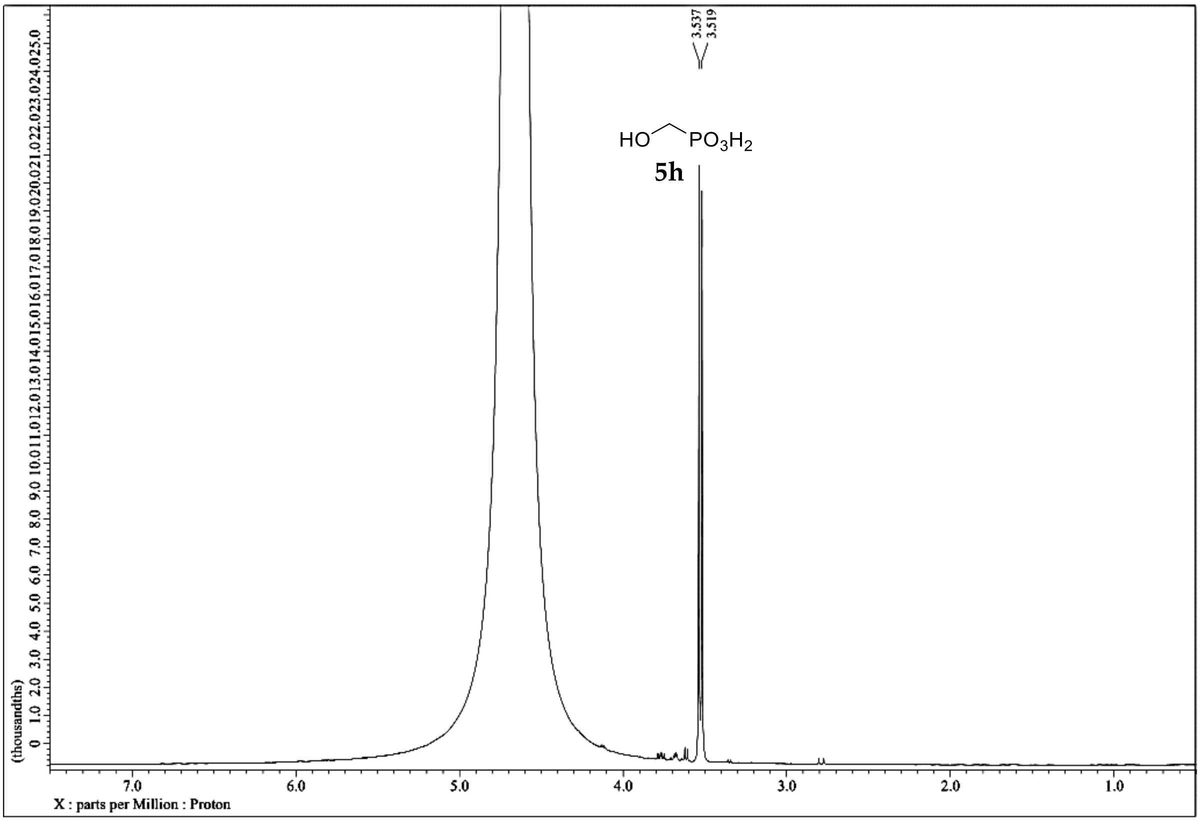

Figure S61. ^{1}H NMR (D_2O, 400MHz) spectra of the crude post-reaction mixture of 1aminomethylphosphonic acid **(1h)** with $NaNO_2$ after 24 h.

*3.15. ABr1234. Deamination of **1p** in Water*

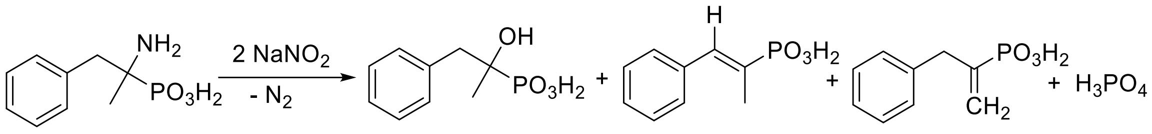

1p **5p** **7p** **7′p**

96h	α = 1.00	66%	22%	9%	3%
δ_P [ppm]		23.47 (dqq)	16.91 (dq)	14.53 (ddt)	1.10

After 96h at 21 °C, post-reaction mixture contained 1-hydroxy-1-methyl-2-phenylethylphosphonic acid **(5p)** (66%molP), (*E*)-3-phenyl-prop-2-en-2-ylphosphonic acid (22%molP) **(7p)**, 3-phenyl-prop-1-en-2-ylphosphonic acid **(7′p)** (9%molP) and orthophosphoric acid (3%molP) (Figure S62 and Figure S63).

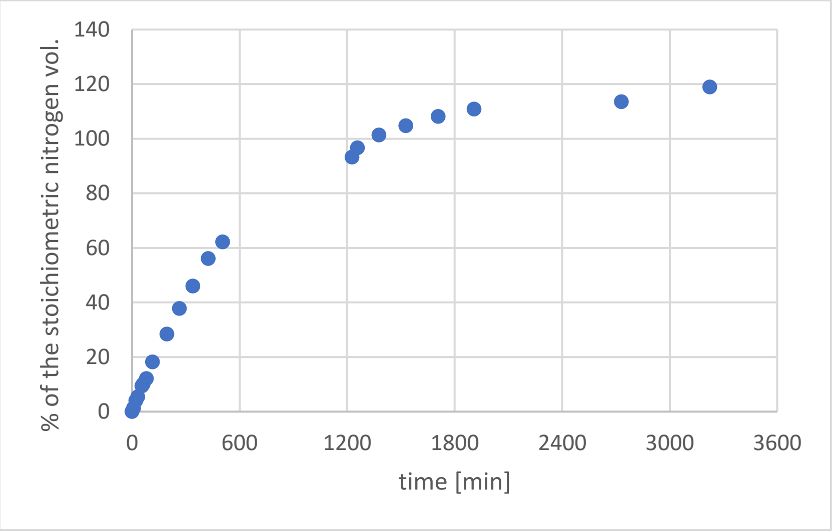

Chart 13. Dependence of the volume of the released gas (in % of the stoichiometric nitrogen volume) on the time in the reaction of 1-amino-1-methyl-2-phenylethylphosphonic acid **(1p)** with $NaNO_2$ in water.

(*E*)-3-Phenyl-prop-2-en-2-ylphosphonic acid **(7p)** was identified by comparing chemical shifts, multiplecity and coupling constants on ^{1}H and ^{31}P NMR spectra with description of structurally similar (*E*)-1-methylprop-1-en-1-ylphosphonic acid **(7i)**, which was obtained in the reaction of 1-amino-1-methylpropylphosphonic acid **(1i)** with $NaNO_2$ (ABr1214) (Table S19). 1-Hydroxy-1-methyl-2-phenylethylphosphonic acid **(5p)** and 3-phenyl-prop-1-en-2-ylphosphonic acid **(7′p)** were identified by comparing chemical shifts, multiplecity and coupling constants on ^{1}H and ^{31}P NMR spectra with description of structurally similar compunds: 1-hydroxy-1-methylethylphosphonic acid **(5m)** and 1-methylvinylphosphonic acid **(7m)**, which were obtained in the reaction of 1-amino-1-methylethylphosphonic acid **(1m)** with $NaNO_2$ (ABr1204) (Table S20).

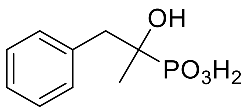

1-Hydroxy-1-methyl-2-phenylethylphosphonic acid (5p). ^{31}P NMR (D_2O): δ 23.47 (ddq, $^3J_{H\text{-}P}$ = 14.0 Hz, $^3J_{H\text{-}P}$ = 7.7 Hz, $^3J_{H\text{-}P}$ = 3.7 Hz); ^{1}H NMR (D_2O) δ: 7.13-7.34 (m, 5H), 2.97 (dd, 1H, $^2J_{H\text{-}H}$ = 13.8 Hz, $^3J_{H\text{-}P}$ = 7.6 Hz), 2.79 (dd, 1H, $^2J_{H\text{-}H}$ = 13.8 Hz, $^3J_{H\text{-}P}$ = 4.0 Hz), 1.03 (d, 3H, $^3J_{H\text{-}P}$ = 14.4 Hz).

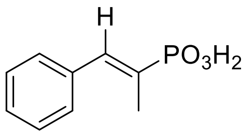

(*E*)-3-Phenyl-prop-2-en-2-ylphosphonic acid (7p). ^{31}P NMR (D_2O): δ 16.91 (dq, $^3J_{H-P(cis)}$ = 22.9 Hz, $^3J_{H-P}$ = 14.0 Hz); ^{1}H NMR (D_2O) δ: 7.13-7.34 (m, 5H), 7.03 (dq, 1H, $^3J_{H-P}$ = 22.9 Hz, $^4J_{H-H}$ = 1.5 Hz), 1.90 (dd, 3H, $^3J_{H-P}$ = 14.2 Hz, $^4J_{H-H}$ = 1.5 Hz).

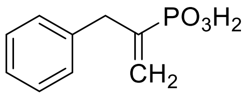

3-Phenyl-prop-1-en-2-ylphosphonic acid (7′p). ^{31}P NMR (D_2O): δ 14.53 (ddt, $^3J_{H-P(trans)}$ = 43.9 Hz, $^3J_{H-P(cis)}$ = 20.6 Hz, $^3J_{H-P}$ = 9.4 Hz); ^{1}H NMR (D_2O) δ: 7.13-7.34 (m, 5H), 5.64 (ddt, 1H, $^3J_{H-P\,(cis)}$ = 20.5 Hz, $^4J_{H-H}$ = 1.5 Hz, $^4J_{H-H}$ = 1.2 Hz), 5.07 (ddt, 1H, $^3J_{H-P(trans)}$ = 43.3 Hz, $^4J_{H-H}$ = 1.8 Hz, $^4J_{H-H}$ = 1.5 Hz), 3.43 (ddd, 2H, $^3J_{H-P}$ = 9.8 Hz, *J* undetermined).

Table S19. Comparison of chemical shifts and coupling constants on ^{1}H and ^{31}P NMR spectra of *(E)*-3-phenyl-prop-2-en-2-ylphosphonic acid **(7p)** and *(E)*-1-methylprop-1-en-1-ylphosphonic acid **(7i)** (ABr1214).

**Structure**	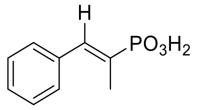	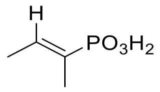
**Source**	**ABr1234**	**ABr1214**
**Solvent**	**D2O**	**D2O**
**^{31}P NMR**	16.91 (dq, *J* = 22.9, 14.0 Hz)	17.47 (dqq, *J* = 21.7, 13.6, 3.7 Hz)
**^{1}H NMR**	7.13-7.34 (m, 5H)	
	7.03 (dq, 1H, *J* = 22.9, 1.5 Hz)	6.15 (ddq, 1H, *J* = 21.7, 6.7, 1.5 Hz)
	1.90 (dd, 3H, *J* = 14.2, 1.5 Hz)	1.63 (ddq, 3H, *J* = 13.6, 1.2, 1.2 Hz)
		1.57 (ddq, 3H, *J* = 6.7, 3.7, 1.2 Hz)

Table S20. Comparison of chemical shifts and coupling constants on ^{1}H and ^{31}P NMR spectra of post-reaction mixture of 1-amino-2-phenyl-1-methylethylphosphonic acid **(1p)** with $NaNO_2$ and 1-hydroxy-1-methylethylphosphonic acid **(5m)** and 1-methylvinylphosphonic acid **(7m)**.

**Structure**	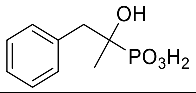	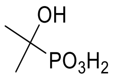	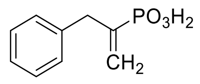	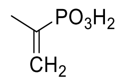
**Source**	**ABr1234**	**ABr1204**	**ABr1234**	**ABr1204**
**Solvent**	**D2O**	**D2O**	**D2O**	**D2O**
**^{31}P NMR**	23.47 (ddq, *J* = 14.0, 7.7, 3.7 Hz)	23.84 (septet, *J* = 14.0 Hz)	14.53 (ddt, *J* = 43.9, 20.6, 9.4 Hz)	15.21 (ddq, *J* = 43.9, 20.6, 13.1 Hz)
**^{1}H NMR**	7.13-7.34 (m, 5H)		7.13-7.34 (m, 5H)	
	2.97 (dd, 1H, *J* = 13.8, 7.6 Hz)		5.64 (ddt, 1H, *J* = 20.5, 1.5, 1.2 Hz),	5.46 (ddq, 1H, *J* = 20.3, 2.1, 1.2 Hz)
	2.79 (dd, 1H, *J* = 13.8, 4.0 Hz)		5.07 (ddt, 1H, *J* = 43.3, 1.8, 1.5 Hz)	5.36 (tq, 1H, *J* = 43.4, 1.8 Hz)
	1.03 (d, 3H, *J* = 14.4 Hz)	1.23 (d, 6H, *J* = 13.8 Hz)	3.43 (ddd, *J* = 9.8 Hz, *J* undetermined)	
				1.77 (dt, 3H, *J* = 13.1, 1.2 Hz)

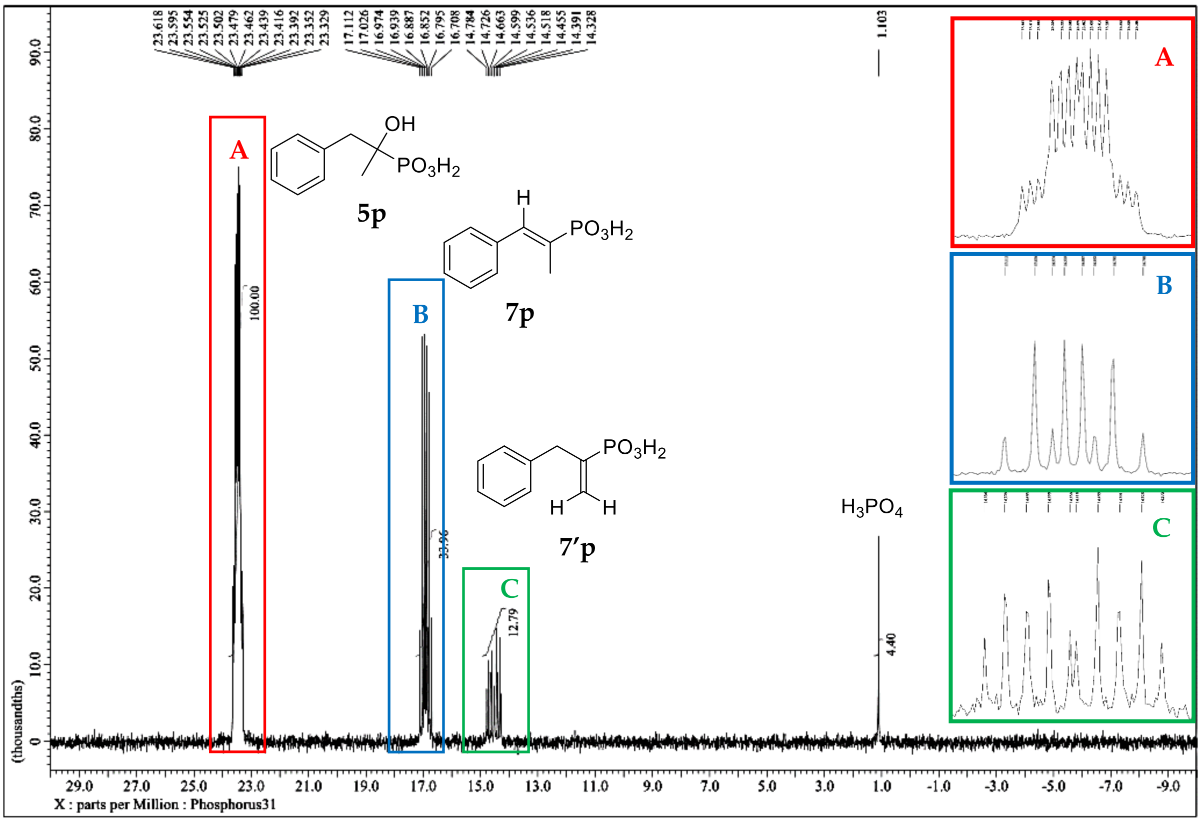

Figure S62. ^{31}P NMR (D_2O, 162MHz) spectra of the crude post-reaction mixture of 1-amino-1-methyl-2-phenylethylphosphonic acid **(1p)** with $NaNO_2$ after 96h.

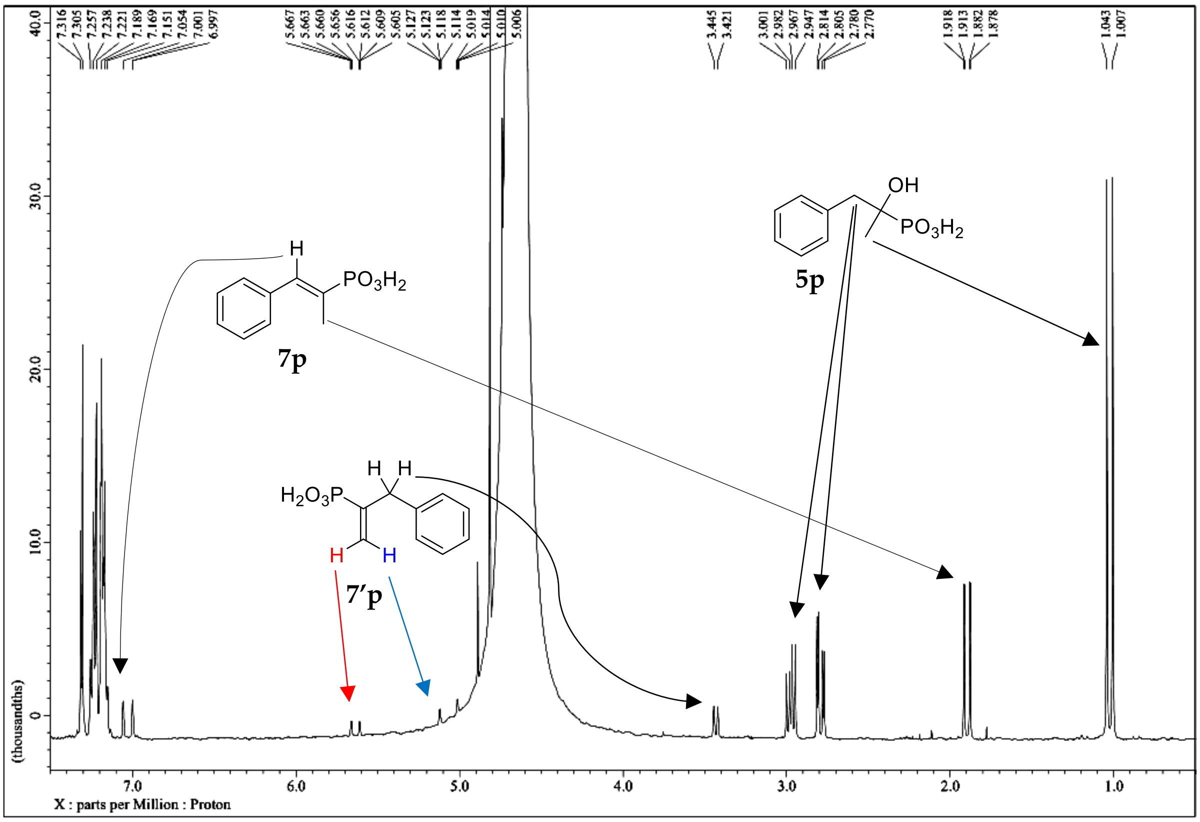

Figure S63. ^{1}H NMR (D_2O, 400MHz) spectra of the crude post-reaction mixture of 1-amino-1-methyl-2-phenylethylphosphonic acid **(1p)** with $NaNO_2$ after 96h.

3.16. *ABr1236. Deamination of* **1g** *in Water*

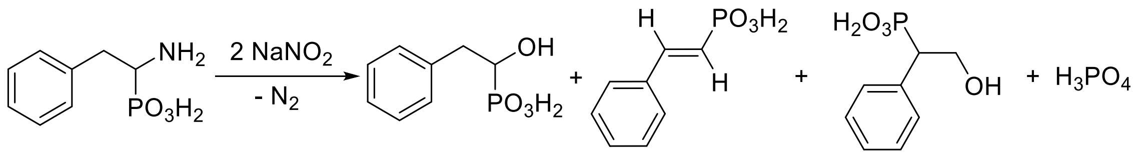

1g		5g	7g	5″g	
48h	α = 0.99	43%	3%	21%	25%
360h	α = 1.00	42%	3%	21%	29%
δ_P [ppm]	14.5	19.59 (ddd)	13.37 (t)	18.96 (ddd)	1.58

After 48h at 21 °C, post-reaction mixture contained 1-hydroxy-2-phenylethylphosphonic acid **(5g)** (44%molP), (*E*)-2-phenylvinylphosphonic acid **(7g)** (3%molP), 2-hydroxy-1-phenylethylphosphonic acid **(5″g)** (21%molP), orthophosphoric acid (26%molP), unreacted substrate **1g** (1%molP) and unidentified compounds (5%molP in total).

After 360h at 21 °C, post-reaction mixture contained 1-hydroxy-2-phenylethylphosphonic acid **(5g)** (46%molP), (*E*)-2-phenylvinylphosphonic acid **(7g)** (3%molP), 2-hydroxy-1-phenylethylphosphonic acid (23%molP) **(5″g)**, orthophosphoric acid (23%molP) and unidentified compounds (5%molP in total) (Figure S66 and Figure S66).

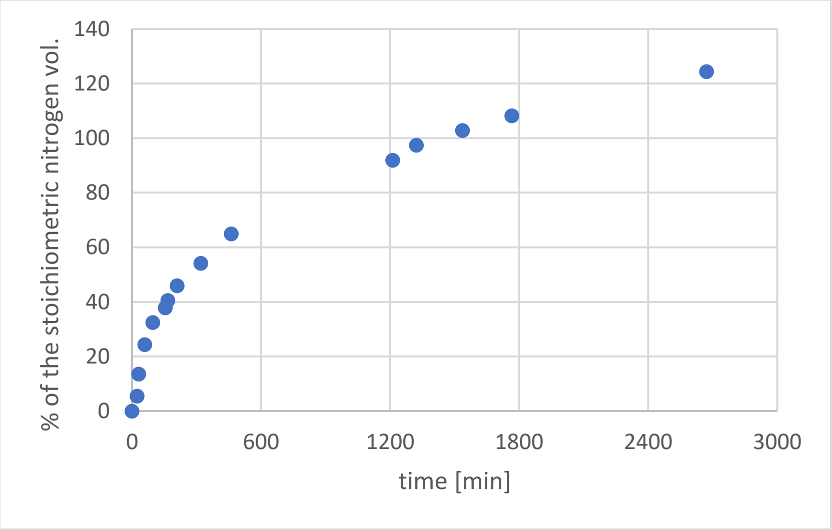

Chart 14. Dependence of the volume of the released gas (in % of the stoichiometric nitrogen volume) on the time in the reaction of 1-amino-2-phenylethylphosphononic acid **(1g)** with $NaNO_2$ in water.

1-Hydroxy-2-phenylethylphosphonic acid **(5g)** and (*E*)-2-phenylvinylphosphonic acid **(7g)** were identified by comparing chemical shifts, multiplecity and coupling constants on ^1H and ^{31}P NMR spectra with description in the literature (Table S21).

In the reaction 3 hydroxyalkylphosphonic acid could be formed (Figure S64): 1-hydroxy-2-phenylethylphosphonic acid **(7g)** (product of nucleophile addition), 2-hydroxy-2-phenylethylphosphonic acid **(5′g)** (product of rearragement and nucleophile addition) and 2-hydroxy-1-phenylethylphosphonic acid **(5″g)** (product of phenyl migration and nucleophile addition).

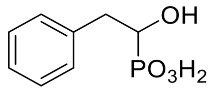

5g

1-hydroxy-2-phenylethylphosphonic acid

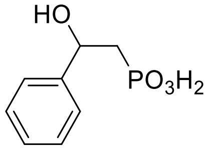

5′g

2-hydroxy-2-phenylethylphosphonic acid

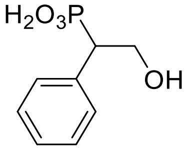

5″g

2-hydroxy-1-phenylethylphosphonic acid

Figure S64. Structures of plausible products of reaction of 1-amino-2-phenylethylphosphonic acid **(1g)** with $NaNO_2$ in water.

Identification of reaction products was accomplished by analysis of $^{13}C\{^1H\}$ NMR spectra (Figure S67) and DEPT-135 spectra (Figure S68): in the post-reaction mixture there were 2 compounds that have structure $-CH_2CH(PO_3H_2)-$. The comparison of $^{13}C\{^1H\}$ NMR spectra of post-reaction mixture with description in the literature confirms that the main reaction product was 1-hydroxy-2-phenylethylphosphonic acid **(5g)** [δ_C: 70.77 (d, *J* = 152.8 Hz), 38.01]. Therefore, 2-hydroxy-1-phenylethylphosphonic acid **(5″g)** has to be the second reaction product [δ_C: 62.25, 50.11 (d, *J* = 124.1 Hz)]. Additionally, the NMR spectra description [19] of 2-hydroxy-2-phenylethylphosphonic acid **(5′g)** excludes possibility that this compound was present in the post-reaction mixture.

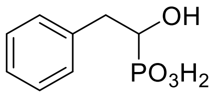

1-Hydroxy-2-phenylethylphosphonic acid (5g). ^{31}P NMR (D_2O): δ 19.59 (br d, $J_{H\text{-}P}$ = 2.8 Hz); ^1H NMR (D_2O, 600MHz) δ: 7.14-7.32 (m, 5H), 3.79 (ddd, 1H, $^2J_{H\text{-}P}$ = 11.5 Hz, $^3J_{H\text{-}H}$ = 7.8 Hz, $^3J_{H\text{-}H}$ = 2.5 Hz), 3.03 (ddd, 1H, $^2J_{H\text{-}H}$ = 14.3 Hz, $^3J_{H\text{-}P}$ = 2.5 Hz, $^3J_{H\text{-}H}$ = 2.5 Hz), 2.64 (ddd, 1H, $^2J_{H\text{-}H}$ = 14.3 Hz, $^3J_{H\text{-}P}$ = 11.9 Hz, $^3J_{H\text{-}H}$ = 7.3 Hz). $^{13}C\{^1H\}$ NMR (62mg, D_2O) δ: 70.77 (d, $J_{C\text{-}P}$ = 152.8 Hz), 38.01.

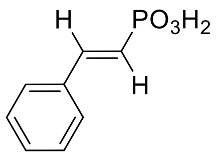

(*E*)-2-Phenylvinylphosphonic acid (7g). ^{31}P NMR (D_2O): δ 13.38 (m); ^1H NMR (D_2O) δ: 7.14-7.32 (m, 5H), 7.04 (dd, 1H, $J_{H\text{-}P}$ = 20.8 Hz, $^3J_{H\text{-}H}$ = 17.4 Hz), 6.37 (dd, 1H, $^3J_{H\text{-}H}$ = 17.4 Hz, $J_{H\text{-}P}$ = 16.0 Hz).

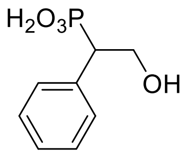

2-Hydroxy-1-phenylethylphosphonic acid (5″g). ^{31}P NMR (D_2O): δ 18.96 (br d $^2J_{H\text{-}P}$ = 21.5 Hz); ^1H NMR (D_2O) δ: 7.14-7.32 (m, 5H), 4.07 (dt, $^2J_{H\text{-}H}$ = 11.3 Hz, $^3J_{H\text{-}P}$ = 4.3 Hz, $^3J_{H\text{-}H}$ = 4.3 Hz), 3.96 (dt, 1H, $^2J_{H\text{-}H}$ = 11.3 Hz, $^3J_{H\text{-}P}$ = 5.2 Hz, $^3J_{H\text{-}H}$ = 5.2 Hz), 3.07 (ddd, 1H, $^2J_{H\text{-}P}$ = 22.0 Hz, $^3J_{H\text{-}H}$ = 11.0 Hz, $^3J_{H\text{-}H}$ = 4.3 Hz). $^{13}C\{^1H\}$ NMR (62mg, D_2O) δ: 62.25, 50.11 (d, $J_{C\text{-}P}$ = 124.1 Hz).

Table S21. Comparison of chemical shifts and coupling constants on ¹H and ³¹P NMR spectra of post-reaction mixture of 1-amino-2-phenylethylphosphonic acid **(1g)** with $NaNO_2$ with description in the literature.

**Structure**	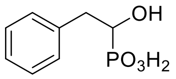		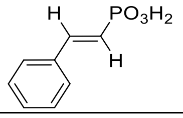	
**Source**	**ABr1236**	**Reference [20]**	**ABr1236**	**Reference [21]**
**Solvent**	**D_2O**	**D_2O**	**D_2O**	**DMSO-d_6**
**^{31}P NMR**	19.59 (ddd, *J* = 2.8 Hz)	18.22 (m, *J* = 6.9 Hz)	13.38 (dd, *J* = 19.6, 15.9 Hz);	14.71
**^{1}H NMR**	7.14-7.32 (m, 5H)	7.27-7.30 (4H, m)<br>7.22-7.27 (m, 1H)	7.14-7.32 (m, 5H)	7.6 (m, 2H)<br>7.4 (m, 3H),
	3.85 (ddd, 1H, *J* = 11.7, 7.7, 2.4 Hz)	3.77 (ddd, 1H, *J* = 7.5, 3.1, 9.6 Hz)	7.04 (dd, 1H, *J* = 20.8, 17.4 Hz)	7.2 (dd, 1H, *J* =17.7, 17.4 Hz)
	3.10 (dt, 1H, *J* = 13.9, 2.7 Hz)	3.07 (ddd, 1H, *J* = 14.4, 3.1, 3.4 Hz)	6.36 (dd, 1H, *J* = 17.4, 16.0 Hz)	6.5 (dd, 1H, *J* =18.0, 16.2 Hz)
	2.70 (ddd, 1H, *J* = 14.3, 11.9, 7.3 Hz)	2.66 (ddd, 1H, *J* = 14.4, 9.6, 7.8 Hz).		

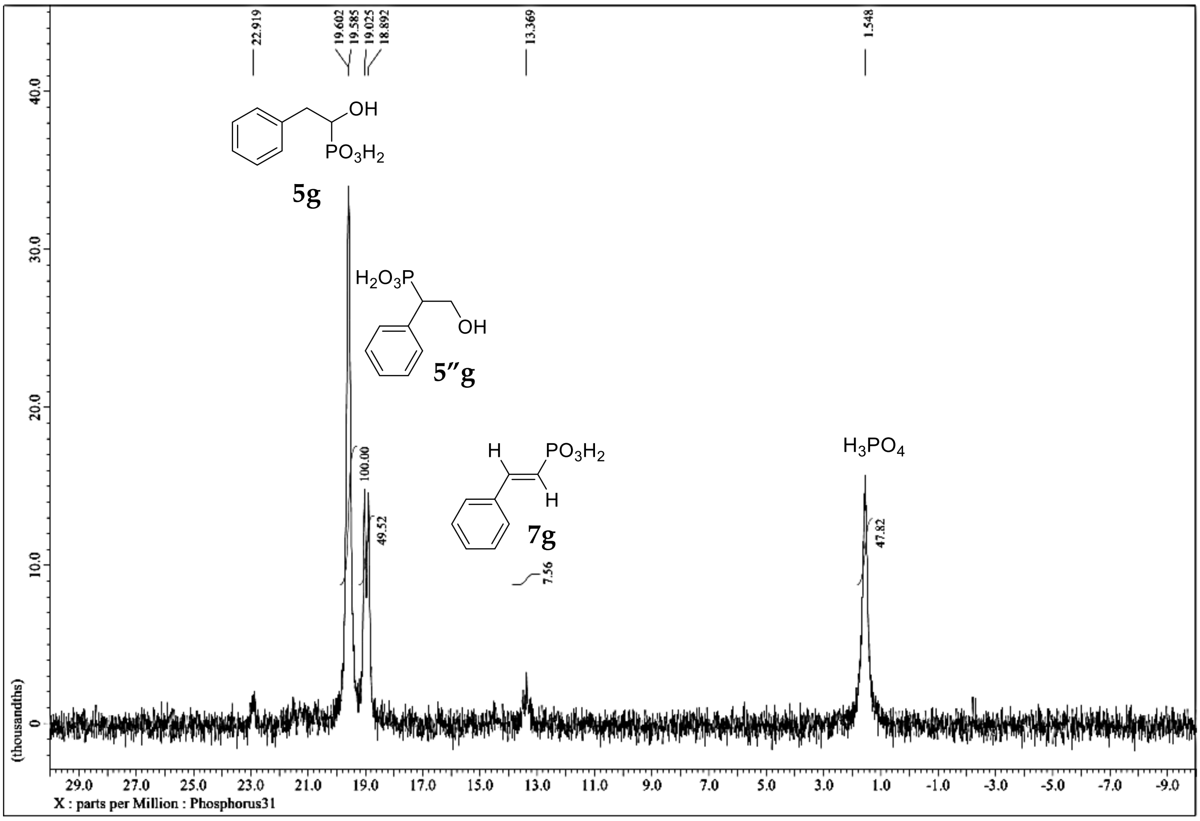

Figure S65. ^{31}P NMR (D_2O, 162MHz) spectra of the crude post-reaction mixture of 1-amino-2-phenylethylphosphonic acid **(1g)** with $NaNO_2$ after 360h.

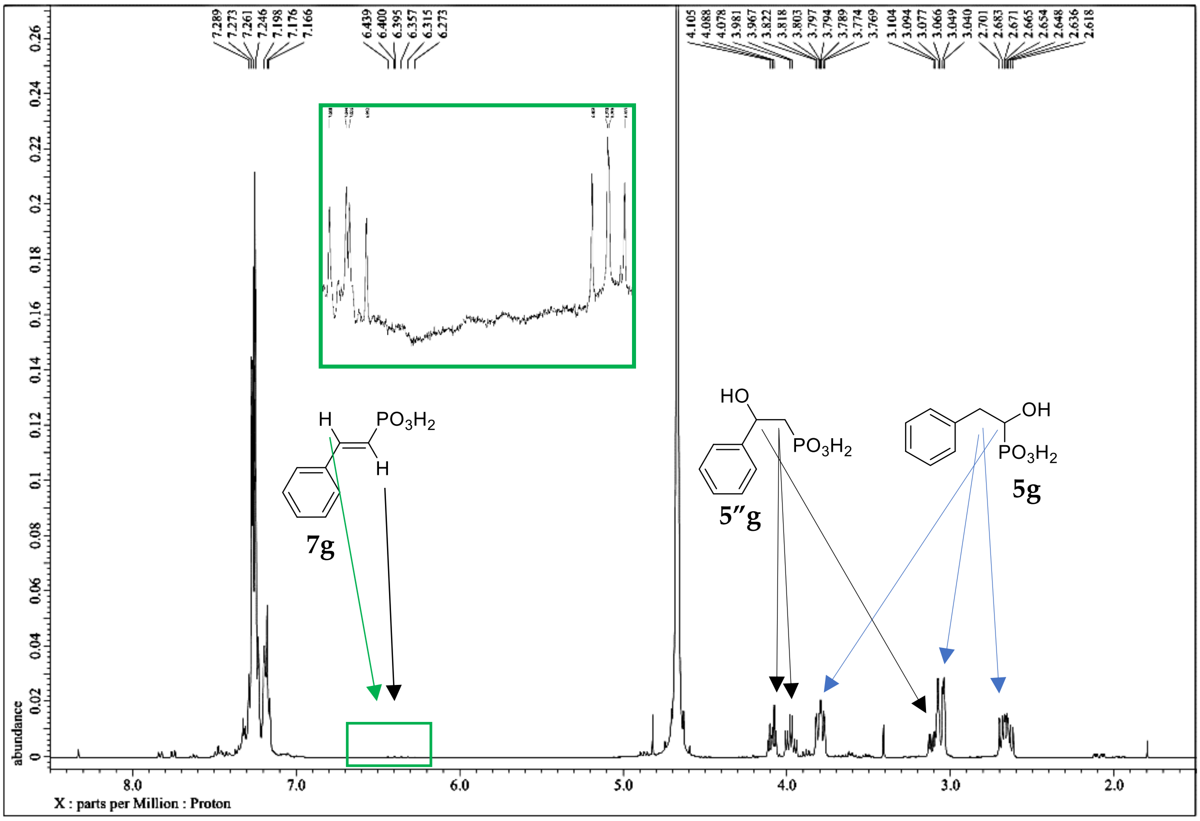

Figure S66. ^{1}H NMR (D_2O, 400MHz) spectra of the crude post-reaction mixture of 1-amino-2-phenylethylphosphonic acid **(1g)** with $NaNO_2$ after 360h.

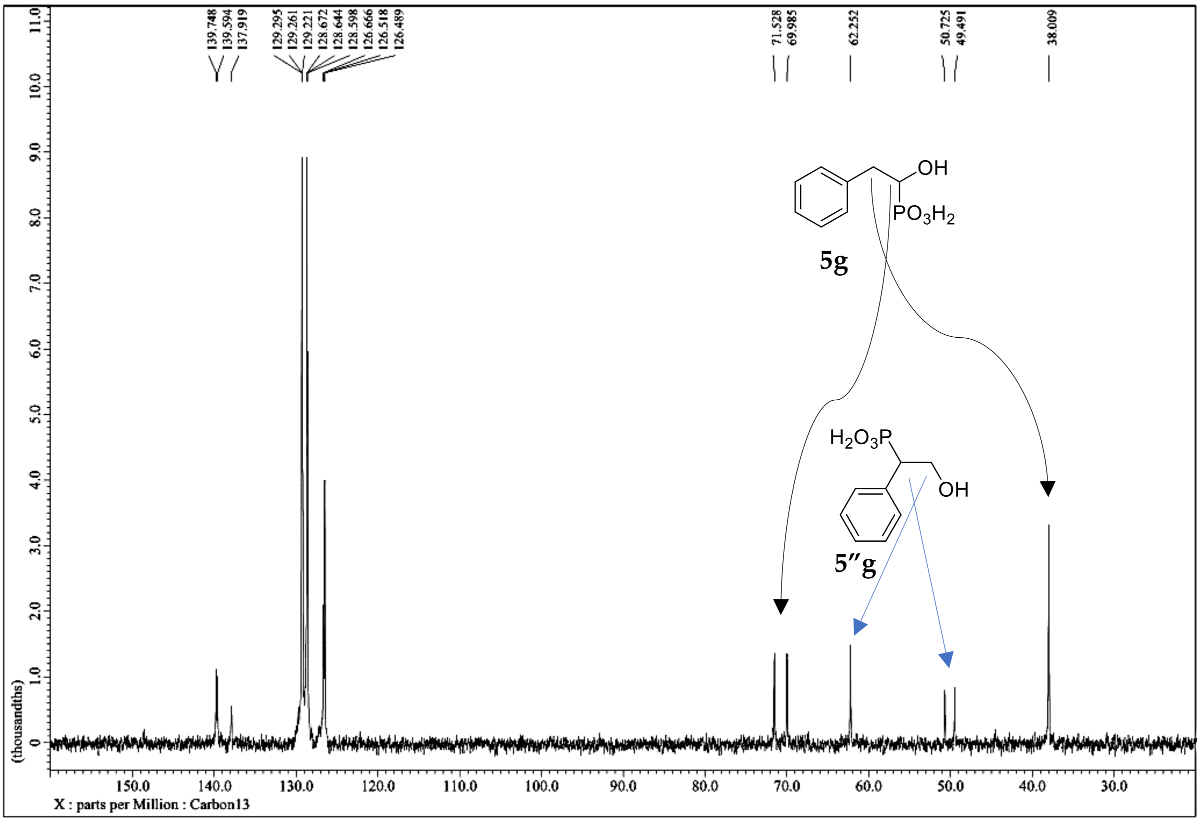

Figure S67. $^{13}C\{^{1}H\}$ NMR (D_2O, 100MHz) spectra of the concentrated post-reaction mixture of 1-amino-2-phenylethylphosphonic acid **(1g)** with $NaNO_2$ after 360h.

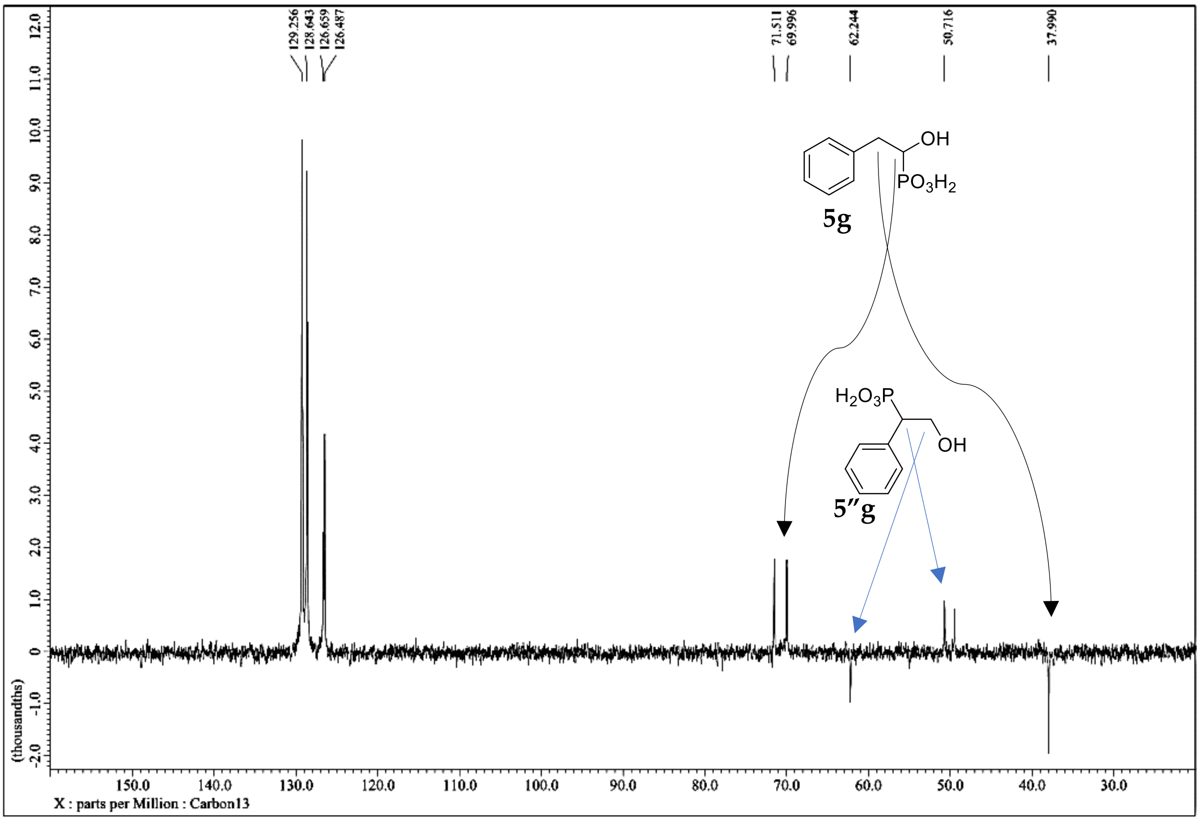

Figure S68. Dept-135 (D_2O) spectra of the concentrated post-reaction mixture of 1-amino-2-phenylethylphosphonic acid **(1g)** with $NaNO_2$ after 360h..

*3.17. ABr1248. Deamination of **1e** in Water*

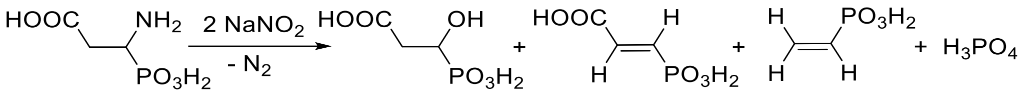

1e **5e** **7e** **7a**

48h	α = 0.71	55%	6%	6%	4%
264h	α = 0.86	70%	6%	6%	4%
δ_P [ppm]	12.23(dt)	19.07(dt)	13.11(dd)	10.65(ddd)	0.64(s)

After 48h at 21 °C, post-reaction mixture contained 3-hydroxy-3-phosphonopropanoic acid **(5e)** (55%molP), (*E*)-3-phosphonoacrylic acid **(7e)** (6%molP), vinylphosphonic acid **(7a)** (6%molP), orthophosphoric acid (4%molP) and unreacted substrate **1e** (29%molP).

After 264h post-reaction mixture contained 3-hydroxy-3-phosphonopropanoic acid **(5e)** (70%molP), (*E*)-3-phosphonoacrylic acid **(7e)** (6%molP), vinylphosphonic acid **(7a)** (6%molP), orthophosphoric acid (4%molP) and unreacted substrate **1e** (14%molP) (Figure S69 and Figure S70).

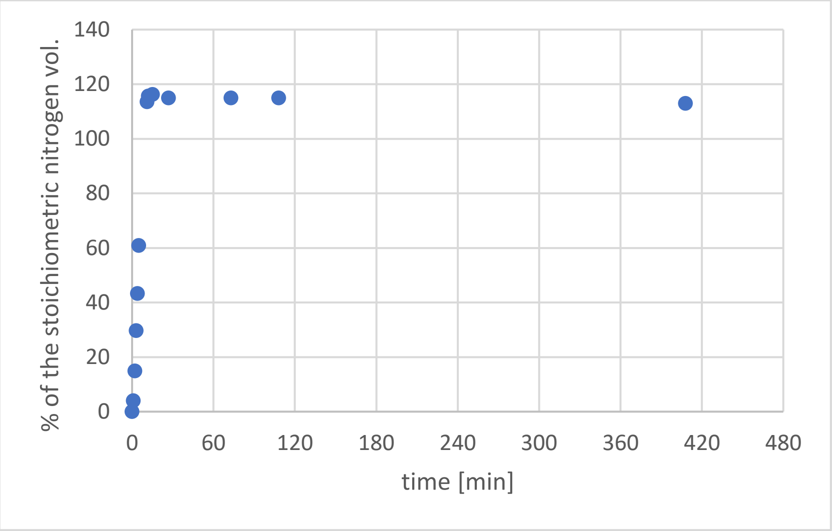

Chart 15. Dependence of the volume of the released gas (in % of the stoichiometric nitrogen volume) on the time in the reaction of 3-amino-3-phosphonopropanoic acid **(1e)** with $NaNO_2$ in water.

Spectra of 3-hydroxy-3-phosphonopropanoic acid **(5e)** has been not described in the literature therefore this compound was identified by comparing chemical shifts, multiplecity and coupling constants on ^1H and ^{31}P NMR spectra with description of structurally similar 1-hydroxy-2-phenylethylphosphonic acid **(5g)**, which was obtained in reaction of 1-amino-2-phenylethylphosphonic acid **(1g)** with $NaNO_2$ (ABr1236) (Table S22). (*E*)-3-phosphonoacrylic acid **(7e)** and vinylphosphonic acid **(7a)** were identified by comparing chemical shifts, multiplecity and coupling constants on ^1H and ^{31}P NMR spectra with description in the literature (Table S23).

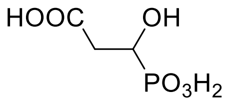

3-Hydroxy-3-phosphonopropanoic acid (5e). ^{31}P NMR (D_2O): δ 19.07 (dt, $^2J_{H\text{-}P}$ = 8.0 Hz, $^3J_{H\text{-}P}$ = 8.0 Hz, $^3J_{H\text{-}P}$ = 5.6Hz); ^1H NMR (D_2O) δ: 3.99 (ddd, 1H, $^3J_{H\text{-}H}$ = 11.0 Hz, $^2J_{H\text{-}P}$ = 8.0 Hz, $^3J_{H\text{-}H}$ = 2.8 Hz), 2.63 (ddd, 1H, $^2J_{H\text{-}H}$ = 15.9 Hz, $^3J_{H\text{-}P}$ = 5.5 Hz, $^3J_{H\text{-}H}$ = 2.8 Hz), 2.42 (ddd, 2H, $^2J_{H\text{-}H}$ = 16.0 Hz, $^3J_{H\text{-}H}$ = 11.0 Hz, $^3J_{H\text{-}P}$ = 7.6 Hz).

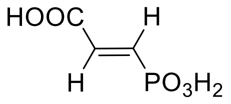

(*E*)-3-phosphonoacrylic acid (7e). ^{31}P NMR (D_2O): δ 10.65 (dd, $J_{H\text{-}P}$ = 19.6 Hz, $J_{H\text{-}P}$ = 17.8 Hz); ^{1}H NMR (D_2O) δ: 6.58 (dd, 1H, $J_{H\text{-}P}$ = 17.1 Hz, $^{3}J_{H\text{-}H}$ = 17.1 Hz), 6.30 (dd, 1H, $J_{H\text{-}P}$ = 19.6 Hz, $^{3}J_{H\text{-}H}$ = 17.4 Hz).

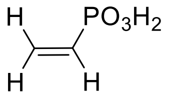

Vinylphosphonic acid (7a). ^{31}P NMR (D_2O): δ 13.11 (ddd, $^{3}J_{H\text{-}P(trans)}$ = 47.0 Hz, $^{3}J_{H\text{-}P(cis)}$ = 23.4 Hz, $^{2}J_{H\text{-}P}$ = 20.6 Hz); ^{1}H NMR (D_2O) δ: 6.02 (ddd, 1H, $^{2}J_{H\text{-}P}$ = 20.9 Hz, $^{3}J_{H\text{-}H}$ = 18.8 Hz, $^{3}J_{H\text{-}H}$ = 12.5 Hz), 5.82 (ddd, 1H, $^{3}J_{H\text{-}P(cis)}$ = 23.8 Hz, $^{3}J_{H\text{-}H}$ = 18.8 Hz, $^{2}J_{H\text{-}H}$ = 2.5 Hz), 5.71 (ddd, 1H, $^{3}J_{H\text{-}P(trans)}$ = 46.9 Hz, $^{3}J_{H\text{-}H}$ = 12.7 Hz, $^{2}J_{H\text{-}H}$ = 2.5 Hz).

Table S22. Comparison of chemical shifts and coupling constants on ^{1}H and ^{31}P NMR spectra of 3-hydroxy-3-phosphonopropanoic **(1e)** with 1-hydroxy-2-phenylethylphosphonic acid **(5g)** (ABr1236).

**Structure**	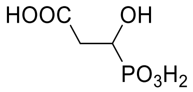	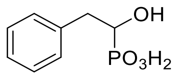
**Source**	**ABr1248**	**ABr1236**
**Solvent**	**D_2O**	**D_2O**
**^{31}P NMR**	19.07 (dt, *J* = 8.0, 5.6Hz)	18.22 (m, *J* = 6.9 Hz)
**^{1}H NMR**		7.27-7.30 (4H, m)
		7.22-7.27 (m, 1H)
	3.99 (ddd, 1H, *J* = 11.0, 8.0, 2.8 Hz)	3.77 (ddd, 1H, *J* = 7.5, 3.1, 9.6 Hz)
	2.63 (ddd, 1H, *J* = 15.9, 5.5, 2.8 Hz)	3.07 (ddd, 1H, *J* = 14.4, 3.1, 3.4 Hz)
	2.42 (ddd, 2H, *J* = 16.0, 11.0, 7.6 Hz)	2.66 (ddd, 1H, *J* = 14.4, 9.6, 7.8 Hz)

Table S23. Comparison of chemical shifts and coupling constants on ^{1}H and ^{31}P NMR spectra of the reaction products of 3-amino-3-phosphonopropanoic **(1e)** with $NaNO_2$ with compounds described in the literature.

**Structure**	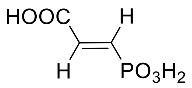		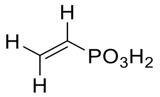	
**Source**	**ABr1248**	**Reference [22]**	**ABr1248**	**Reference [6]**
**Solvent**	**D_2O**	**D_2O (trisodium salt)**	**D_2O**	**$CDCl_3$**
**^{31}P NMR**	10.65 (dd, *J* = 19.6, 17.8 Hz)	13.88	13.11 (ddd, *J* = 47.0, 23.4, 20.6 Hz)	17.3 (ddd, *J* = 20.0, 20.5, 51.3 Hz)
**^{1}H NMR**	6.58 (t, 1H, *J* = 17.1 Hz)	7.22-7.08 (dd, 1H, *J* = 12.4, 17.1 Hz)	6.02 (ddd, 1H, *J* = 20.9, 18.8, 12.5 Hz)	6.31 (ddd, 1H, *J* = 20.5, 16.2, 4.5 Hz)
	6.30 (dd, 1H, *J* = 19.6, 17.4 Hz)	5.70-5.82 (dd, 1H, *J* = 9.0, 7.6 Hz)	5.82 (ddd, 1H, *J* = 23.8, 18.8, 2.5 Hz)	6.05 (ddd, 1H, *J* = 51.3, 11.2, 4.5 Hz),
			5.71 (ddd, 1H, *J* = 46.9, 12.7, 2.5 Hz)	6.02 (ddd, 1H, *J* = 20.0, 16.2, 11.2 Hz)

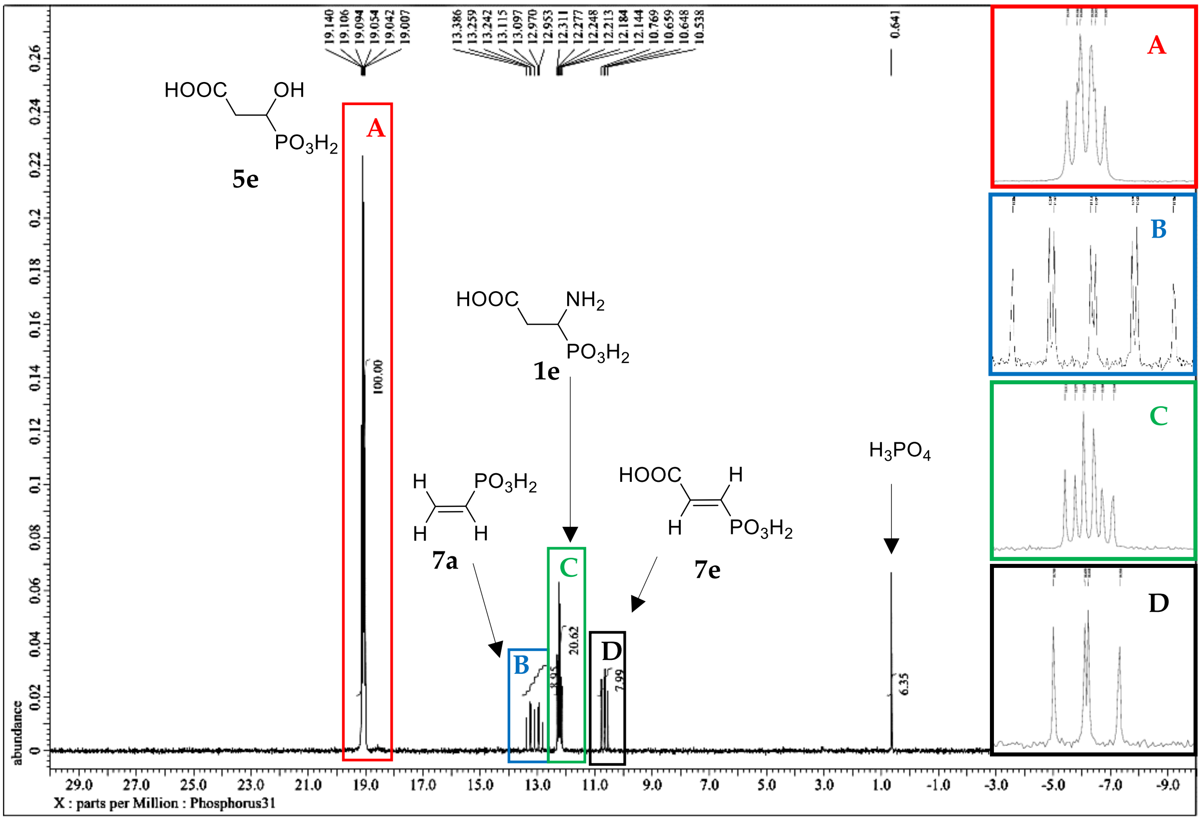

Figure S69. ^{31}P NMR (D_2O, 162MHz) spectra of the crude post-reaction mixture of 3-amino-3-phosphonopropanoic acid **(1e)** with $NaNO_2$ after 264h.

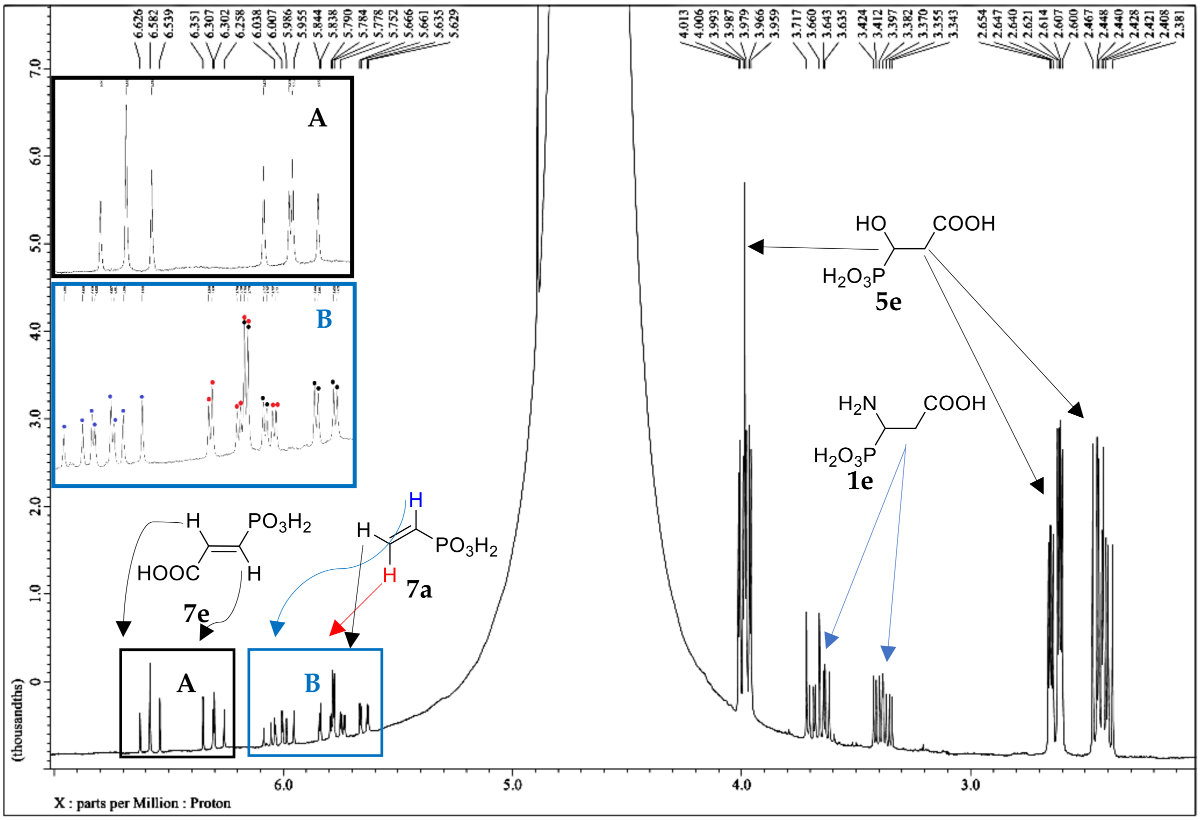

Figure S70. ^{1}H NMR (D_2O, 400MHz) spectra of the crude post-reaction mixture of 3-amino-3-phosphonopropanoic acid **(1e)** with $NaNO_2$ after 264h. .

Table S24. Comparison of structures and chemical shifts of 1-hydroxyalkylphosphonic acids **5** and 2-hydroxyalkylophosphonic acids **5′** on ^{31}P NMR spectra of crude post-reaction mixtures of 1-aminoalkylphosphonic acids with $NaNO_2$ in water.

Structure	^{31}P NMR (D_2O)	Structure	^{31}P NMR (D_2O)	Structure	^{31}P NMR (D_2O)
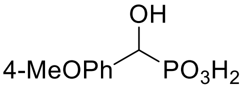	17.23 (d)	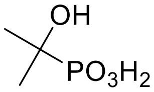	23.84 (septet)	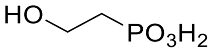	21.67 (tt)
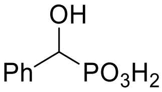	17.27 (d)	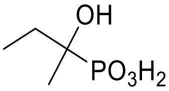	24.18 (ddq)	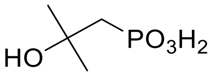	21.24 (t)
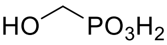	18.50 (t)	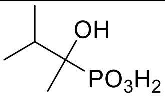	24.06 (dq)	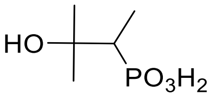	26.36 (dq)
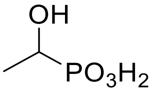	21.33 (dq)	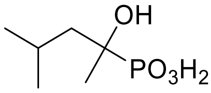	24.18 (tq)		26.44 (dq)
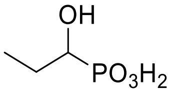	20.96 (dt)	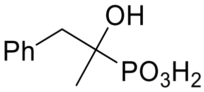	23.47 (dqq)	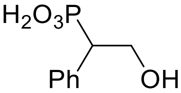	18.96 (ddd)
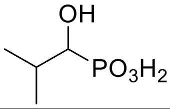	20.57 (dd)	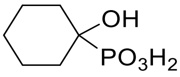	23.88 (br s)		
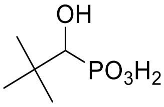	19.91 (d)	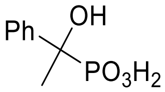	20.28 (q)		
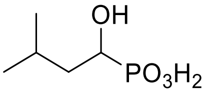	21.58 (dd)				
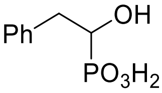	19.59 (ddd)				
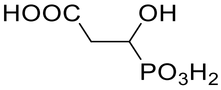	19.07 (dd)				

Table S25. Comparison of structures and chemical shifts of vinylphosphonic acid derivatives **7** and **7'**on ^{31}P NMR spectra of crude post-reaction mixtures of 1-aminoalkylphosphonic acids **1** with $NaNO_2$ in water.

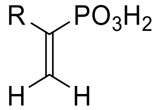	^{31}P NMR (D_2O)	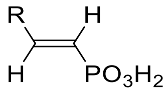	^{31}P NMR (D_2O)	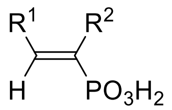	^{31}P NMR (D_2O)
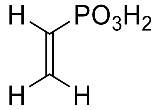	12.9 (ddd)	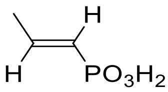	13.84 (ddq)	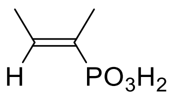	17.47 (dqq)
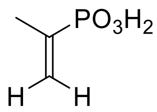	15.31 (ddq)	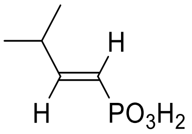	14.90 (ddd)	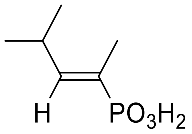	17.87 (ddq)
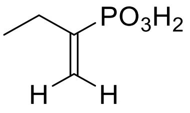	15.63 (ddt)	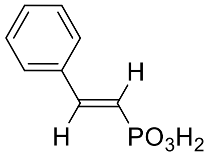	13.38 (dd)	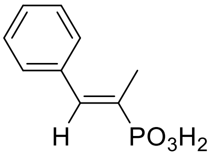	16.91 (dq)
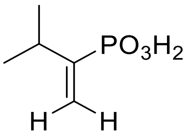	15.84 (ddd)	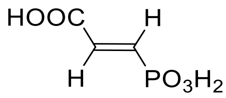	13.11 (dd)	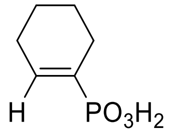	16.25 (doublet of quintets)
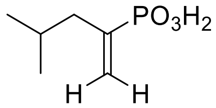	15.6 (ddt)				
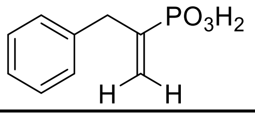	14.53 (ddt)				

4. References

1. Soroka, M. Comments on the synthesis of aminomethylphosphonic acid. *Synthesis* **1989**, *7*, 547–548.
2. Soroka, M.; Mastalerz, P. The synthesis of phosphonic and phosphinic analogs of aspartic acid and asparagine. *Rocz. Chem.* **1976**, *50*, 661–666.
3. Soroka, M. The synthesis of 1-aminoalkylphosphonic acids. A revised mechanism of the reaction of phosphorus trichloride, amides and aldehydes or ketones in acetic acid (Oleksyszyn reaction). *Liebigs Ann. Chem.* **1990**, *1990*, 331–334.
4. Goldeman, W.; Soroka, M. The preparation of dialkyl 1-hydroxyalkylphosphonates in the reaction of trialkyl phosphites with oxonium salts derived from aldehydes or ketones. *Synthesis* **2006**, 2006, 3019–3024.
5. Koppenhoefer, B.; Schuring, V. (S)-2-Chloroalkanoic acids of high enantiomeric purity from *(S)*-2-amino acids: *(S)*-2-Chloropropanoic acid. *Org. Synth.* **1988**, 66, 151–151.
6. Sainz-Diaz, C.I.; Galvez-Ruano, E.; Hernandez-Laguna, A.; Bellanato, J. Synthesis, Molecular Structure, and Spectroscopical Properties of Alkenylphosphonic Derivatives. 1. Vinyl-, Propenyl-, (Bromoalkenyl)-, and (Cyanoalkenyl)phosphonic Compounds. *J. Org. Chem.* **1995**, *60*, 74–83.
7. Fitch, S.J.; Moedritzer, K. Nuclear magnetic resonance study of the P-C(OH)-P to P-CO-P rearrangement: Tetraethyl-1-hydroxyalkylidenediphosphonates. *J. Am. Chem. Soc.* **1962**, *84*, 1876–1879.
8. Chang, W.-Ch.; Mansoorabadi, S.O.; Liu, H.-W. Reaction of HppE with Substrate Analogues: Evidence for Carbon-Phosphorus Bond Cleavage by a Carbocation Rearrangement. *J. Am. Chem. Soc.* **2013**, *135*, 8153–8156.
9. Liu, P.; Murakami, K.; Seki, T.; He, X.; Yeung, S.M.; Kuzuyama, T.; Seto, H.; Liu, H.W. Protein Purification and Function Assignment of the Epoxidase Catalyzing the Formation of Fosfomycin. *J. Am. Chem. Soc.* **2001**, *123*, 4619–4620.
10. Baltser, A.E.; Zaitsev, D.A.; Ivanova, T.V.; Babenko, T.G.; Barskova, E.N. Addition of morpholine and pyrrolidine to isopropenylphosphonic acid in situ. *Russ. J. Org. Chem.* **2013**, *49*, 627–628.
11. Blazis, V.J.; Koeller, K.J.; Spilling, Ch. D. Reactions of chiral phosphorous acid diamides: The asymmetric synthesis of chiral α-hydroxy phosphonamides, phosphonates, and phosphonic acids. *J. Org. Chem.* **1995**, *60*, 931–940.

12. Zhou, S.; Pan, J.; Davis, K.M.; Schaperdoth, I.; Wang, B.; Boal, A.K.; Krebs, C.; Bollinger, J.M. Steric enforcement of cis-epoxide formation in the radical C–O-coupling reaction by which (*S*)-2-hydroxypropyl-phosphonate epoxidase (HppE) produces Fosfomycin. *J. Am. Chem. Soc.* **2019**, *141*, 20397–20406.
13. Hudson, H.R.; Ismail, F.; Pianka, M.; Wan, Ch.-W. The formation of α-amino- and α-hydroxyalkanephosphonic acids in the reactions of phosphite esters with aldehydes and alkyl carbamates. *Phosphorus, Sulfur and Silicon and the Related Elements* **2000**, *164*, 245–257.
14. Oehler, E.; Kanzler, S. Synthesis of phosphonic acids related to the antibiotic fosmidomycin from allylic α- and γ-hydroxyphosphonates. *Phosphorus, Sulfur and Silicon and the Related Elements* **1996**, *112*, 71–90.
15. Quast, H.; Heuschmann, M. Three-membered heterocycles. 12. Synthesis of a phosphirane oxide. *Liebigs Annalen der Chemie*, **1981**, *5*, 977–992.
16. Kenyon, G.L.; Westheimer, F.H. Stereochemistry of unsaturated phosphonic acids. *J. Am. Chem. Soc.* **1966**, *88*, 3557–3561.
17. Prishchenko, A.A.; Livantsov, M.V.; Novikova, O.P.; Livantsova, L.I.; Petrosyan, V.S. Synthesis of new functionalized aryl-substituted methylphosphonic and methylenediphosphonic acids and their derivatives. *Heteroat. Chem.* **2016**, *27*, 381–388.
18. Rueppel, M.L.; Marvel, J.T. Proton and phosphorus-31P NMR spectra of substituted methylphosphonic acids with indirect determination of phosphorus-31P shifts. *Org. Magn. Reason.* **1976**, *8*, 19–20.
19. Yan, F.; Moon, S.-J.; Liu, P.; Zhao, Z.; Lipscomb, J.D.; Liu, A.; Liu, H.-W. Determination of the Substrate Binding Mode to the Active Site Iron of (S)-2-Hydroxypropylphosphonic Acid Epoxidase Using 17O-Enriched Substrates and Substrate Analogues, *Biochemistry* **2007**, *46*, 12628–12638.
20. Chen, R.; Breuer, E. Direct Approach to α-Hydroxyphosphonic and α,ω-Dihydroxyalkane-α,ω-bisphosphonic Acids by the Reduction of (Bis)acylphosphonic Acids. *J. Org. Chem.* **1998**, *63*, 5107–5109.
21. Saha, U.; Helvig, Ch. F.; Petkovich, P.M. Phosphate Management with Small Molecules. US 9198923, 1 December 2015.
22. De Macedo Puyau, P.; Perie, J.J. Synthesis Of Substrate Analogues And Inhibitors For The Phosphoglycerate Mutase Enzyme, *Phosphorus, Sulfur Silicon Relat. Elem.* **1997**, *129*, 13–45.
